# The *Legionella pneumophila* effector SidL is an adenylyltransferase that modifies the glycolytic intermediate 3-phosphoglycerate

**DOI:** 10.1016/j.molcel.2026.07.007

**Published:** 2026-07-24

**Authors:** Joshua J. Black, A. Maxwell Burroughs, Edrees H. Rashan, Charles R. Nosal, Katerina A. Romanov, Marco A. Catipovic, Catherine Valadez, L. Aravind, Kevin G. Hicks, Caren L. Freel Meyers, Tamara J. O'Connor, Matthew G. Vander Heiden, Rachel Green

**Affiliations:** (1) Department of Molecular Biology & Genetics, Johns Hopkins University School of Medicine; Baltimore, MD, USA; (2) Howard Hughes Medical Institute; Chevy Chase, MD, USA; (3) Computational Biology Branch, Division of Intramural Research, National Library of Medicine, National Institutes of Health; Bethesda, MD, USA; (4) Koch Institute for Integrative Cancer Research, Massachusetts Institute of Technology; Cambridge, MA, USA; (5) Department of Biology, Massachusetts Institute of Technology; Cambridge, MA, USA; (6) Department of Pharmacology and Molecular Sciences, Johns Hopkins University School of Medicine, Baltimore, MD, USA; (7) Chemistry-Biology Interface Graduate Training Program, Johns Hopkins University, Baltimore, MD, USA; (8) Department of Nutrition & Integrative Physiology, University of Utah College of Health; Salt Lake City, UT, USA; (9) Dana-Farber Cancer Institute; Boston, MA, USA

## Abstract

The large effector arsenal of the bacterial pathogen *Legionella pneumophila* has been a rich source of biochemistry, highlighting the immense diversity of strategies deployed in host-pathogen conflict. Here, we redefine the purported translation inhibitor SidL as an enzyme that targets a glycolytic metabolite, discovering that it adenylates 3-phosphoglycerate to produce the previously unknown molecule 2-AMP-3-phosphoglycerate. When expressed alone in mammalian cells, SidL adenylates 3-phosphoglycerate, disrupts glycolysis, and blocks the nutrient-responsive translation regulator mTORC1, which we propose indirectly causes translation inhibition. Moreover, we observe SidL-dependent production of 2-AMP-3-phosphoglycerate in macrophages during *L. pneumophila* infection, the timing of which is consistent with a role for SidL in the early stages of the infection cycle. Thus, our study uncovers a mechanism in which an intracellular pathogen uses chemical modification of a glycolytic intermediate to target central carbon metabolism in the host.

## eTOC

Black et al. identify the *L. pneumophila* effector SidL as an adenylyltransferase that targets host glycolysis. SidL covalently modifies the glycolytic intermediate 3-phosphoglycerate (3PG) with AMP, generating the previously unknown metabolite 2-AMP-3-phosphoglycerate (2-AMP-3PG) while simultaneously preventing 3PG use in eukaryotic metabolism.

## Graphical Abstract

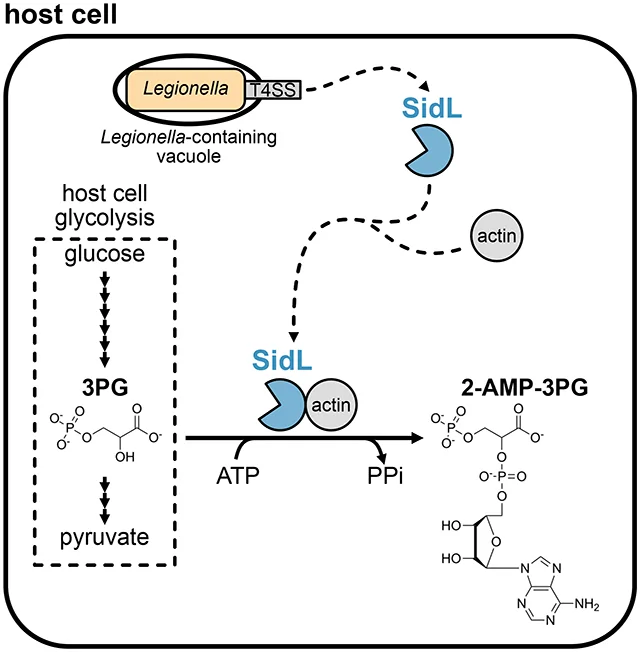

## Introduction

Biological conflicts between host organisms and pathogens drives the evolution of pathogen effectors, yielding a remarkable diversity of enzymes with specialized functions.^1^ The study of such enzymes has provided insights into how pathogens subvert host biology as well as the endogenous regulation of eukaryotic processes.^2^ One prominent example of effector diversity is found in the intracellular bacterium *Legionella pneumophila*. Its 24-hour infection cycle begins as the bacterium is phagocytosed by an amoeba or macrophage where *L. pneumophila* uses a Type IV Secretion System (T4SS) called Dot/Icm to translocate an arsenal of over 300 effectors into the host cell to generate and maintain a replicative niche.^3^ Studies of individual effectors have revealed a broad range of functions, sometimes uncovering unexpected biochemistry;^4-12^ however, roughly 75% of effectors have yet to be characterized, suggesting a trove of unexplored activities left to be uncovered.^3^

Reflecting the breadth of its effector repertoire, *L. pneumophila* targets a variety of host processes, often using multiple, redundant effectors.^13^ For example, *L. pneumophila* encodes at least eight effectors that inhibit host translation,^14-24^ including a kinase,^23^ an acetyltransferase,^24^ and four glycosyltransferases^17-19^ that modify the ribosome or translation-associated proteins. Similarly, *L. pneumophila* encodes multiple effectors that target host metabolism,^25-31^ including ADP-ribosyltransferases that inactivate host mitochondrial and metabolic proteins.^25-27^ Notably, there are many examples of enzymatic effectors that target host proteins^32^ but few that target host metabolites, such as an amylase that degrades host glycogen^31^ and phosphatases that act on phospholipid headgroups.^5,33^

The effector SidL/Ceg14 was first described as a translation-targeting effector,^14,15^ as it blocks translation in cell-free extracts and when exogenously expressed in mammalian cells.^14-17^ However, whether SidL directly targets the translation machinery was questioned when it was subsequently shown to bind actin and block its polymerization *in vitro*^16^ and, more recently, to act as an actin-dependent nucleotidase that hydrolyzes ATP into AMP and pyrophosphate.^34^ The latter function readily rationalized its ability to inhibit ATP-dependent *in vitro* reactions such as translation and actin polymerization.

Here, we independently characterized SidL and similarly find that SidL hydrolyzes ATP into AMP *in vitro*. However, rather than simply hydrolyzing ATP to generate AMP, we discover that SidL is an adenylyltransferase that modifies the glycolytic intermediate 3-phosphoglycerate (3PG) with AMP. This reaction produces a previously unknown molecule that we identify as 2-AMP-3PG. When expressed ectopically in mammalian cells, SidL produces 2-AMP-3PG, decreases 3PG abundance, and disrupts downstream glycolytic metabolism. Consistent with previous studies showing that perturbations to glycolysis inactivate the major translation regulator mTORC1 (mechanistic target of rapamycin complex 1),^35-44^ SidL expression also inhibits mTORC1 signaling which we propose is responsible for the translation shutdown in cells. Finally, we show that *L. pneumophila* infection leads to the production of 2-AMP-3PG in a SidL-dependent manner at an early stage of the infection cycle, indicating that pathogens use chemical modification of metabolites to interface with central carbon metabolism in the host cell.

## Results

### SidL inhibits translation and mTORC1 signaling in HEK293T cells.

**Figure 1.**
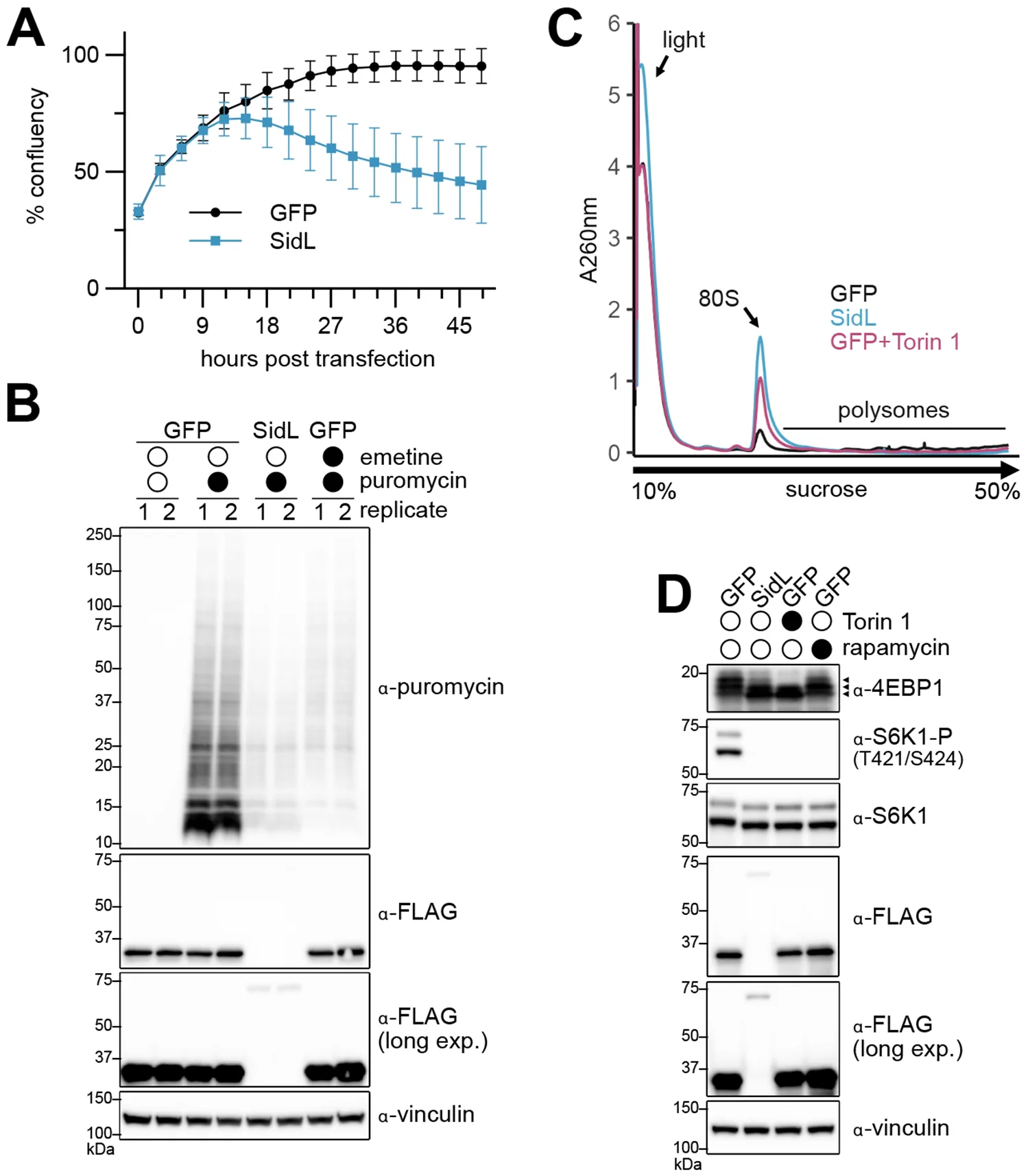
SidL inhibits translation and mTORC1 signaling in HEK293T cells. **(A)** Proliferation curves of HEK293T cells transfected with plasmids encoding 3xFLAG-GFP (pJB63) or 3xFLAG-SidL (pJB91). Values are mean ± standard deviation (SD) of biological triplicates. **(B)** Immunoblots of puromycin incorporation assays of HEK293T cells transfected for ~18-20 hours with plasmids encoding 3xFLAG-GFP (pJB63) or 3xFLAG-SidL (pJB91) prior to 18 μM puromycin treatment for 5 minutes. Treatment with 360 μM emetine for 20 minutes preceding puromycin treatment was used as a control for translation inhibition. Experiment done in technical duplicate. **(C)** Sucrose gradient traces of lysates from HEK293T cells transfected for ~18-20 hours with plasmids encoding 3xFLAG-GFP (pJB63) ± 300 nM Torin 1 or 3xFLAG-SidL (pJB91). Torin 1 treatment was started ~2 hours post transfection. **(D)** Immunoblots for mTORC1 activity in HEK293T cells transfected for ~18-20 hours with plasmids encoding 3xFLAG-SidL (pJB91) or 3xFLAG-GFP (pJB63) ± 300 nM Torin 1 or 100 nM rapamycin. Treatment with Torin 1 or rapamycin was started ~2 hours post transfection. For 4EBP1, upper, middle, and lower triangles denote hyper-phosphorylated, partially phosphorylated, and hypo-phosphorylated species, respectively. For panels B-D, closed and open circles respectively indicate the presence and absence of the indicated treatments. Panels B-D are representative of two independent experiments.

To explore SidL function in mammalian cells, we transiently transfected human embryonic kidney 293T (HEK293T) with plasmids encoding human codon-optimized N-terminally 3xFLAG-tagged SidL cells and compared it to expression of 3xFLAG-GFP. SidL-expressing cells had strongly reduced cell proliferation relative to GFP-expressing cells (Fig. 1A), indicating that SidL was active in HEK293T cells. Because SidL was first described as a translation-targeting effector,^14,15^ we first followed puromycin incorporation into nascent peptides to assay its effects on global translation.^45^ Indeed, SidL reduced puromycin incorporation equivalently to cells treated with the translation elongation inhibitor emetine^46^ (Fig. 1B).

To further explore how SidL affects translation, we collected polysome profile traces after ultracentrifuging cell lysates from GFP- or SidL-expressing cells on sucrose gradients to visualize the distribution of ribosomes. Here, we found that SidL-expressing cells showed a marked accumulation of 80S ribosomes with an associated reduction in the polysome pool (Fig. 1C). Notably, this polysome profile resembles that of cells treated with Torin 1 (Fig. 1C), a small molecule inhibitor of the signaling kinase complex mTORC1.^47^ Therefore, we assessed mTORC1 activity by tracking the phosphorylation of its substrates, 4EBP1 and S6K1, and found that, like with Torin 1 or rapamycin treatment, SidL reduced the phosphorylation of both substrates as inferred from the smaller size of normally multi-phosphorylated 4EBP1 and the loss of signal for phosphorylated S6K1 (Fig. 1D).

As mTORC1 is a major regulator of translation,^35^ the observation that SidL inhibited mTORC1 provides an explanation for how it hinders translation in HEK293T cells. However, this finding also suggests that translation inhibition may be indirect and that SidL instead targets one of the upstream pathways that feed into mTORC1 regulation.^48-50^

### SidL contains an MCF1-SHE domain necessary for its function.

**Figure 2.**
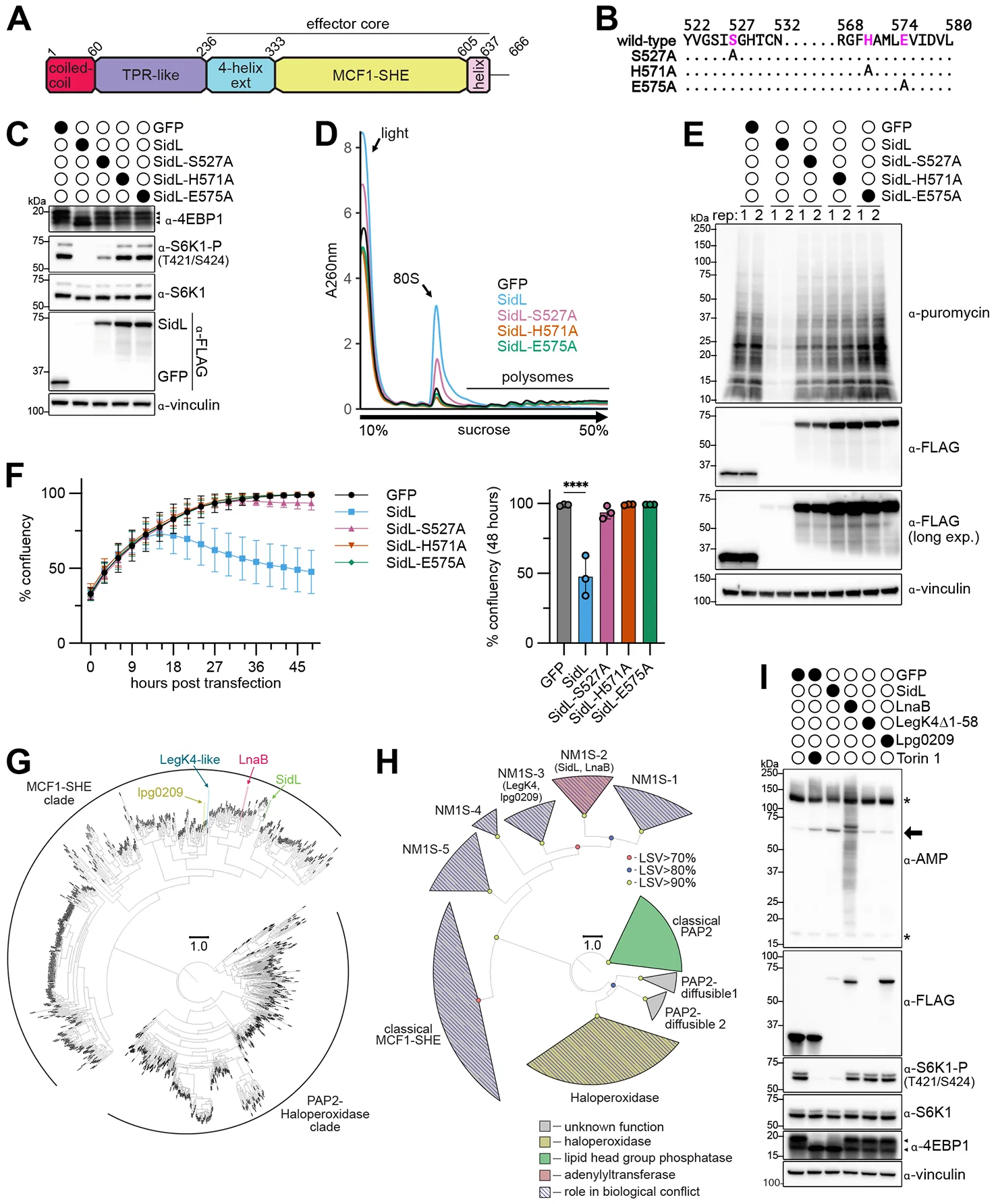
SidL-mediated effects on cells depend on a functional MCF1-SHE domain. **(A)** Domain topology of SidL. Amino acid positions delineating the domains are indicated. **(B)** Sequences surrounding conserved SHE residues (magenta) in SidL with alanine substitutions below. **(C)** Immunoblots for mTORC1 activity in HEK293T cells transfected for ~18-20 hours with plasmids encoding 3xFLAG-GFP (pJB63), 3xFLAG-SidL (pJB91), 3xFLAG-SidL-S527A (pJB92), 3xFLAG-SidL-H571A (pJB152), and 3xFLAG-SidL-E575A (pJB153). For 4EBP1, upper, middle, and lower triangles denote hyper-phosphorylated, partially phosphorylated, and hypo-phosphorylated species, respectively. **(D)** Sucrose gradient traces of lysates from HEK293T cells transfected for ~18-20 hours with plasmids encoding 3xFLAG-GFP (pJB63), 3xFLAG-SidL (pJB91), 3xFLAG-SidL-S527A (pJB92), 3xFLAG-SidL-H571A (pJB152), and 3xFLAG-SidL-E575A (pJB153). **(E)** Immunoblots of puromycin incorporation assays of HEK293T cells transfected for ~18-20 hours with plasmids encoding 3xFLAG-GFP (pJB63), 3xFLAG-SidL (pJB91), 3xFLAG-SidL-S527A (pJB92), 3xFLAG-SidL-H571A (pJB152), and 3xFLAG-SidL-E575A (pJB153) with technical duplicates. **(F)** Left: Proliferation curves of HEK293T cells transfected with plasmids encoding 3xFLAG-GFP (pJB63), 3xFLAG-SidL (pJB91), 3xFLAG-SidL-S527A (pJB92), 3xFLAG-SidL-H571A (pJB152), and 3xFLAG-SidL-E575A (pJB153). Right: Bar graph for the 48-hour timepoint. Values for both plots are mean ± SD of biological triplicates. Ordinary one-way ANOVA with multiple comparisons: *p* < 0.0001 (****) for the 48-hour timepoint. **(G)** Phylogenetic tree of the conserved core of the PAP2-Haloperoxidase-MCF1-SHE superfamily (see Fig. S1), sequences identified at tree leaves by NCBI accession number are listed in Table S1. Scale bar (in the tree’s center) indicates the average number of substitutions per site. *Legionella* effectors containing the MCF1-SHE domain are indicated. See Supplemental Data S1 for Newick tree file. **(H)** Collapsed phylogenetic tree from (F) depicting higher-order relationships between identified families, with color-coded circles indicating their support as measured by bootstrap-like Local Support Values (LSV) (see STAR Methods). The five newly identified families in the MCF1-SHE clade are labeled as NM1S (Novel> MCF1-SHE) numbers 1-5. Cartoons representing the families are filled according to known functional annotations and known or predicted roles in biological conflict^1^ (see Fig. S1F). **(I)** Immunoblots for protein adenylation and mTORC1 activity in HEK293T cells transfected for ~18-20 hours with plasmids encoding 3xFLAG-GFP (pJB63) ± 300 nM Torin 1, 3xFLAG-SidL (pJB91), 3xFLAG-LnaB (pJB224), 3xFLAG-LegK4Δ1-58 (pJB225), or 3xFLAG-Lpg0209 (pJB226). A LegK4 variant lacking amino acids 1-58 (LegK4Δ1-58) was used as it enhances solubility compared to the full-length protein while retaining functionality.^23^ Arrow denotes the ~70 kDa species that changes in response to SidL or Torin 1. Asterisks (*) denotes other species. Torin 1 treatment was started ~2 hours post transfection. For 4EBP1, the upper and lower triangles denote hyper-phosphorylated and hypo-phosphorylated species, respectively. For panels C, E, and I, closed and open circles respectively indicate the presence and absence of the indicated plasmids or treatments. Panels C, D, E, and I are representative of two independent experiments. See Fig. S1, Table S1, and Supplemental Data S1.

Because SidL lacks functional annotation in proteomic databases, we used computational sequence and structure analyses to gain insight into its evolutionary relationships, finding that SidL contains an N-terminal highly charged coiled-coil region, a run of helical repeats related to Tetratricopeptide repeats (TPR), and a Makes Caterpillars Floppy-Serine-Histidine-Glutamate (MCF1-SHE) domain^51^ followed by an extended C-terminal α-helix with an unstructured tail (Fig. 2A). The MCF1-SHE domain was first described as an enzymatic domain of unknown function found in secreted toxins and is named based on the presence of a putative catalytic triad composed of serine, histidine, and glutamate residues (SHE).^51,52^ The SHE residues in SidL are S527, H571, and E575 (Fig. 2B) and a structural prediction of SidL with AlphaFold3^53^ (Fig. S1A) positions them in close proximity, further suggesting they comprise a catalytic triad (Fig. S1B).

To test the importance of the SHE residues for the function of SidL, we generated three SidL variants each with an alanine substitution at one of the SHE residues (Fig. 2B). Expression of these SHE variants in HEK293T cells revealed that they failed to inhibit mTORC1 signaling (Fig. 2C), translation (Figs. 2D-E), and cell proliferation (Fig. 2F). Importantly, this loss-of-function was not explained by reduced protein levels; instead, the SHE variants were expressed at higher levels than wild-type SidL (Figs. 2C and 2E), a common phenotype for translation-inhibiting proteins whose translation become repressed by their own activities.^20,54^ Collectively, these data indicate that a functional MCF1-SHE domain is required for SidL activity in cells.

### Unification of SidL with a phosphotransferase and phosphatase superfamily.

Our sequence and structure similarity analysis also identified higher-order relationships amongst MCF1-SHE proteins, revealing six distinct clades: the classical MCF1-SHE clade and five additional, rapidly evolving Novel MCF1-SHE (NM1S) clades (Figs 2G-H and S1C-E). These clades feature lineage-specific sequence, structure, and domain architectural features, suggesting potentially distinct functional and substrate specificities. In addition to SidL, *L. pneumophila* encodes three other effectors, LnaB, LegK4 and Lpg0209, that contain MCF1-SHE domains with SidL being more closely related to LnaB in clade NM1S-2 than to LegK4 and Lpg0209 in NM1S-3 (Figs. 2G-H and S1F). Interestingly, LnaB is a protein-targeting adenylyltransferase, using the SHE domain to transfer the adenosine monophosphate (AMP) moiety from ATP onto host proteins with the concomitant release of pyrophosphate.^11,12^ These observations prompted us to ask if SidL, LegK4, and Lpg0209 similarly adenylate proteins.

To test this hypothesis, we expressed these proteins in HEK293T cells and monitored protein adenylation by immunoblotting cell lysates with an α-AMP antibody.^55^ As reported,^12^ LnaB expression led to the adenylation of many proteins (Fig. 2I); however, LegK4 and Lpg0209 did not, suggesting that this is not a universal activity of MCF1-SHE domain proteins under these conditions. In comparison, SidL increased the adenylation of a single ~70 kDa protein; however, because this species also increased in response to Torin 1, we suspect that its appearance results from mTORC1 inhibition rather than from direct modification by SidL. Notably, we also found that while SidL inhibited mTORC1, LnaB, LegK4, and Lpg0209 did not (Fig. 2I). Thus, despite these four effectors all containing MCF1-SHE domains, only LnaB seems to adenylate eukaryotic proteins and only SidL blocks mTORC1 signaling in this simplified context. These observations, consistent with the above phylogenetic analysis indicating diversity amongst MCF1-SHE proteins, suggest an alternative activity or substrate for SidL.

We further identified a distant relationship between the MCF1-SHE domain and the phosphatidic acid phosphatase type 2 (PAP2) superfamily (see STAR Methods, Figs. 2G-H and S1C).^56,57^ Archetypal PAP2 family members are integral membrane enzymes that catalyze phosphoryl transfer and phosphatase reactions, acting on metabolites in housekeeping processes like lipid metabolism,^58-60^ blood glucose homeostasis,^61,62^ and bacterial cell wall synthesis.^63^ Structural comparisons reveal a unified fold with a conserved alpha-helical enzymatic core, which includes the catalytic SHE residues (Figs. S1D-E). Interestingly, several diffusible PAP2 clades have evolved in addition to the known haloperoxidase clade^57,64^ and MCF1-SHE clades unified here (Figs. 2H and S1C), indicated by the absence of a hydrophobic N-terminal helix (Figs. S1D-E). Contextual genome analysis of these diffusible proteins points to potential roles in biological conflicts with other domains containing known toxin functions^1^ (Fig. S1F). These observations parallel the recently described Lipocone superfamily, in which numerous diffusible toxins and effectors evolved from an originally membrane-bound enzymatic scaffold.^65^ This relationship with the PAP2 superfamily suggests that SidL and, more broadly, other MCF1-SHE proteins catalyze phosphoryl transfer or chemically similar reactions potentially beyond just protein substrates.

### Actin binding promotes SidL activity in cells.

The activities of LnaB and SidL are stimulated by binding to the eukaryotic-specific cytoskeletal protein actin, using it as a co-factor to restrict activation to the host environment.^11,12,34^ Consistent with this, we observed specific actin enrichment in affinity purifications of SidL-H571A from HEK293T cell lysates (Fig. S2A). We used AlphaFold3^53^ to model SidL and actin together, predicting two interaction interfaces with one between actin and the N-terminal region of SidL and another with the C-terminal helix of SidL that follows its MCF1-SHE domain (Fig. S2B). This latter interaction is similar that of actin and LnaB (Fig. S2B).^11,12^

To dissect this interaction, we assayed four truncated SidL variants based on the AlphaFold3 model (Fig. S2C). First, we generated two N-terminal SidL variants: a truncation of the first 60 residues (SidL-ΔN60), which are not predicted to bind actin, and another larger truncation of the first 236 residues (SidL-ΔN236) that includes an actin-binding interface (Fig. S2C). The SidL-ΔN60 variant co-purified with actin while SidL-ΔN236 did not (Fig. S2D), supporting the prediction that residues 61-236 interface with actin. Next, we generated two truncations from the C-terminus of SidL (Fig. S2C): a truncation of the terminal 28 residues (SidL-ΔC28), which includes the disordered tail, and another truncation of the last 43 residues (SidL-ΔC43), which contains the second predicted SidL-actin interface (Fig. S2C). The SidL-ΔC28 variant co-purified actin while the SidL-ΔC43 variant did not, supporting the requirement of this second region for actin binding (Fig. S2D). Notably, only the two variants that co-purify with actin inhibited mTORC1 and translation (Figs. S2E-F). Together, these data indicate that actin binding is necessary for the *in vivo* effects of SidL, consistent with actin being important for its *in vitro* activation.^34^

### SidL hydrolyzes ATP into AMP *in vitro*.

**Figure 3.**
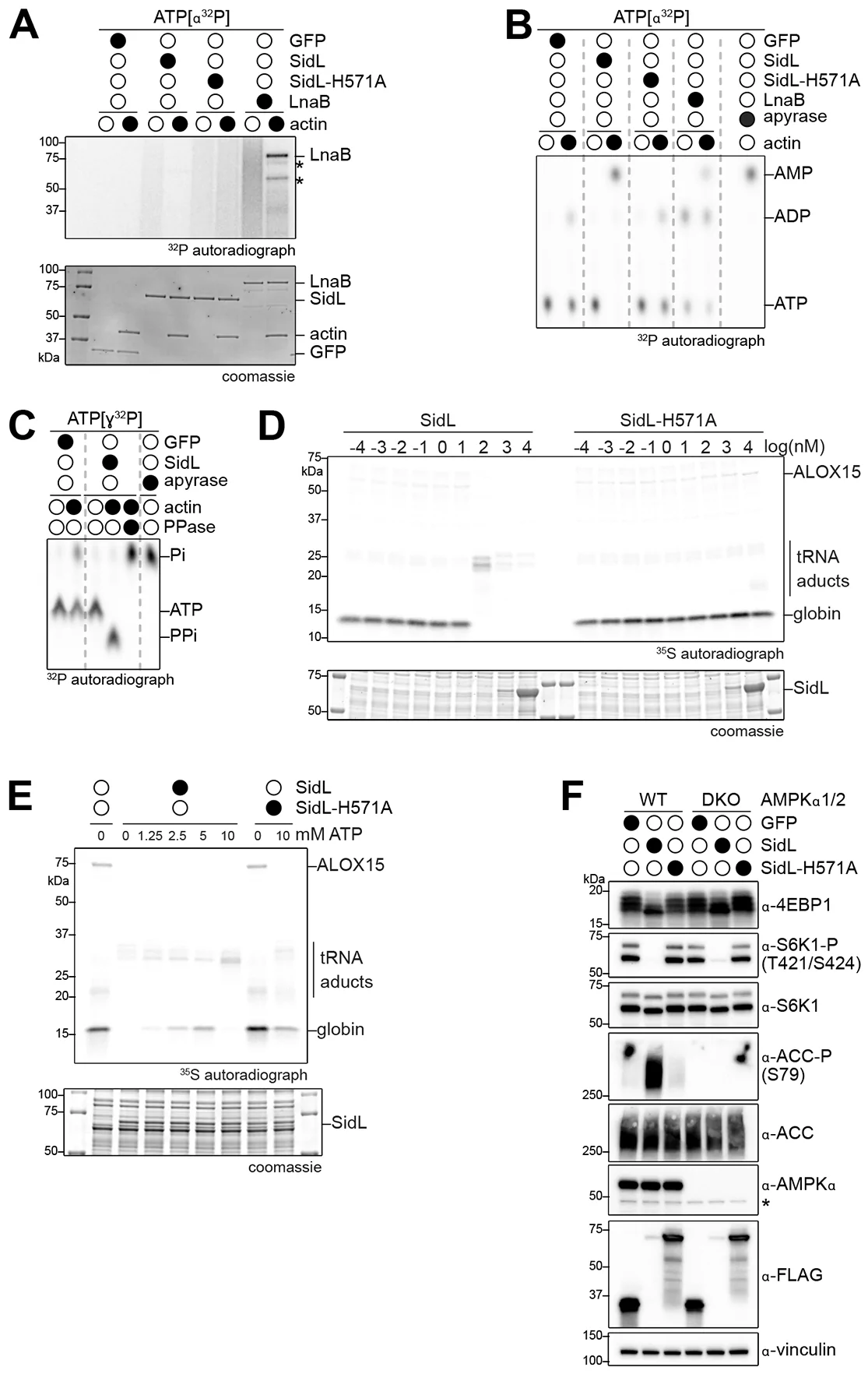
SidL hydrolyzes ATP to produce AMP and pyrophosphate *in vitro*. **(A)** Protein auto-adenylation reactions containing ATP[α^32^P] with indicated recombinant proteins ± human actin were separated by SDS-PAGE then analyzed by Coomassie staining and autoradiography. GFP, SidL, and SidL-H571A are N-terminally 3xFLAG-tagged and LnaB is N-terminally GST-3C-3xFLAG-tagged. Asterisks (*) denote adenylated protein contaminants in the LnaB preparation. **(B)** Analysis of ATP[α^32^P] hydrolysis by 3xFLAG-SidL ± actin compared to 3xFLAG-GFP ± actin, 3xFLAG-SidL-H571A ± actin, GST-3C-3xFLAG-LnaB ± actin, and apyrase as analyzed by TLC separation using a 500 mM KH_2_PO_4_ pH 3.5 mobile phase. **(C)** ATP[ɣ^32^P] hydrolysis by 3xFLAG-SidL ± pyrophosphatase (PPase) compared to 3xFLAG-GFP and apyrase as analyzed by TLC with 1.5 M KH_2_PO_4_ pH 3.4 mobile phase. **(D)** *In vitro* translation reactions supplemented with methionine[^35^S] performed in the presence of a titration of 3xFLAG-SidL or 3xFLAG-SidL-H571A. Newly translated globin and lipoxygenase (ALOX15) proteins were separated by SDS-PAGE and visualized by autoradiography. The ~25 kDa species represent charged tRNA and tRNA-protein adducts. Total protein loading is visualized by Coomassie staining. **(E)** As in (D) but with 500 nM recombinant proteins and the indicated ATP supplementation. **(F)** Immunoblots for mTORC1 and AMPK activity in wild-type or AMPKα1/2-DKO HEK293T cells transfected for ~18-20 hours with plasmids encoding 3xFLAG-GFP (pJB63), 3xFLAG-SidL (pJB91), or 3xFLAG-SidL-H571A (pJB152). For 4EBP1, upper, middle, and lower triangles denote hyper-phosphorylated, partially phosphorylated, and hypo-phosphorylated species, respectively. Asterisks (*) denote non-specific species. For panels A, B, C, E and F, closed and open circles respectively indicate the presence and absence of the indicated proteins or plasmids. Each panel is representative of two independent experiments.

To further explore SidL activity *in vitro*, we purified recombinant SidL and the catalytically inactive variant SidL-H571A as well as GFP and LnaB from *Escherichia coli* for *in vitro* biochemistry studies. Because LnaB undergoes auto-adenylation,^11,12^ we first asked if SidL similarly auto-adenylates. To test this, we individually incubated SidL, LnaB, and GFP with ATP radiolabeled at the α-phosphate (ATP[α^32^P]) with or without actin then separated them by SDS-PAGE and used autoradiography to assess their potential modification with AMP[α^32^P]. We observed robust actin-dependent auto-adenylation for LnaB but not for SidL (Fig. 3A), consistent with the lack of protein adenylation activity for SidL in cells (Fig. 2I).

We then used aqueous thin-layer chromatography (TLC) on polyethyleneimine-cellulose to analyze these reactions and found that SidL hydrolyzed ATP[α^32^P] into a fast-migrating, less polar product in an actin-dependent manner (Fig. 3B). Importantly, this requires the catalytic activity of SidL as the SidL-H571A variant did not hydrolyze ATP. The hydrolyzed product does not co-migrate with ADP (produced by actin) indicating that it is not ADP but instead co-migrated with AMP (produced by apyrase), indicating SidL hydrolyzes ATP to AMP. We next incubated SidL with ATP radiolabeled at the gamma phosphate (ATP[ɣ^32^P]) to test if SidL successively removes phosphate (Pi) from ATP (like apyrase) or if SidL cleaves the bond between the alpha and beta phosphates to release AMP and pyrophosphate (PPi). This reaction produced a slow-migrating, more polar species that was converted into Pi by adding pyrophosphatase (Fig. 3C). Together, these data indicate that SidL hydrolyzes ATP to AMP and PPi *in vitro*.

Our findings are consistent with a recent study that proposed SidL is an actin-dependent ATPase that hydrolyzes ATP into AMP^34^ and readily explains its ability to hinder ATP-dependent reactions such as translation^16,17^ and actin polymerization *in vitro*.^16,34^ Indeed, we saw that wild-type SidL but not SidL-H571A hinders *in vitro* translation reactions (Fig. 3D), which was partially offset by ATP supplementation (Fig. 3E). Notably, AMP-activated protein kinase (AMPK) negatively regulates mTORC1 in response to elevated AMP levels.^66^ Thus, SidL-mediated increases in cellular AMP levels could explain how SidL inactivates mTORC1. Consistent with this notion, we found that the presence of SidL led to increased phosphorylation of the AMPK substrate acetyl-CoA carboxylase (ACC) (Fig. 3F). However, dual disruption of the genes encoding the catalytic subunit of AMPK (AMPKα1/2 DKO) did not restore mTORC1 signaling in SidL-expressing cells despite ACC phosphorylation being fully abolished (Fig. 3F). Thus, AMP production is not the primary driver of mTORC1 inactivation in response to SidL, suggesting an alternative biochemical activity.

### SidL uses ATP to modify the small molecule 3-phosphoglycerate *in vitro*.

**Figure 4.**
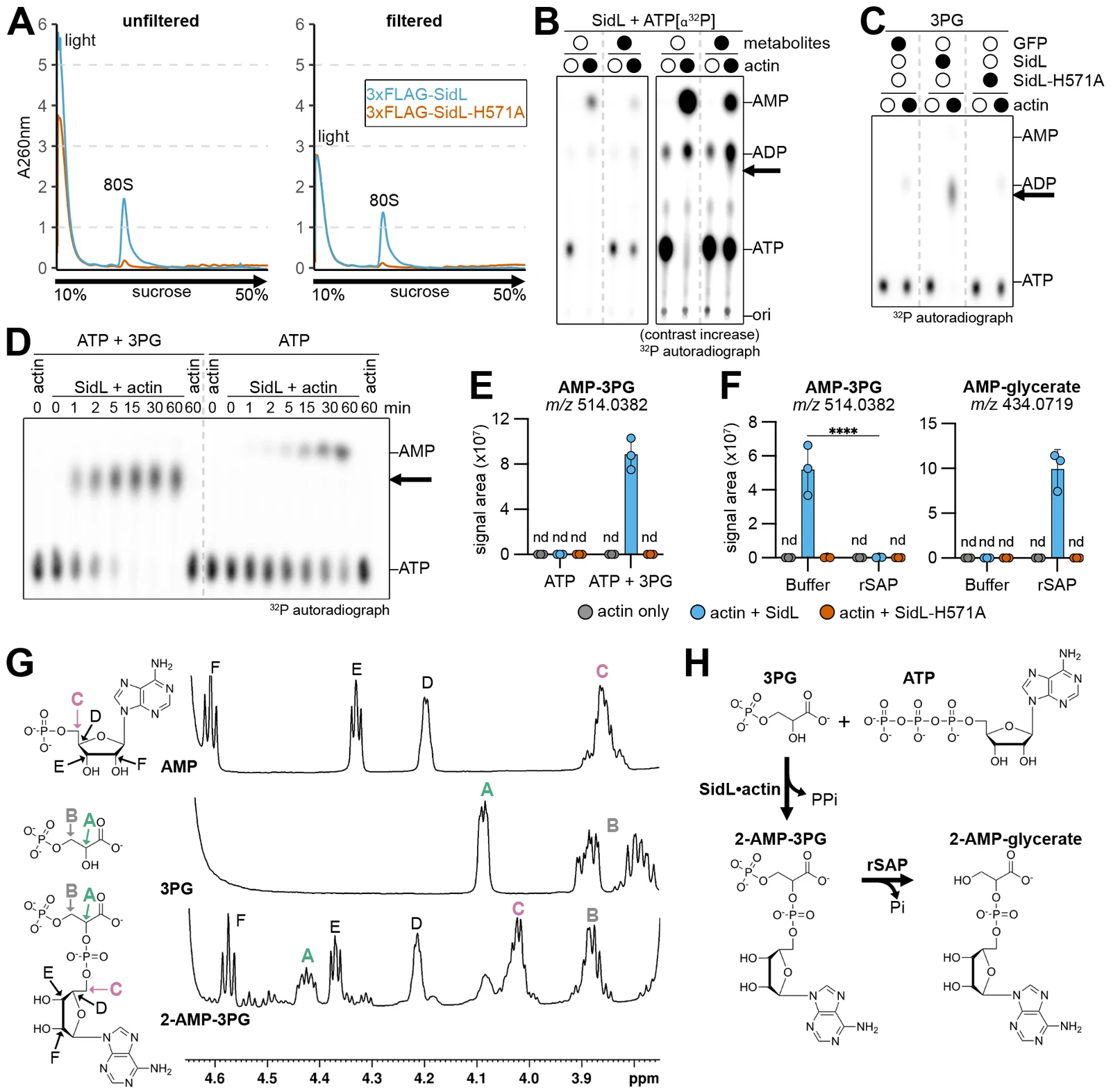
SidL adenylates the glycolytic intermediate 3-phosphoglycerate *in vitro*. **(A)** Sucrose gradient traces of lysates from HEK293T cells transfected for ~18-20 hours with plasmids encoding 3xFLAG-SidL (pJB91) or 3xFLAG-SidL-H571A (pJB152). Half of each lysate was either filtered through a G-25 desalting column (MWCO < ~5 kDa) or left unfiltered prior to ultracentrifugation. **(B)** TLC analysis of *in vitro* reactions containing 3xFLAG-SidL ± actin, ATP[α^32^P], and a 696-metabolite library (see Table S3).^67^ Black arrow indicates unique actin-dependent species; the right panel is the same as the left panel but with increased contrast. **(C)** *In vitro* [α^32^P]-modification of 3PG by 3xFLAG-SidL compared to 3xFLAG-GFP and 3xFLAG-SidL-H571A ± actin as analyzed by TLC. **(D)** Kinetic analysis of ATP[α^32^P] consumption by 3xFLAG-SidL with reactions containing 2 mM unlabeled ATP ± 20 mM 3PG. Reactions were incubated at 37°C and quenched with 10% trichloroacetic acid at the indicated timepoints. TLCs were separated with 500 mM KH_2_PO_4_ pH 3.5 mobile phase. **(E, F)** LC-MS signal intensities for AMP-3PG and AMP-glycerate from *in vitro* reactions containing ATP and actin alone or with 3xFLAG-SidL or 3xFLAG-SidL-H571A with or without 3PG **(E)** and reactions subsequently treated with rSAP or its storage buffer **(F)**. Mass-to-charge ratio (*m/z*) of each metabolite is indicated. Bars are mean ± SD with points representing individual reactions (n=3). Two-way ANOVA with multiple comparisons: *p* ≤ 0.0001 (****) for (F). “nd” denotes not detected. See Figs. S4B and S4C and Table S4. **(G)** 1D ^1^H-NMR (500 MHz, D_2_O) comparison of AMP, 3PG, and the SidL product (2-AMP-3PG) (see STAR Methods). Letters above each resonance correspond to the indicated hydrogens in the adjacent structures. See Supplemental Data S2 for additional NMR confirmation and characterization of 2-AMP-3PG. **(H)** Molecular depiction of 3PG adenylation by SidL and subsequent dephosphorylation by rSAP. For panels B and C, closed and open circles respectively indicate the presence and absence of the metabolite library or indicated proteins. Panels A-D are each representative of two independent experiments. See Figs. S3, S4, S5, and S6 and Tables S3 and S4 and Supplemental Data S2.

One hint for SidL activity came from its effects on polysome profiles in HEK293T cells. In addition to changing the distribution of ribosomes, SidL caused an increase in the UV absorbance (A260) signal in the light region of the polysome profile (Fig. 1C), which contains a mixture of low molecular weight RNAs, proteins, and metabolites. This increased signal was not present with the SHE variants (Fig. 2D) or variants that did not bind actin (Fig. S2F), indicating that it depends on SidL activity. Notably, this increase was not due to mTORC1 inhibition, as treating cells with Torin 1 did not induce this effect (Fig. 1C). Furthermore, passing lysates from SidL-expressing cells through a desalting column prior to ultracentrifugation reduced this A260 signal to that of cells expressing SidL-H571A (Fig. 4A), suggesting the accumulation of a small UV-absorbing molecule in the presence of SidL.

Because SidL hydrolyzes ATP (Fig. 3B), is related to metabolite-targeting PAP2 enzymes (Figs. 2G-H), and inhibits the metabolite-sensing mTORC1 (Fig. 1D), we hypothesized that this increased A260 signal is due to the production of an ATP-modified metabolite. To test this, we leveraged our *in vitro* assays, incubating SidL with ATP[α^32^P] and a pooled library of 696 metabolites (Table S3),^67^ with or without actin. The addition of this library reduced the hydrolysis of ATP to AMP but also resulted in the actin-dependent production of a unique species (Fig. 4B). Notably, this new species (black arrow) was slightly more polar than ADP, as reflected by its slower migration on the TLC plate. These data suggest that SidL catalyzes transfer of the α-phosphate group of ATP to an unknown metabolite.

While this [α^32^P]-modified species was a minor component in these conditions (Fig. 4B), we pursued its identification. To this end, we iteratively sub-pooled the metabolite library and screened for the generation of the SidL-catalyzed product. SidL robustly modified compounds in two of the initial sub-pools of ~24 metabolites (Fig. S3A) and further sub-pooling identified the common substrate to be 3-phosphoglycerate (3PG) (Figs. S3B and S3C). Importantly, we found that 3PG modification by SidL was actin-dependent and required SidL catalytic activity (Fig. 4C). Moreover, this is not a general activity of MCF1-SHE domain enzymes as LnaB did not modify 3PG (Fig. S3D). Kinetic analysis of the SidL reaction revealed that the rate of ATP consumption was markedly higher in the presence of excess 3PG (Fig. 4D). Consistent with these results, 3PG supplementation potentiated the *in vitro* translation inhibition of an otherwise sub-inhibitory concentration of wild-type SidL (Fig. S3E). Thus, 3PG modification is the preferred activity of SidL whereas AMP production is a basal activity that occurs in the absence of 3PG substrate.

### SidL adenylates 3PG *in vitro* to produce 2-AMP-3PG.

Considering its similarities to LnaB, an adenylyltransferase,^11,12^ we hypothesized that the SidL product was an adenylated derivative of 3PG (AMP-3PG) (Fig. S4A). Thus, we analyzed the *in vitro* SidL reactions by liquid chromatography-mass spectrometry (LC-MS) using a targeted single ion monitoring method that distinguishes and measures the abundance of the SidL product, its precursors, ATP and 3PG, and the intermediate AMP. This approach revealed that SidL produced a molecule with a mass-to-charge ratio (*m/z*) of 514.0382 (Fig. 4E), matching the expected *m/z* for the [M-H]^−^ product of AMP-3PG. Importantly, this product was only detected in reactions containing wild-type SidL and 3PG (Fig. 4E). Consistent with the kinetics data (Fig. 4D), SidL consumed more ATP in the presence of 3PG than in absence of 3PG without producing a concomitant amount of AMP (Fig. S4B). Thus, SidL uses ATP to adenylate 3PG and generate AMP-3PG.

There are three functional groups on 3PG that SidL could in principle adenylate (Fig. S4A): the 1-position carboxyl to produce 1-AMP-3PG (Fig. S4Ai), the 2-position hydroxyl to produce 2-AMP-3PG (Fig. S4Aii), or the 3-position phosphoryl to produce 3-AMP-glycerate (Fig. S4Aiii). We reasoned that the 3-position phosphoryl group in 1-AMP-3PG or 2-AMP-3PG would be susceptible to dephosphorylation by recombinant shrimp alkaline phosphatase (rSAP), producing 1- or 2-AMP-glycerate (Figs. S4Ai-ii), while the phosphoryl group of 3-ADP-glycerate would be protected from rSAP activity (Fig. S4Aiii). To test this, we incubated SidL with ATP and 3PG before splitting the reaction in half and adding either rSAP or buffer, finding that rSAP depleted AMP-3PG and produced new molecule with a *m/z* of 434.0719 (Figs. 4F and S4C). This change in *m/z* indicates the loss of a phosphate from AMP-3PG producing its dephosphorylated derivative AMP-glycerate (Figs. S4Ai-ii). Moreover, TLC analysis of analogous reactions containing SidL, 3PG, and ATP[α^32^P] showed that rSAP treatment generated a species that migrates faster than AMP-3PG but slower than Pi, consistent with the removal of a single phosphate from AMP-3PG to produce AMP-glycerate (Fig. S4D). Together, these data establish that SidL modifies 3PG at either its 1-position carboxyl or its 2-position hydroxyl (Figs. S4Ai-ii).

While the adenylation of carboxyl groups is common in biosynthetic reactions, the resulting bonds are short-lived.^68^ We reasoned that these products are unlikely to accumulate in SidL reactions and thus surmise that SidL modifies 3PG at its 2-position hydroxyl. To explore this experimentally, we characterized the ability of SidL to use ATP[α^32^P] to modify metabolites that share features with 3PG (Fig. S5A). We found that while 3PG was the most robustly modified metabolite of those tested (Fig. S5B), SidL weakly modified several others, including 2-phosphoglycerate (2PG), indicating some promiscuity for structurally similar metabolites under these conditions. Notably, SidL modified glyceraldehyde 3-phosphate, glycerol 3-phosphate, glycerol 2-phosphate, and ribulose-5-phosphate (Fig. S5B). These four metabolites contain hydroxyl groups but lack carboxyl groups indicating that SidL modifies hydroxyl groups. Interestingly, SidL did not modify glycerate (Fig. S5B), which lacks a phosphoryl group (Fig. S5A), nor did it modify glyceraldehyde or glycerol (Fig. S5B), which lack both carboxyl and phosphoryl groups (Fig. S5A). These observations suggest that SidL modifies the 2-position hydroxyl group on 3PG to produce 2-AMP-3PG (Fig. S4Aii) and that its carboxyl and phosphoryl groups contribute to the overall recognition of 3PG by SidL.

To validate the proposed structure of 2-AMP-3PG, we analyzed the reaction species by 1D ^1^H nuclear magnetic resonance (^1^H-NMR) spectroscopy. Comparison of the ^1^H-NMR spectra of AMP and 3PG to that of 2-AMP-3PG revealed several notable changes in resonances among these molecules (Fig. 4G). First, the chemical shift of the resonance of proton A that is connected to the 2-position carbon in 2-AMP-3PG was found to be >0.3 ppm downfield of the equivalent proton in 3PG, indicating the presence of a proximal group capable of de-shielding the proton. This proton resonance appeared as a quintuplet in the product distinct from the doublet of doublets observed for the equivalent proton in 3PG, a phenomenon which can be explained by its proximity to the phosphoryl group of the AMP moiety. Second, the chemical shift of the methylene proton resonance of AMP (labeled as C) in 2-AMP-3PG was found to be >0.1 ppm downfield of the corresponding resonance in AMP. Finally, we observed a collapse of the resonance from the methylene protons in 3PG (labeled as B) from a doublet of multiplets into a single multiplet in 2-AMP-3PG without a significant change in the chemical shift. These changes in chemical shifts and splitting patterns are consistent with the SidL product being 2-AMP-3PG and are further supported by additional 1D ^13^C-NMR, ^31^P-NMR and 2D NMR correlation analyses (Supplemental Data S2). Thus, SidL adenylates 3PG to produce 2-AMP-3PG (Fig. 4H).

### Adenylation of 3PG by SidL disrupts glycolysis in HEK293T cells.

**Figure 5.**
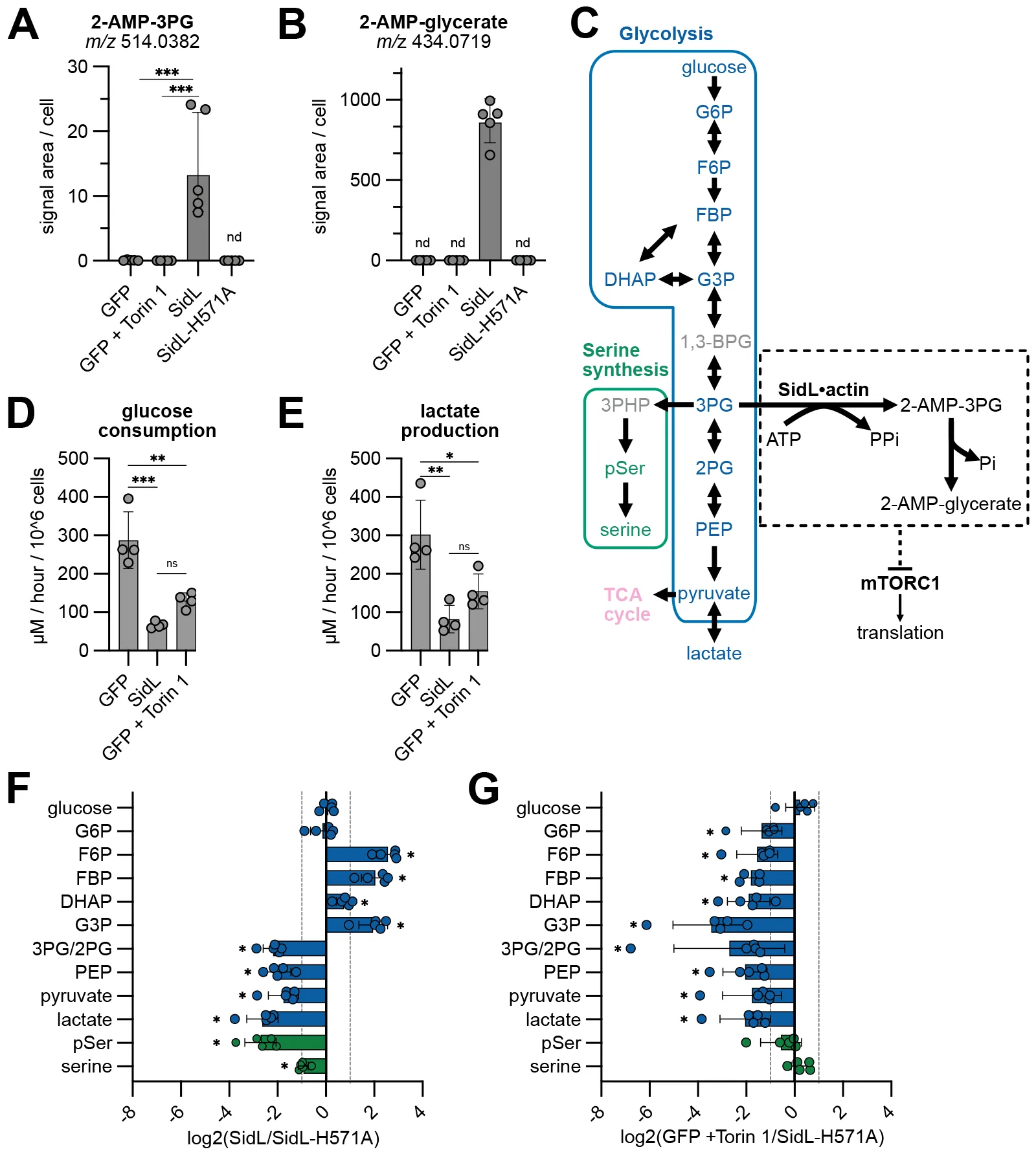
SidL adenylates 3-phosphoglycerate and perturbs glycolysis in HEK293T cells. **(A, B)** LC-MS detection of 2-AMP-3PG **(A)** and 2-AMP-glycerate **(B)** abundances in HEK293T cells transfected for ~18 hours with plasmids encoding 3xFLAG-GFP (pJB63) ± 300 nM Torin 1, 3xFLAG-SidL (pJB91), or 3xFLAG-SidL-H571A (pJB152). Torin 1 treatment was started 2 hours post transfection. Signals are normalized to cell counts; bars are mean ± SD with points representing individual replicates (n=5). *m/z* of each metabolite is indicated. Ordinary one-way ANOVA with multiple comparisons: *p* ≤ 0.001 (***). “nd” denotes not detected. **(C)** Overview of glycolysis with its intermediates (blue) and its intersection with serine synthesis (green), TCA cycle (pink), and SidL and a model for how sequestration of 3PG via its adenylation could lead to mTORC1 inactivation. 1,3-BPG and 3PHP (gray) were not detected in our LC-MS analyses. Abbreviations: G6P, glucose 6-phosphate; F6P, fructose 6-phosphate; FBP, fructose 1,6-bisphosphate; G3P, glyceraldehyde 3-phosphate; DHAP, dihydroxyacetone phosphate; 1,3-BPG, 1,3-bisphosphoglycerate; 3PG, 3-phosphoglycerate; 2PG, 2-phosphoglycerate; PEP, phosphoenolpyruvate; TCA, tricarboxylic acid cycle; 3PHP, 3-phosphohydroxylpyruvate; pSer, phosphoserine. **(D, E)** Rates of glucose consumption **(D)** and lactate production **(E)**, as determined by quantifying glucose and lactate in the medium of cells transfected with plasmids encoding 3xFLAG-GFP (pJB63) ± 300 nM Torin 1 or 3xFLAG-SidL (pJB91) between two time points (see STAR Methods). Bars are mean ± SD of biological replicates (n=4) with points being the mean of technical duplicates. Ordinary one-way ANOVA with multiple comparisons: not significant (ns), *p* ≤ 0.05 (*), *p* ≤ 0.01 (**), *p* ≤ 0.001 (***). **(F, G)** Bar graphs showing fold changes in the abundance of glycolysis and serine biosynthesis intermediates from cells transfected with plasmids encoding 3xFLAG-SidL (pJB91) **(F)** or 3xFLAG-GFP (pJB63) + 300 nM Torin 1 **(G)** compared to cells with 3xFLAG-SidL-H571A (pJB152). Cells were transfected for 18 hours and Torin 1 treatment started 2 hours post transfection. Bars are mean ± SD with points being individual replicates (n=5). Asterisks (*) indicate *p*-value ≤ 0.05 as calculated in Figs. S7B-C. Abbreviations are same as (C). See Figs. S6 and S7 and Table S5.

We next sought to determine whether SidL adenylates 3PG in HEK293T cells. Using our previously validated targeted LC-MS method for detecting 2-AMP-3PG and 2-AMP-glycerate (Figs. 4E-F), we analyzed metabolites extracted from HEK293T cells expressing SidL, SidL-H571A, or GFP with or without Torin 1 treatment and only detected the parent ions corresponding to 2-AMP-3PG and 2-AMP-glycerate in the SidL-expressing cells (Figs. 5A-B). Importantly, the retention times and MS2 fragment spectra of these ions matched those produced in our *in vitro* reactions containing recombinant SidL, confirming their identities as 2-AMP-3PG and 2-AMP-glycerate (Figs. S6A-B). The presence of 2-AMP-glycerate in these cells is likely due to the dephosphorylation of 2-AMP-3PG by a cellular phosphatase, as SidL did not adenylate glycerate *in vitro* (Fig. S5B). Together, these data indicate that SidL adenylates 3PG within cells.

3PG is produced as an intermediate of glycolysis, the central metabolic pathway that converts glucose into pyruvate (Fig. 5C).^69^ During glycolysis, phosphoglycerate mutase phosphorylates 3PG at its 2-position hydroxyl group and then dephosphorylates the 3-position to produce 2PG (EC5.4.2.11).^70^ Alternatively, 3PG can be used for *de novo* serine synthesis whereby phosphoglycerate dehydrogenase oxidizes the 2-position hydroxyl group to produce 3-phosphohydroxypyruvate (EC1.1.1.95).^70^ Thus, adding AMP at the 2-position of 3PG would make it incompatible with these two canonical metabolic reactions (Fig. S7A), prompting us to ask if SidL expression inhibits glycolysis. Therefore, we measured the amount of glucose and lactate in the media of HEK293T cells expressing GFP or SidL over time to calculate the rates of glucose consumption and lactate production as proxies for glycolytic activity. From these measurements, we found that SidL reduced both of these rates relative to GFP-expressing cells (Figs. 5D-E), suggesting that SidL hinders overall glycolytic activity.

To gain more direct insight into how SidL affects glycolysis and broader metabolism, we analyzed the steady-state abundances of 140 metabolites from HEK293T cell lysates using an untargeted metabolomics approach (Table S5). In our analysis, we detected all the glycolytic intermediates except 1,3-BPG which is typically undetectable due to its rapid consumption in cells and chemical instability.^71^ The isobaric 3PG and 2PG (3PG/2PG) were amongst the most strongly decreased metabolites by SidL expression (Figs. 5F and S7B). Moreover, we found that the downstream glycolysis intermediates, phosphoenolpyruvate (PEP) and pyruvate, as well as lactate were also reduced in the presence of SidL (Figs. 5F and S7B), as were the levels of both phosphoserine (pSer) and serine (Figs. 5F and S7B). Interestingly, the levels of intermediates upstream of 3PG, fructose 6-phosphate (F6P), fructose 1,6-bisphosphate (FBP), dihydroxyacetone phosphate (DHAP), and glyceraldehyde 3-phopshate (G3P), increased in response to SidL while glucose and glucose 6-phosphate (G6P) were unchanged (Figs. 5F and S7B). Finally, we saw that SidL induced a global loss of intermediates from other metabolic pathways including those of the TCA cycle (Fig. S7B), as would be expected from broad disruption of glycolysis under our experimental conditions. In addition to its effects on glycolytic intermediates, SidL also impacted the abundance of adenosine nucleotides with an increase in AMP and a reduction in ATP and ADP (Fig. S7B); these observations are consistent with the ability of SidL to hydrolyze ATP into AMP *in vitro* (Fig. 3B),^34^ and may explain why SidL activates AMPK in HEK293T cells (Fig. 3F).

While these results were striking, mTORC1 activity is known to affect metabolic processes, including glycolysis.^50,72,73^ Indeed, Torin 1 treatment reduced glycolytic activity in HEK293T cells, as determined by its effects on glucose consumption and lactate production rates (Figs. 5D-E). Because SidL inhibited mTORC1 (Fig. 1D), we wanted to verify that its observed effects on glycolysis were not just a consequence of mTORC1 inhibition. Thus, we also analyzed changes in metabolite abundances in Torin 1 treated cells. We found that unlike SidL, which only decreased 3PG/2PG and downstream glycolytic intermediates (Figs. 5F and S7B), Torin 1 inhibition of mTORC1 caused all glycolytic intermediates, except glucose, to decrease (Figs. 5G and S7C), with a more generalized, broader decrease in other metabolites including those of the TCA cycle (Fig. S7C). Moreover, mTORC1 inhibition did not strongly impact serine biosynthesis as pSer and serine levels did not significantly change in response to Torin 1 (Figs. 5G and S7C). Furthermore, direct comparison of metabolites from SidL and Torin 1-treated cells minimized the relative differences for most metabolites (Fig. S7D), highlighting the very specific changes in glycolytic and serine biosynthesis intermediates by SidL. Collectively, these data suggest that the effects of SidL on glycolysis are not simply a consequence of mTORC1 inhibition but rather indicate that SidL adenylation of 3PG in cells reduces 3PG availability for downstream metabolic processes.

### SidL adenylates 3PG during *L. pneumophila* infection.

Having established that SidL adenylates 3PG *in vitro* and when exogenously expressed in HEK293T cells, we next investigated whether SidL does so during *L. pneumophila* infection. To explore this, we individually infected THP-1 cells (human monocyte-derived macrophages) with wild-type *L. pneumophila*, a Δ*sidL* deletion mutant, or a Dot/Icm mutant (*dot-*) that lacks a functional T4SS and cannot translocate effectors into the host cell^74^ and compared them to similarly treated uninfected cells. At 1, 3, 6 and 9 hours post infection (hpi), cells were harvested and extracted metabolites were analyzed by LC-MS using a targeted multiple reaction monitoring method to measure the abundance of 2-AMP-3PG and 2-AMP-glycerate.

**Figure 6.**
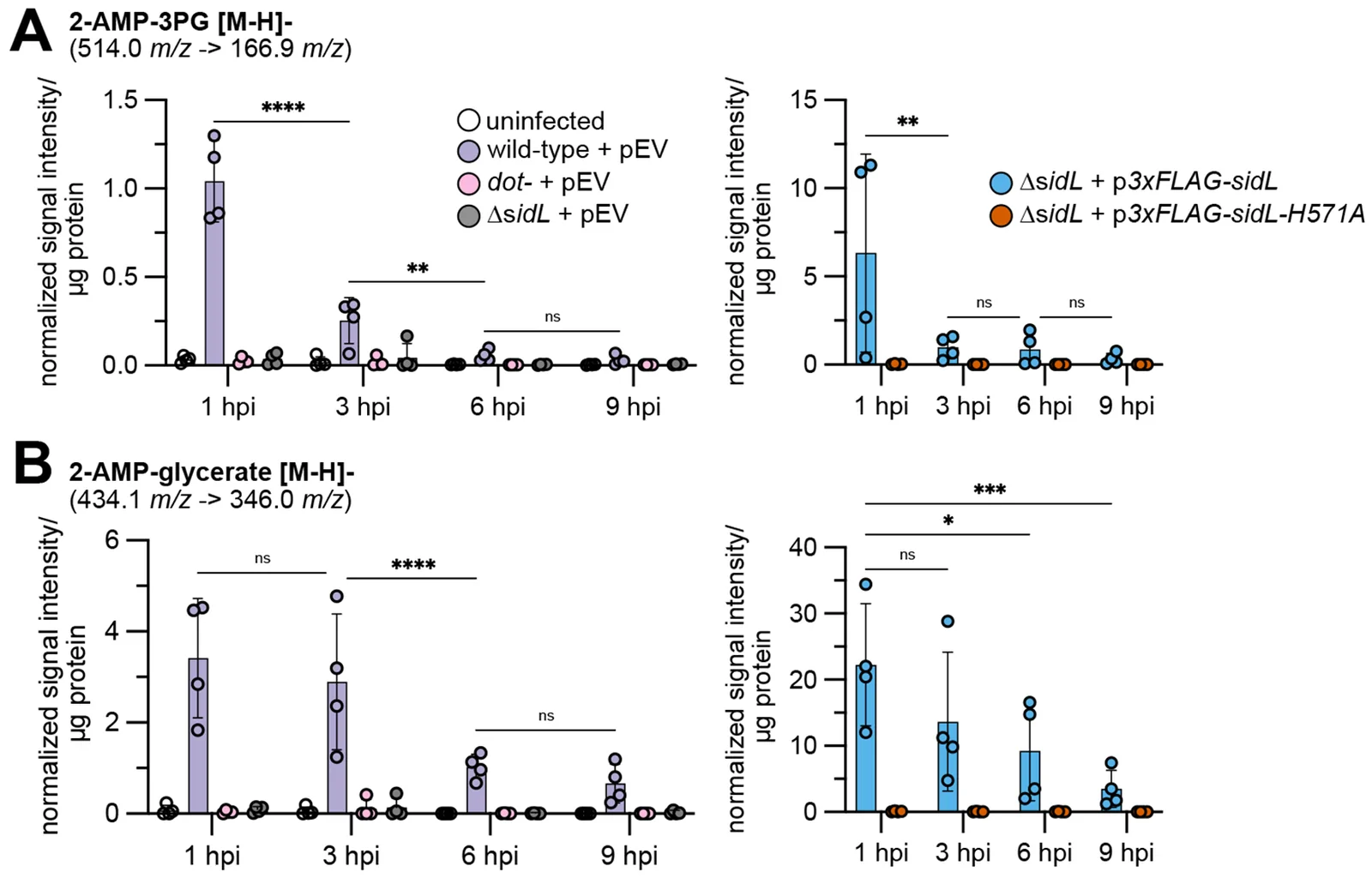
*L. pneumophila* adenylates 3-phosphoglycerate in a SidL-dependent manner. **(A, B)** LC-MS detection of 2-AMP-3PG **(A)** and 2-AMP-glycerate **(B)** in either uninfected THP-1 cells or cells infected with the indicated *L. pneumophila* strains at the indicated hours post infection (hpi) (see STAR Methods). The quantified *m/z* transition fragments for each molecule are indicated, and signals are normalized to a heavy AMP standard in the extraction buffer and total cellular protein content. Bars are mean ± SD with points representing biological replicates (n=4). *m/z* of each metabolite is indicated. Two-way ANOVA with multiple comparisons: not significant (ns), *p* ≤ 0.05 (*), *p* ≤ 0.01 (**), *p* ≤ 0.001 (***), *p* ≤ 0.001 (****). See Fig. S8 and Table S6.

We detected 2-AMP-3PG in THP-1 cells infected with wild-type bacteria but not in uninfected cells, indicating that 3PG is adenylated during infection (Fig. 6A). The presence of 2-AMP-3PG was effector-dependent as it was not found in cells infected with the *dot-* bacteria. Most importantly, 2-AMP-3PG was not detected in cells infected with Δ*sidL* mutant bacteria, and this loss could be rescued by reintroducing a copy of wild type *sidL* on a self-replicating plasmid, but not a catalytically inactive variant (*sidL-H571A*) (Fig. 6A). The inability of *sidL-H571A* to restore 2-AMP-3PG production was not due to protein instability, as immunoblotting of *L. pneumophila* lysates showed similar levels of SidL-H571A and SidL produced in their bacterial host (Fig. S8A). These results demonstrate that SidL is solely responsible for 2-AMP-3PG production during *L. pneumophila* infection of macrophages (Fig. 6A). Similarly, 2-AMP-glycerate was only detected in THP-1 cells infected with bacterial strains harboring intact *sidL* (Fig. 6B). Notably, 2-AMP-3PG and 2-AMP-glycerate were more abundant at 1 and 3 hpi than at 6 and 9 hpi in cells infected with wild-type bacteria suggesting temporal regulation of their synthesis over the infection cycle (Fig. 6).

We also analyzed the isobaric 3PG/2PG and other glycolytic intermediates in these samples to ask if *L. pneumophila* infection impacts the abundances of these metabolites in THP-1 cells (Figs. S8B-I). Notably, we observed decreases for 3PG/2PG upon infection with wild-type bacteria but not with *dotA-*bacteria, indicating effector-driven effects (Fig. S8F). However, these effects were not due to SidL as 3PG/2PG was also decreased in cells infected with Δ*sidL* mutant bacteria. Moreover, we observed Dot-dependent but SidL-independent decreases in PEP at some time points while other metabolites showed more modest decreases or remained unchanged (Figs. S8B-I). Finally, we analyzed adenosine nucleotides during infection to ask if SidL impacts host ATP, ADP, and AMP levels as previously proposed.^34^ Notably, AMP levels did not increase during infection irrespective of the presence of SidL (Fig. S9A). Moreover, we found that ATP and ADP decreased in cells infected with wild-type bacteria but not *dot-* bacteria (Figs. S9B-C), again however independent of SidL.

Taken together, these data strongly indicate that *L. pneumophila* synthesizes 2-AMP-3PG during infection downstream of SidL expression but suggests that broader impacts on host cell carbon metabolism are the result of the combined impact of many independent effectors in a complex infection environment.

## Discussion

Toxins and effectors exhibit extraordinary diversity with many targeting universally conserved cellular features, including information-transmitting molecules like ribosomes and tRNAs, energy currencies like NTPs and NAD+, and cellular membranes and lipids.^1^ While we initially sought to understand how SidL inhibits translation, we unexpectedly discovered that SidL adenylates the glycolytic intermediate 3PG *in vitro* (Figs. 4C-G), in HEK293T cells (Fig. 5A), and during *L. pneumophila* infection of macrophages (Fig. 6A) to produce the metabolite 2-AMP-3PG (Fig. 4H). Together, these findings reveal that metabolites from host glycolysis, one of the most conserved and critical metabolic pathways, are directly modified by pathogen effectors. Current chemical databases lack 2-AMP-3PG, suggesting that it is a previously unknown metabolite. The placement of AMP at the 2-position of 3PG would be expected to make it incompatible with eukaryotic metabolic reactions that use 3PG (Fig. S7A). Consistent with this idea, we observed decreases not only in the steady-state levels of the isobaric 3PG and 2PG but also of the glycolytic and serine biosynthesis intermediates downstream of 3PG in response to SidL expression in HEK293T cells (Figs. 5F and S7B). Thus, we propose that unchecked 3PG adenylation by SidL effectively starves these cells of 3PG. Multiple studies have shown that mTORC1 is inhibited by disruptions to glycolysis.^36-44^ Therefore, this metabolic perturbation by SidL constitutes a reasonable explanation for how SidL inhibits mTORC1 signaling (Fig. 1D) and consequently translation (Fig. 5C) under these conditions.

How mTORC1 senses glycolytic perturbations is an area of active study.^36-44^ It was proposed that mTORC1 responds to low levels of DHAP and G6P.^36,44^ However, our data indicate that neither DHAP nor G6P are depleted in SidL-expressing cells (Fig. 5F), arguing that mTORC1 sensing of these metabolites is not involved under these conditions. Since the levels of glycolysis intermediates are sensitive to multiple feedback mechanisms that control glycolysis itself and other metabolic pathways,^75^ multiple mechanisms may exist to regulate mTORC1 activity in response to glycolytic disruptions. One possibility is that the level of 3PG itself or one of the other depleted glycolysis intermediates (Fig. 5F) is sensed by mTORC1 in some contexts. Thus, SidL may be a useful reagent to study the crosstalk between mTORC1 and glycolysis.

While our data identify 3PG adenylation as the preferred activity of SidL (Figs. 4D and S4B), we also detected basal ATP hydrolysis activity in the absence of 3PG (Figs. 3B-C and 4D), consistent with a recent study from the Luo group.^34^ This latter activity readily explains SidL-mediated inhibition of ATP-dependent processes *in vitro* (Figs. 3D-E)^34^ and the metabolomic analysis indicating that SidL decreases the level of ATP and increases the level of AMP when ectopically expressed in HEK293T cells (Fig. S7B). As SidL generates AMP in the absence of 3PG *in vitro* (Figs. 4D and S4B), we anticipate that in cells where physiological concentrations of ATP (1-5 mM)^76-80^ greatly exceed that of 3PG (50-80 μM),^79-82^ there may be SidL-dependent uncoupled ATP hydrolysis there as well. However, we cannot uncouple the ATP hydrolysis and adenylyltransferase activities of SidL and therefore we cannot determine if the corresponding loss in ATP is sufficient to inhibit translation in cells as it does *in vitro* (Figs. 3D-E). Nevertheless, we did not see SidL-dependent ATP depletion or AMP production during *L. pneumophila* infection (Fig. S9), possibly because expression of SidL there is considerably lower. We did however see SidL-dependent production of 2-AMP-3PG and its derivative 2-AMP-glycerate during infection (Fig. 6) lending strong support to the hypothesis that 3PG adenylation its true activity. Collectively, our results indicate that SidL should not be grouped with the effectors that directly inhibit translation.^14-24^ Rather, SidL should be viewed as a metabolite-targeting effector, a lens that raises multiple questions regarding its physiological role.

Beyond the deleterious effects that SidL has on cells when expressed alone at high levels in HEK293T cells, we reason that *L. pneumophila* uses SidL in a more subtle manner during infection since the bacterium requires a live host cell to replicate.^83^ Thus, *L. pneumophila* must strike a balance between keeping its host alive and the reactions catalyzed by its effectors. Notably, while we saw clear SidL-dependent generation of 2-AMP-3PG and 2-AMP-glycerate production during infection (Fig. 6), the levels of most glycolytic intermediates only changed modestly or remained relatively constant with only PEP and 3PG/2PG significantly decreasing in an infection-dependent manner at some time points (Fig. S8B-I). Importantly, these changes were not SidL-dependent but were effector-dependent, hinting at the existence of additional effectors that modulate glycolytic metabolites, possibly including 3PG and/or 2PG. This is not surprising as there are other effectors that target central carbon metabolism in the host. Two of these known effectors decrease mitochondrial function with MitF inducing mitochondrial fragmentation to impair oxidative phosphorylation^30^ and Ceg3 inactivating mitochondrial ADP/ATP translocases.^25,26^ In addition, *L. pneumophila* encodes the effector LamA that degrades host glycogen stores to directly increase the availability of G1P and G6P.^31^ Collectively, such modulation of central carbon metabolism is thought to generally increase host glycolytic activity.^30,31^ However, our discovery that SidL adenylates 3PG suggests that *L. pneumophila* has other unappreciated effects on specific glycolytic intermediates, perhaps to exert finer control over host glycolysis, and opens an avenue for future identification of other effectors that target host glycolysis and metabolism more broadly.

Beyond identifying 2-AMP-3PG and 2-AMP-glycerate being made in a SidL-dependent manner during infection, we saw that their abundances were highest at 1 and 3 hpi compared to 6 and 9 hpi (Fig. 6). These data suggest that SidL is most active at early stages of the infection cycle and is temporally regulated. It was recently shown that the effector AnkJ/LegA11 directly binds SidL and inhibits its ATP hydrolysis activity.^34,84^ Such so-called meta effectors are emerging as common mechanisms by which effectors are regulated in *L. pneumophila* and elsewhere.^85^ As such, we speculate that heightened SidL activity at 1 and 3 hpi indicates the importance of targeting 3PG early during infection and the temporal decrease of 2-AMP-3PG and 2-AMP-glycerate could suggest the existence of an effector that metabolizes these molecules.

Our study also raises questions about the roles of 2-AMP-3PG or 2-AMP-glycerate during infection. Previous studies suggest that *L. pneumophila* uses carbon derived from host glucose and serine for its own intracellular metabolism.^86-89^ Because 3PG is at the branchpoint between glycolysis and serine biosynthesis (Fig. 5C),^90^ it is tempting to speculate that *L. pneumophila* targets 3PG for nutrient scavenging and that 2-AMP-3PG and 2-AMP-glycerate store 3PG for later use. Alternatively, SidL may adenylate 3PG for purposes outside of manipulating metabolism. One possibility is that 2-AMP-3PG or 2-AMP-glycerate serve as cofactors or regulatory ligands for another effector akin to how the host-specific metabolite inositol hexakisphosphate activates the effector Lpg2603.^91^ Alternatively, these molecules could act as host enzyme inhibitors, like the galactose mimetic synthesized by *Pseudomonas syringae* that inhibits host β-galactosidase,^92^ or they could interfere with second messenger signaling, similar to the effects of pathogens manipulating host cyclic AMP levels.^93^ Thus, further study of 2-AMP-3PG and 2-AMP-glycerate is needed to understand their role during infection.

Finally, beyond the activity of SidL, our study identifies the MCF1-SHE domain as one of several that catalyze adenylation.^94^ We found extensive sequence diversity amongst MCF1-SHE domains (Figs. 2G-H), and despite being relatively closely related, SidL and LnaB evolved different substrates: a metabolite for SidL and proteins for LnaB.^11,12^ Moreover, we found that LegK4 and Lpg0209, the two other MCF1-SHE domain proteins from *L. pneumophila*, do not appear to broadly adenylate proteins, at least under the conditions tested (Fig. 2I). Similarly, certain LnaB homologs from other *Legionella* species do not adenylate proteins *in vitro*.^12^ Together, these observations are consistent with the broader substrate and enzymatic diversity of the PAP2 superfamily to which the MCF1-SHE domain is related (Figs. 2G-H).^56-64^ Thus, we suggest that yet uncharacterized MCF1-SHE proteins may catalyze analogous nucleotidyl or phosphoryl transfer reactions on a variety of substrates.

Together, our data support a model in which SidL adenylates the glycolytic intermediate 3PG to produce 2-AMP-3PG. We anticipate that chemical modification of soluble metabolites by pathogen effectors may be a broadly used mechanism through which pathogens interface with their host cell.

### Limitations of the study

While we saw clear impacts on glycolysis when SidL is ectopically expressed in HEK293T cells (Figs. 5F and S7B), we did not see evidence for SidL-dependent effects on glycolysis during infection (Fig. S8B-I) despite strong evidence for SidL being responsible for 2-AMP-3PG production in both scenarios (Figs. 5A and 6A). This discrepancy could be due to the relatively lower levels of expression in an infection model or to the presence of additional effectors that target host glycolytic intermediates and mask SidL-specific effects. Indeed, we saw infection-dependent changes in the levels of PEP and 3PG/2PG that relied on *L. pneumophila* having a functional T4SS (Fig. S8F-G). It is well understood that *L. pneumophila* often encodes multiple, redundant effectors that target the same or related host pathways.^13^ In line with this thinking, Δ*sidL* mutant *L. pneumophila* strains do not show impaired replication during infection.^16,34,84^ Therefore, the identification of other effectors that directly target host glycolysis may be needed to understand how *L. pneumophila* benefits from employing SidL.

## STAR Methods

### EXPERIMENTAL MODEL AND STUDY PARTICIPANT DETAILS

HEK293T cells were obtained from the American Type Culture Collection (ATCC #CRL-3216). The PRKAA1/PRKAA2 double knockout line (AMPKα1/2 DKO) and its parental HEK293T line were obtained from David Sabatini.^44^ HEK293T cells were thawed and passaged at least twice before their use in experiments. Unless otherwise stated, HEK293T cells were cultured in Dulbecco’s Modified Eagle Medium (DMEM; Gibco #11995065; 4.5 g/L D-glucose, 584 mg/L L-glutamine, 110 mg/L sodium pyruvate) supplemented with 10% fetal bovine serum (FBS; Gibco #26140079). HEK293T cells were cultured at 37°C in a 5% CO_2_ humidified incubator and passaged every 2-3 days and routinely assayed for mycoplasma contamination (Applied Biological Materials #G238).

THP-1 cells (ATCC #TIB-202) were cultured in Advanced RPMI 1640 medium (Gibco #12633012) supplemented with 10% heat-inactivated FBS and 2 mM L-glutamine (Gibco #25030081) at 37°C in a 5% CO2 humidified incubator.

All *Legionella* strains were generated in the *L. pneumophila* Philadelphia-1 strain Lp02.^74^ *L. pneumophila* strains were cultured at 37°C in liquid N-(2-acetamido)-2-aminoethanesulfonic acid (ACES)–buffered yeast extract (AYE) medium or on solid charcoal ACES-buffered yeast extract (CYE) medium^95^ containing L-cysteine (0.4 mg/ ml; Sigma-Aldrich # C7352), ferric nitrate (0.135 mg/ml; Sigma-Aldrich #216828) and, when appropriate, thymidine (0.1 mg/ml; Sigma-Aldrich # T1895) (CYET).

Cells, reagents, software, and equipment used in this study are listed in KEY RESOURCES TABLE.

### METHOD DETAILS

#### Plasmid cloning

Plasmids used in this study are listed in KEY RESOURCES TABLE and fully annotated plasmid maps (as GenBank sequence files) are available in Supplemental Data S3. All plasmids generated in this study were confirmed by whole-plasmid sequencing (Plasmidsaurus) and are available upon request.

For mammalian expression plasmids, *Homo sapiens* codon-optimized *Legionella pneumophila* effector sequences were generated using the Codon Optimization Tool (Integrated DNA Technologies) accessed May 2023 (SidL and SidL-S527A) and June 2024 (LnaB, LegK4(Δ1-58), and Lpg0209); gene blocks containing these sequences were synthesized by Twist Biosciences and subcloned into PCR-linearized pJB63 (pcDNA3.1-P_CMV_::3xFLAG-GFP) by Gibson Assembly (New England Biolabs #E2611) to replace the GFP coding sequence and generate N-terminal 3xFLAG-tagged fusions. Aside from SidL-S527A, mutations and truncations of SidL were introduced by inverse PCR.

For *E. coli* expression plasmids, 3xFLAG-GFP was PCR amplified from pJB63 and effector sequences (*L. pneumophila* codon usage sequences) were PCR amplified from plasmids encoding N-terminal 3xFLAG-tagged effectors and sub-cloned into PCR-linearized pGEX-GST-3C using Gibson Assembly to clone pJB193 (pGEX-GST-3C-3xFLAG-GFP), pJB194 (pGEX-GST-3C-3xFLAG-SidL), pJB209 (pGEX-GST-3C-3xFLAG-SidL-H571A), and pJB213 (pGEX-GST-3C-3xFLAG-LnaB).

For *L. pneumophila* expression plasmids, the *L. pneumophila* codon usage sequences for *sidL* and *sidL-H571A* were PCR amplified using forward primer JBO372 (ggcggatccCAAAACTTAGATGAGATTCTAAAGAAACTGAG) and reverse primer JBO373 (gcctctagaCTAGCACCCATAAACAGTTCCATC) from pJB194 and pJB209 containing *Bam*HI and *Xba*I restriction sites (underlined sequences), respectively, and sub-cloned into pDTI116^96^ cut with the same enzymes to generate pJB245 and pJB246, respectively.

The *Escherichia coli* strain DH5α (Invitrogen #18265017) was used to maintain and propagate plasmids, unless otherwise stated. For bacterial transformation, plasmids were purified using GeneJET Plasmid Miniprep Kit (ThermoFisher Scientific #K0503). For HEK293T transfection, plasmids were purified from DH5α using ZymoPURE II Plasmid Midiprep Kit (Zymo Research #D4201).

#### Construction of *L. pneumophila* Δ*sidL* mutant

*sidL* was deleted in *L. pneumophila* Philadelphia-1 strain Lp02 using a double recombination strategy^97^ employing the suicide vector pSR47s containing ~750 bp upstream and downstream of the *sidL* gene (pJB251) to generate Lp02 Δ*sidL* strain (TO4654).

pJB251 was generated as follows. The sequence upstream of the *sidL* gene was PCR amplified using forward primer JBO381 (gccgagctcCCAAAGCGATTACCTTCAGCATC) and reverse primer JBO382 (gccggatccATAACCCTCTACCTCTTAGCATTGTC) which contains *Sac*I and *Bam*HI restriction sites (underlined sequences), respectively. The sequence downstream of the *sidL* gene was PCR amplified using forward primer JBO383 (gccggatccAGGATAATTTGGGTTCCCATTCCC) and reverse primer JBO384 (gcctctagaGCATCAGATGGTATAACAACTTTGG) which contains *Bam*HI and *Xba*I restriction sites (underlined sequences), respectively. Each fragment was digested with the appropriate restriction enzyme pair then subcloned into pSR47s^97^ digested with *Sac*I and *Xba*I and propagated in DH5α λpir.^98^

#### Plasmid DNA transfections

HEK293T cells were seeded as indicated in the below experimental sections and the indicated amounts of plasmid DNA (pDNA) were transfected using Lipofectamine 3000 (Invitrogen #L3000015). For transfection, pDNA was diluted in Opti-MEM media (Gibco #51985034) and mixed with P3000 reagent (2 μL/μg pDNA) to make Solution A and Lipofectamine 3000 (2 μL/μg pDNA) was diluted into an equivalent volume of Opti-MEM to make Solution B. Solutions A and B were mixed and incubated for 10-15 minutes at room temperature then applied to cells drop-wise. Plasmids used in each experiment are indicated in the corresponding figure legends.

#### Cell proliferation assays

5x10^5^ HEK293T cells were seeded in a 6-well dish in 2 mL media. Cells were transfected ~24 hours after seeding with 1.5 μg of pDNA, as described in “Plasmid DNA transfections”, then imaged every 3 hours over a 48-hour period using a CELLCYTE X imager (Cytena). Cell confluency was calculated using CELLCYTE Studio (Cytena, settings: contrast sensitivity 50 a.u., smoothing 2 a.u., filled hole size 100 μm^2^, minimum object size 100 μm^2^). Data were plotted using GraphPad Prism using an ordinary one-way ANOVA with Dunnet’s multiple comparisons for Fig. 2F.

#### Puromycin incorporation assays

5x10^5^ HEK293T cells were seeded in a 6-well dish in 2 mL media. Cells were transfected ~28-30 hours after seeding with 1 μg of pDNA, as described in “Plasmid DNA transfections”. After overnight incubation (~16-18 hours), media was exchanged with fresh media. After 2 hours of incubation, 18 μM puromycin (Sigma-Aldrich #P7255) was added and incubated for 5 minutes at 37°C. For emetine pretreatment, 360 μM emetine was added to cells for 20 minutes prior to puromycin treatment. Cells were then quickly harvested by washing with 1 mL 1X PBS pH 7.4 (Gibco #10010023) containing 360 μM emetine (Millipore #324693) then lysed in 200 μL ice-cold RIPA buffer (ThermoFisher Scientific #89901) supplemented with 360 μM emetine, 1X Halt Protease and Phosphatase Inhibitor Cocktail (ThermoFisher Scientific #71636), 10 mM Na_2_HPO_4_ (Sigma #71636), 10 mM β-glycerophosphate (Sigma #G9422), 1 mM TCEP (Gold Biotechnology #TCEP2), and 42.5 units/mL benzonase (Millipore #E1014)). Cells were scraped, gently resuspended using a pipette, and transferred to a pre-chilled tube on ice. Lysates were clarified by centrifugation at 14,000 xg for 5 minutes at 4°C then transferred to a fresh pre-chilled tube on ice and used immediately or flash frozen in liquid nitrogen before being stored at −80°C. Immunoblotting was done as described in “Immunoblotting”.

#### Sucrose gradients and polysome profiling

2x10^6^ HEK293T cells were seeded in a 10-cm dish in 10 mL media. Cells were transfected ~28-30 hours after seeding with 6 μg of pDNA, as described in “Plasmid DNA transfections”. After overnight incubation (~16-18 hours), media was exchanged with fresh media. After 2 hours of incubation, cells were washed with 8 mL 1X PBS pH 7.4 lysed in 300 μL ice-cold gradient lysis buffer (50 mM HEPES-KOH pH 7.4, 100 mM KOAc, 15 mM Mg(OAc)_2_, 5% glycerol, 0.25% NP-40 alternative (Millipore #492018), 1 mM TCEP, 1X Halt Protease and Phosphatase Inhibitor Cocktail, and 20 units/mL Turbo DNAse (Invitrogen #AM22390)). Cells were scraped, gently resuspended using a pipette, and transferred to a pre-chilled tube on ice. Lysates were clarified by centrifugation at 8,000 xg for 5 minutes at 4°C then transferred to a fresh pre-chilled tube on ice and used immediately or flash frozen in liquid nitrogen before being stored at −80°C.

RNA concentration of the lysates was quantified using Qubit RNA High Sensitivity Assay (ThermoFisher Scientific #Q32855) and normalized to the lowest RNA concentration in 300 μL final volume in the gradient lysis buffer. 250 μL of normalized lysates (22-40 μg total RNA) were layered onto the top of pre-chilled 10-50% sucrose gradients containing 25 mM HEPES-KOH pH 7.4, 100 mM KOAc, 5 mM Mg(OAc)_2_, and 1 mM TCEP made in 14x89 mm tubes (Seton Scientific #7030) and ultracentrifuged in a SW-41 swinging-bucket rotor (Beckman Coulter #331362) at 40,000 rpm for 1 hour and 45 minutes at 4°C. Gradient traces were recorded with continuous UV absorbance (260 nm) using a Biocomp Piston Gradient Fractionator and visualized in RStudio using ggplot2.^99^

For experiments with Torin 1 treatment, 0.03% DMSO (Invitrogen #D12345) or 300 nM Torin 1 (Selleckchem #S2827) was added 2 hours post-transfection and their inclusion was sustained during media exchange.

For the G-25 column experiment, samples were processed as above except four replicate dishes of cells were cultured for each condition. The replicate cells were lysed and pooled together to ensure ample material. A PD MiniTrap desalting column with Sephadex G-25 resin (Cytiva #28918007) was pre-equilibrated in the gradient lysis buffer. The pooled lysates were split in half (500 μL each) and one half was passed through the column by gravity. After elution from the column, RNA concentration was quantified for each sample using Qubit RNA High Sensitivity Assay and 26 μg total RNA was subjected to ultracentrifugation as described above.

#### Cell culture for immunoblotting

4-5x10^5^ HEK293T cells per well were seeded in a 6-well dish in 2 mL media. Cells were transfected ~28-30 hours after seeding with 1 μg of pDNA, as described in “Plasmid DNA transfections”. After overnight incubation (~16-18 hours), media was exchanged with fresh media. After 2 hours of incubation, cells were quickly harvested by washing with 1mL 1X PBS pH 7.4 then lysed in 150 μL ice-cold lysis buffer (RIPA buffer supplemented with 1X Halt Protease and Phosphatase Inhibitor Cocktail, 10 mM Na_2_HPO_4_, 10 mM β-glycerophosphate, 1 mM TCEP, and 42.5 units/mL benzonase). Cells were scraped, gently resuspended using a pipette, and transferred to a pre-chilled tube on ice. Lysates were clarified by centrifugation at 14,000 xg for 5 minutes at 4°C then transferred to a fresh pre-chilled tube on ice and used immediately or flash frozen in liquid nitrogen before being stored at −80°C. Immunoblots were done as described in “Immunoblotting”.

For experiments with mTOR inhibitors, 0.15% DMSO, 300 nM Torin 1, or 100 nM rapamycin (Selleckchem #S1039) was added 2 hours post-transfection and their inclusion was sustained during media exchange.

For experiments comparing wild-type and AMPK-DKO HEK293T cells,^44^ wild-type cells were seeded at 4x10^5^ cells per well and AMPK-DKO cells were seeded at 5x10^5^ cells per well to account for growth differences.

#### Sequence analysis

Sequence homology searches using SidL and its closest orthologs as queries identified its immediate relationship to previously unannotated homologs (NM1S families) and further iterative database searching established homology with the classical MCF1-SHE domain. Iterative sequence profile searches were conducted using the PSI-BLAST (RRID:SCR_001010)^100^ and JACKHMMER (RRID:SCR_005305)^101^ programs with a profile-inclusion threshold of expect (e)-value at 0.005 against the non-redundant database of the National Center for Biotechnology Information (NCBI) clustered down to 50% (nr50) using the MMseqs2 program (RRID:SCR_022962)^102^. Remote homology searches, used to establish relationship between the MCF1-SHE and PAP2-Haloperoxidase domains, were performed using profile-profile comparisons with the HHpred program (RRID:SCR_010276)^103^ against profile libraries comprised of the PFAM (RRID:SCR_004726)^104^ and PDB (RRID:SCR_012820)^105^ databases. A sequence similarity network was constructed using the HHalign program (RRID:SCR_016133)^106^ to perform profile-profile comparisons, with the resulting e-value scores serving as edges. This network was then analyzed using the Leiden community-finding algorithm^107^ to detect sub-networks. Network analysis and visualization were performed using the R igraph library.^108^ See Table S1 for domain annotations and HHalign scores.

#### Comparative genomics and domain identification

Genomic neighborhoods were obtained from the NCBI Genome database (RRID:SCR_002474).^109^ Conservation analysis of these neighborhoods was performed by clustering the protein products of neighboring genes with the MMseqs2 program.^102^ Domain identification was conducted with HMMs from the Pfam database^104^ utilizing the RPSBLAST^110^ and HMMSCAN (RRID:SCR_005305)^111^ programs.

#### Phylogenetic analysis

Multiple sequence alignments (MSAs) for phylogenetic analysis were constructed using the MAFFT program (RRID:SCR_011811)^112^ with the local-pair algorithm, together with the following non-default parameters: maximum iterations=3000, gap opening penalty=1.5, and offset penalty=0.2. MSA refinements were guided by DALIlite structural alignments (RRID:SCR_013433).^113^ The input MSA for phylogenetic analysis contained 925 domains from 865 proteins from across the distinct identified families, with individual family representation in the tree proportional to the total number sequences retrieved from the nr50 database (see above). Sequences were randomly selected for inclusion using the sample function in R. Phylogenetic analysis was conducted using the maximum likelihood method implemented by the FastTree2 program (RRID:SCR_015501).^114^ Local support values to estimate reliability of splits in trees are calculated using Shimodaira-Hasegawa test-like algorithm as described.^114,115^ Trees were rendered using the TreeViewer program.^116^

#### Structural analyses and visualization

SidL, SidL-actin, LnaB-actin, and classical MCF1-SHE predictions were done using AlphaFold3 webserver^53^ with default settings; structures were generated May 2025 (SidL and SidL•actin), June 2025 (classical MCF1-SHE), and July 2025 (LnaB-actin). Structures were visualized using UCSF ChimeraX (version 1.4)^117^ and MOL* (RRID:SCR_017551).^118^ Publicly available experimental structures used in this study were obtained from RCSB Protein Data Bank (LnaB-actin complex, PDB 8J9B; “classical PAP2”, PDB 5JKI; “PAP2-diffusible”, PDB 7F17; “haloperoxidase” PDB 4USZ). Structure similarity searches were performed using the DaliLite^113^ and FOLDSEEK (RRID:SCR_027018)^119^ programs.

#### Affinity purification of 3xFLAG-tagged proteins

2x10^6^ HEK293T cells were seeded in a 10-cm dish in 10 mL media. Cells were transfected ~28-30 hours after seeding with 6 μg of pDNA, as described in “Plasmid DNA transfections”. After overnight incubation (~16-18 hours), cells were washed with 8 mL 1X PBS pH 7.4 then lysed in 700 μL ice-cold affinity purification (AP) lysis buffer (50 mM HEPES-KOH pH 7.4, 100 mM KOAc, 15 mM Mg(OAc)_2_, 5% glycerol, 0.25% NP-40 alternative, 1X Halt Protease and Phosphatase Inhibitor Cocktail, and 20 units/mL Turbo DNAse). Cells were scraped, gently resuspended using a pipette, and transferred to a pre-chilled tube. All subsequent steps were conducted on ice or at 4°C, unless otherwise noted. Lysates were clarified by centrifugation at 8,000 xg for 10 minutes then transferred to a fresh pre-chilled tube on ice. 50 μL of each lysate was taken aside as inputs and mixed with 10 μL of 6X Laemmli buffer then heated for 5 minutes at 95°C before −80°C storage.

ANTI-FLAG M2 magnetic beads (Millipore #M8823; 12.5 μL of the 50% slurry per sample) were washed thrice with AP lysis buffer. The clarified lysates were added to pre-washed beads and incubated for 1-1.5 hour with gentle nutation. Following, the beads were washed thrice in 500 μL AP wash buffer 1 (AP lysis buffer but with 0.1% NP-40 alternative and no DNAse) then washed thrice with 500 μL AP wash buffer 2 (wash buffer 1 but with 0.05% NP-40 alternative and no glycerol). The beads were then resuspended in 50 μL of AP elution buffer (wash buffer 2 supplemented with 400 μg/mL 3xFLAG peptide (Sigma-Aldrich #F4799)) and incubated for 1 hour with gentle nutation. Afterwards, the eluates were transferred to fresh tubes and either flash frozen in liquid nitrogen (for mass spectrometry) or supplemented with Laemmli buffer to 1X (for immunoblotting) before −80°C storage.

For mass spectrometry, affinity purifications were done in technical triplicate (n=3), each from a separate dish of transfected cells and processed as described in the “Mass spectrometry of affinity-purifications” subsection.

#### Mass spectrometry of affinity-purifications

##### Sample preparation.

Affinity purification eluates (see above) were buffer exchanged using SP3 paramagnetic beads (GE Healthcare).^120^ Briefly, samples were resolubilized in 10 mM TEAB and disulfide bonds reduced with DTT (5 mM final concentration) for 1 hour at 60°C. Samples were cooled to room temperature and pH adjusted to ~8.0, followed by alkylation with iodoacetamide (10 mM final concentration) in the dark at room temperature for 15 minutes. Next, 100 μg SP3 beads were added to the samples and 100% ethanol was added to achieve a final ethanol concentration of 50% v/v. Samples were incubated at room temperature with shaking for 5 minutes. Following protein binding, beads were washed with 180 μL 80% ethanol three times. Proteins were digested on-bead with 1 μg trypsin/LysC protease mix (Pierce #A41007) at 37°C overnight. Resulting peptides were separated from the beads using a magnetic tube holder. Supernatants containing peptides were removed from the beads and dried using vacuum centrifugation, resuspended in 100 μL 0.1% trifluoroacetic acid, and desalted on OASIS HLB μElution plates (Waters Corp #186001828BA) per manufacturer’s instructions. Eluates were dried using vacuum centrifugation.

##### Liquid chromatography separation and tandem mass spectrometry (LC-MS/MS)

Dried peptides were reconstituted in 2% ACN and 0.1% FA and analyzed by nanoflow liquid chromatography-tandem mass spectrometry (nLC-MS/MS) using a Neo Vanquish UHPLC interfaced with an Orbitrap Exploris 480 mass spectrometer (ThermoFisher Scientific). Peptide separation was performed with a linear gradient (water/acetonitrile) over a polyimide-coated, fused-silica, 25 cm × 360 μm o.d./75 μm i.d. self-packed PicoFrit column (New Objective) with a built-in emitter (75 μm emitter i.d.). Stationary phase in the analytical column consisted of ReproSil-Pur 120 C18-AQ, 2.4 μm particle size, 120 Å pore (Dr. Maisch High Performance LC GmbH). The trap column consisted of ~1 cm × 360 μm o.d./75 μm i.d. polyimide-coated, fused-silica tubing (New Objective), packed with 5 μm particle size, 120 Å pore, C18 stationary phase (ReproSil-Pur), with a Kasil frit. Electrospray ionization was accomplished with 2.1 kV positive spray voltage and an ion transfer tube temperature of 250°C.

For data-independent acquisition (DIA), peptides were separated by a 100-minute linear gradient. MS1 scans were acquired in the Orbitrap detector of the Exploris 480 mass spectrometer from 300-1250 m/z with the following settings: RF lens setting of 50%, 120,000 resolution at 200 m/z, normalized AGC of 101%, maximum injection time set to Auto, and Easy-IC internal lockmass calibration turned on. Precursor ions in each window were fragmented by HCD at 30% NCE. DIA product ion (MS2) scans were acquired in the Orbitrap detector using the following settings: 140–1800 m/z, 30,000 resolution at 200 m/z, normalized AGC of 1000%, and a maximum injection time of 54 ms.

##### Data analysis

Database searches were done using DIA-NN 2.1.0.^121^ Data were searched against a human UniProt FASTA database (accession UP000005640, 20,402 entries (reviewed Swiss-Prot canonical proteins), custom FASTA files containing each bait protein (GFP and SidL-H571A), and a custom contaminants file (to include 3xFLAG peptide) to generate an *in silico* spectral library. Search parameters were tryptic cleavage (maximum 1 missed), peptide length 7-30, precursor charge range 2-6, precursor m/z range 300-1250, fragment ion m/z range 140-1800, and N-term methionine excision and cysteine carbamidomethylation as fixed modifications (no variable modifications). Algorithm parameters were mass accuracy of 8ppm, MS1 accuracy of 2ppm, and scan window of 6 with Match Between Runs (MBR) and Protein Inference enabled. All other settings were default and output was filtered at a 1% false discovery rate (FDR). The resultant protein group file was manually filtered to remove contaminant proteins and the differential expression analysis was performed using DIA-Analyst (https://analyst-suites.org)^122^ with Bayesian Principal Component Analysis (BPCA) imputation. Volcano plots were visualized in Rstudio using ggplot2.^99^ Processed data are listed in Table S2. The mass spectrometry proteomics data have been deposited to the ProteomeXchange Consortium via the PRIDE partner repository with the dataset identifier PXD068090.^123^

#### Immunoblotting

The protein concentration of clarified lysates was determined using bicinchoninic acid (BCA) assays (Pierce #23227), according to the manufacturer’s instructions, measured on a Synergy H1 microplate reader (BioTek). Samples were normalized according to protein concentration and mixed with a 6X Laemmli loading buffer and heated at 95°C for ~5 minutes. Samples were loaded onto 4-20% 18-well or 26-well Criterion TGX Precast Midi Protein Gels (Bio-Rad #5671094 and #5671095) with Precision Plus Protein Dual Color Standard (Bio-Rad #1610374) as a ladder. 25 μg total protein was loaded for signaling experiments and 40 μg total protein was loaded for puromycin incorporation experiments. Proteins were separated at 200 V for 45 minutes in 1X Tris/Glycine/SDS buffer (Bio-Rad #1610732) then transferred to nitrocellulose membrane (Bio-Rad #1704271) using a Trans-blot Turbo Transfer System (Bio-Rad) at a constant 25 V for 30 minutes with a 1.0 amps limit.

Membranes were cut according to target protein size ranges and blocked in 5% Blotto non-fat milk (Santa Cruz #sc-2324) dissolved in TBS (20 mM Tris-HCl pH 7.5, 150 mM NaCl) for at least 20 minutes at room temperature. Membranes were then incubated with primary antibodies overnight at 4°C in 5% TBST (TBS with 0.1% Tween-20). The next day, membranes were washed thrice with TBST then incubated with secondary antibody diluted in 5% milk TBST for 1 hour at room temperature. After, membranes were washed thrice with TBST. For chemiluminescent-based detection, incubated with SuperSignal West Pico PLUS (ThermoFisher Scientific #34580) or Femto Maximum Sensitivity (ThermoFisher Scientific #34095) Chemiluminescent Substrate. Signal was detected using a ChemiDoc Imaging System (Bio-Rad). For anti-AMP,^55^ membranes were blocked in ROTI Block (Carl Roth #A151) diluted 1:10 in TBS, the primary antibody was diluted in ROTI Block diluted 1:10 in TBST supplemented with 1 mM MnCl_2_, and the secondary antibody was diluted in TBST without milk then imaged via chemiluminescence. For fluorescent-based detection (Fig. S2D only), membranes were blocked in Intercept TBS Blocking Buffer (LICORbio #927-60001) and antibodies were diluted in Intercept T20 TBS Antibody Diluent (LICORbio #927-65001). Fluorescence was detected using an Odyssey CLx (LICORbio). Antibodies used in this study are listed in KEY RESOURCES TABLE.

#### Purification of recombinant proteins

*Escherichia coli* BL21(DE3) cells (New England Biolabs #C2527I) were transformed with pGEX-3C-derived plasmids (pJB193, pJB194, pJB209, and pJB213) and cultured in Luria Broth (Research Products International #L24040) supplemented with 100 μg/mL carbenicillin (Research Products International #C46000) overnight at 37°C with shaking. 10 mL of saturated overnight culture was used to inoculate 1 L 2xYT media (Research Products International #X15680) supplemented with 100 μg/mL carbenicillin and cultured at 37°C with shaking until OD 0.6-0.75. At this time, cultures were chilled on ice for 5-10 minutes then isopropyl-β-D-thiogalactoside (Gold Biotechnology #I2481C) was added to 1 mM and shaken overnight at 16°C (~20-24 hours). All subsequent steps were done using pre-chilled tubes/reagents and on ice or at 4°C unless otherwise specified. Cells were centrifuged at 4,000 xg for 10 minutes, transferred to a 50 mL conical, and centrifuged again at 4,000 xg for 5 minutes. Cells were either used immediately or frozen in liquid nitrogen and stored at −80°C.

Fresh or thawed cells were resuspended in 50 mL Buffer 1 (50 mM Tris-HCl pH 8.0, 100 mM NaCl, 1 mM EDTA pH 8.0 (Sigma-Aldrich #ED), 2 mM β-mercaptoethanol (Sigma-Aldrich #M6250)) supplemented with 1 tablet/100 mL cOmplete EDTA-free protease inhibitor cocktail (Roche #11873580001), a pinch of Lysozyme (Sigma-Aldrich #L6876), and 15 kU/μL ultra-pure Benzonase nuclease (Sigma-Aldrich #E8263)) and sonicated for a total of 2 minutes (5-second pulses with 15-second rests) at 50% amplitude using a 0.5-inch diameter horn (Branson Ultrasonics #101-147-049). Cell lysates were ultracentrifuged on a Type 45 Ti fixed-angle rotor (Beckman Coulter #339160) at 40,000 rpm for 45 minutes. While samples were being clarified, 0.5-1mL Glutathione resin (Genscript #L00206) was pre-equilibrated with Buffer 1. Clarified lysates were incubated with glutathione resin for 1 hour with gentle nutation. Glutathione resin was then transferred to a 15 mL column and washed with 20 column volumes of Buffer 1.

For GST-3C-3xFLAG-tagged GFP, SidL, and SidL-H571A purifications, the glutathione resin was resuspended in 4 column volume 3C elution buffer (Buffer 1 with 2 mM DTT (Gold Biotechnology #DTT) and 26 μg/mL homemade 3C protease) and incubated overnight with gentle nutation to elute the proteins via GST-tag removal. For GST-3C-3xFLAG-LnaB, the glutathione resin washed with 6 column volumes of glutathione elution buffer (Buffer 1 with 10 mM glutathione (Sigma-Aldrich #G4251)) to elute the protein (GST-tag cleavage made 3xFLAG-LnaB insoluble). Eluates were concentrated with Amicon Ultra-4 or Ultra-15 filters (Millipore #UFC8010, #UFC8030, and #UFC9030; 10 kDa MWCO for GFP, 30 kDa MWCO for SidL, SidL-H571A, and LnaB), per manufacturer's instructions. Concentrated eluates were further purified by size-exclusion using a Superdex 200 Increase 10/300 GL column (Cytiva #28-9909-44) with an Akta pure FPLC (flow rate = 0.5 mL/minute). Size-exclusion was done using Buffer 2 (50 mM Tris-HCl pH 7.5, 150 mM NaCl, 5% glycerol, 2 mM DTT). Peak fractions were pooled and either diluted or concentrated on Amicon Ultra filters, as above. Protein concentrations were determined with a nanodrop (ThermoFisher Scientific) and calculated extinction coefficients. 20 μM aliquots of each protein were made and frozen in liquid nitrogen then stored at −80°C.

#### *In vitro* assays using radioactive ATP

##### Protein adenylation and ATPase activity assays

20 μL reactions were assembled containing 100 nCi/μL ATP[α^32^P] (Revvity #BLU503H250UC), 600 nM recombinant protein, and 1.2 μM actin (Cytoskeleton #APHL99) in reaction buffer (50 mM HEPES-KOH pH 7.4, 100 mM KOAc, 15 mM Mg(OAc)_2_). Note that actin was stored in general actin buffer (5 mM Tris-HCl pH 8.0, 200 μM CaCl_2_, 500 μM DTT, and 200 μM ATP). For reactions lacking actin, an equivalent volume of general actin buffer was added as a buffer control. Reactions were initiated by the addition of the recombinant and incubated for 30 minutes at 37°C with shaking at 550 rpm on a thermal mixer then placed on ice. Reactions were then split in half and separated by SDS-PAGE to access protein adenylation activity (Fig. 3A) or thin-layer chromatography (TLC) (Fig. 3B) to access ATPase activity.

For SDS-PAGE, Laemmli buffer was added to 1X, heated at 95°C for ~3 minutes, then separated on a 4-20% 18-well Criterion TGX Precast Midi Protein Gels with Precision Plus Protein Dual Color Standard at 200 V for 45 minutes in 1X Tris/Glycine/SDS buffer. The gel was fixed and Coomassie stained/destained and imaged on a ChemiDoc Imaging System. The gel was then dried using a Model 583 Gel Dryer (Bio-Rad) for 1-2 hours at 70°C.

For TLC analysis, 0.25 units of apyrase (New England Biolabs #M0398) was added to a 20 μL reaction and incubated for 30 minutes at 37°C to use as a marker for AMP. 0.5 μL of the reactions were spotted on a PEI Cellulose F TLC plate (Supelco #1.05579) and separated using a 500 mM KH_2_PO_4_ pH 3.5 mobile phase.

Dried gels and TLC plates were exposed overnight to a phosphor screen (Cytiva #BAS-IP-MS-2040-E) and imaged on an Amersham TYPHOON (Cytiva).

##### Pyrophosphate release assays

20 μL reactions were assembled as above except 50 nCi/μL ATP[ɣ^32^P] (Revvity #BLU502A250UC) was used. For adenylate cyclase-containing reactions, 0.3 ng recombinant *Bordetella pertussis* adenylate cyclase (BioTechne #8270-AC) and 0.2 μg Bovine testes calmodulin (Sigma-Aldrich #P1431) was used. Reactions were initiated by addition of recombinant protein and incubated for 30 minutes at 37°C then placed on ice. 50 units of *Saccharomyces cerevisiae* pyrophosphatase (Sigma-Aldrich #I1643) were added to the indicated reactions and incubated for an additional 15 minutes at 37°C. Samples were quenched with 20% trichloroacetic acid (Fisher Scientific #18-607-543) and 0.5 μL of each reaction was spotted onto a PEI Cellulose F TLC plate and separated using a 1.5 M KH_2_PO_4_ pH 3.4 mobile phase. (see Fig. 3C)

##### Metabolite library and sub-pooling adenylation reactions

10 μL reactions were prepared with 50 nCi/μL ATP[α^32^P], 100 nM SidL, and 600 nM actin or general actin buffer in reaction buffer (50 mM HEPES-KOH pH 7.4, 100 mM KOAc, 15 mM Mg(OAc)_2_) supplemented with a library of 696 metabolites (final concentration of each metabolite was 500 nM) or a metabolite library storage buffer (final concentration was 0.5 mM HEPES-KOH pH 7.4, 10 mM NaCl, 100 μM MgCl_2_, 0.4% DMSO). Reactions were initiated by the addition of SidL and incubated for 20 minutes at 37°C. 1 μL of each reaction was spotted onto a PEI Cellulose F TLC plate and separated using a 500 mM KH_2_PO_4_ pH 3.5 mobile phase.

For the metabolite library sub-pooling experiments, reactions were set up as above except metabolite concentrations were added at 40 μM for the first iteration (Fig. S3A) and 200 μM for the second and third iterations (Figs. S3B-C). Reactions were incubated for 60 minutes at 37°C prior to spotting onto PEI Cellulose F TLC plates.

To compare adenylation of compounds with similar structural features as 3-phosphoglycerate (Fig. S5), 200 μM of the indicated compounds were used and TLCs were separated using 500 mM or 750 mM KH_2_PO_4_ pH 3.5 mobile phases.

The metabolite library compounds are listed in Table S3 with corresponding 384-plate well locations and commercial sources. Note that this library is expanded from its original iteration and contains more metabolites than previously reported.^67^

##### 3PG adenylation assays

10 μL reactions were prepared as in “*Protein adenylation and ATPase activity assays*”, except 100 nM recombinant protein, ± 600 nM actin, 25 nCi/μL ATP[α^32^P], with or without 200 μM 3-phosphoglycerate were used. Reactions were initiated by addition of recombinant protein and incubated for 60 minutes at 37°C then placed on ice before being spotted on TLC plates (Figs. 4C and S3D). Reactions were subsequently split in half and supplemented with either 0.5 μL rSAP (New England Biolabs #M0371) or rSAP storage buffer (25 mM Tris-HCl pH 7.5, 1 mM MgCl_2_, 50% glycerol) and incubated for an additional 15 minutes at 37°C to test for rSAP susceptibility (Fig. S4D). 1 μL of each reaction was spotted onto a PEI Cellulose F TLC plate and separated using a 750 mM KH_2_PO_4_ pH 3.5 mobile phase.

##### Kinetics analysis of SidL ± 3PG

75 μL reaction mixtures were prepared containing reaction buffer, 600 nM actin, 25 nCi/μL ATP[α^32^P], 2 mM ATP (Sigma-Aldrich #A7699), with or without 20 mM 3-phosphoglycerate. Reactions were initiated by addition of 100 nM SidL and incubated for the indicated times at 37°C. Reactions were quenched at each time point by transferring 4.5 μL to a pre-chilled tube containing 0.5 μL 100% TCA (final concentration ~10%). The TCA was neutralized by adding 15 μL 1 M Tris-HCl pH 8.0 (final concentration 750 mM) prior to spotting 1 μL of each reaction onto a PEI Cellulose F TLC plate and separated using a 500 mM KH_2_PO_4_ pH 3.5 mobile phase. (see Fig. 4D)

Note that 3-phosphoglycerate used in these experiments were obtained from multiple vendors due to temporary unavailability (Sigma-Aldrich #P8877; Santa Cruz Biotechnology #sc-214793; MedChem Express #HY-141412). Small molecule structures were generated using ChemDraw (Revvity Signals Software).

#### *In vitro* translation assays

250 μl 1:1 rabbit reticulocyte lysate (RRL, Green Hectares) was depleted of globin proteins by incubation with 500 μl NiNTA resin (GoldBiotechnology #H-350-50) for 30 min at 4°C. The unbound fraction was collected by centrifugation through a 0.22 μm Costar Spin-X filter (Corning #CLS8161-100EA) and used for *in vitro* translation (IVT) reactions. IVT was performed as previously described.^124^ 10 μl reactions were assembled with 35 % (v/v) globin depleted RRL, 15 % (v/v) IVT buffer (133 mM HEPES pH 7.5, 333 KOAc, 6.66 mM ATP, 6.66 mM GTP, 80 mM creatine phosphate, 266 μg/ml creatine kinase, 0.66 mg/ml total pig liver tRNA, 13.3 mM Mg(OAc)_2_, 6.66 mM reduced glutathione, 2 mM spermidine, 267 μM each amino acid (without methionine)), 25 % (v/v) SidL or mutant at the indicated concentration, 25 % (v/v) ATP/Mg(OAc)_2_, 3PG, or water at the indicated concentrations. Reactions were mixed on ice, moved to 32°C and started by addition of 0.25 μl methionine[^35^S] EasyTag EXPRESS35S Protein Labeling Mix (Revvity #NEG772002MC). Reactions were stopped after 30 min by addition of 4X Laemmli buffer. Translated species were resolved by SDS-PAGE. Gels were coomassie stained then dried onto filter paper, exposed to a phosphor screen, and imaged by autoradiography using an Amersham TYPHOON imager (Cytiva).

#### Metabolite analysis by LC-MS

##### Analysis of in vitro reactions

Recombinant SidL *in vitro* activity was assessed in 100 μL reactions containing 0.2 mM ATP, 1 mM 3-phosphoglycerate, and 120 nM actin diluted in reaction buffer (5 mM HEPES-KOH pH 7.4, 10 mM KOAc, 1.5 mM Mg(OAc)_2_) with or without 20 nM wild-type SidL or SidL-H571A. Reactions were initiated by the addition of enzyme and incubated at 37°C with shaking at 750 rpm on a thermal mixer for 60 minutes. Reactions were quenched with 400 μL of cold methanol and vortexed for 30 seconds. To test the presence of labile phosphate groups on the products of SidL, *in vitro* reactions were split in half and were treated with either 1 μL rSAP or storage buffer for 30 mins prior to quenching with 200 μL of cold methanol. Quenched reactions were centrifuged at max speed for 10 minutes at 4°C to pellet insoluble debris. 2 μL of supernatant was injected and analyzed by liquid chromatography and mass spectrometry (LC-MS).

##### Extraction of metabolites from HEK293T cells

For cell metabolomics experiments, five sets of paired 6-well plates were seeded with 4x10^5^ HEK293T cells per well (one plate for metabolite extraction and another for cell counts). Paired plates were seeded by making five 2x10^5^ cells/mL mixtures and 2 mL aliquots of this mixture were added to each well. DMEM media (Gibco #11965092; 4.5 g/L D-glucose, 584 mg/L L-glutamine, no pyruvate) supplemented with 10% dialyzed FBS (Gibco #26400044) was used for this experiment. Cells were transfected ~28 hours after seeding with 1 μg of pDNA, as described in “Plasmid DNA transfections”, and 0.15% DMSO or 300 nM Torin 1 was added 2 hours post-transfection. After overnight incubation (~16 hours), media was exchanged with fresh 2 mL media containing DMSO or Torin 1 and incubated for 2 hours at 37°C.

All subsequent steps were done using ice-cold tubes/reagents on ice or at 4°C unless otherwise noted. Cells were washed twice on with Blood Bank Saline Isotonic Solution 0.90% w/v (ThermoFisher Scientific #293-184) then lysed in 500 μL extraction solvent (80:20 v/v methanol (Sigma-Aldrich #34860) and HPLC-grade water (Sigma-Aldrich #270733)). Cells were then quickly scraped and transferred to fresh tubes, vortexed for 15 minutes, then centrifuged at 18,123 xg for 10 minutes. 350 μL of each supernatant was transferred to a fresh tube and dried under nitrogen gas at room temperature on a Reacti-Therm III Heating/Stirring Module (Pierce) prior to −80°C storage and shipped on dry ice. The cells in the paired plate were washed twice with Blood Bank Saline, trypsinized, and counted on a TC20 Automated Cell Counter (Bio-Rad) using trypan blue (Bio-Rad #1450021).

On the day of LC-MS analysis, cell extracts were reconstituted in 100 μL of cold water:acetonitrile (1:1, v/v) and vortexed at 4°C for 10 minutes. Samples were centrifuged at max speed for 10 minutes at 4°C to pellet any insoluble debris. Supernatants were transferred to glass analytical vial inlets for analysis. A pooled sample for quality control was prepared by pooling 2.5 μL of each sample. All samples were queued for LC-MS analysis in random order. 3 μL of each sample was injected and analyzed by LC-MS.

##### LC-MS analysis of in vitro reactions and metabolites from HEK293T cells

LC-MS analysis of polar metabolites was achieved using a Dionex Ultimate 3000 UPLC system (ThermoFisher Scientific) coupled to a QExactive Orbitrap mass spectrometer (ThermoFisher Scientific) equipped with a heated electrospray ionization (HESI) source operating in both positive- and negative-ion modes. Samples were queued in an autosampler chamber set to 8°C. In random order, samples were injected onto a SeQuant ZIC-pHILIC column (2.1 mm x 150 mm; 5 μm particle size) (Millipore Sigma) held at 30°C and were eluted with 20 mM ammonium carbonate, 0.1% ammonium hydroxide (mobile phase A) and acetonitrile (mobile phase B) as the solvents. The chromatography of the elution was performed as follows: 0-20 mins, linear gradient from 80% B to 20% B; 20-20.5 mins, linear gradient from 20% B to 80% B; 20.5 – 28 mins, 80% B. From 21 – 28 mins, flow was diverted to waste. The flow rate was held at 150 μL/min. Samples were ionized using an HESI source kept at 320°C. The sheath gas flow rate was set to 50 units, the aux gas flow rate was set to 15 units, and the sweep gas flow rate was set to 3 units. Spray voltages for positive- and negative-ion modes were set to 4100 and 3300, respectively. Radio frequency lens of the ion transfer tube was set to 70%.

For *in vitro* reactions, targeted quantification of reactants and products (see Table S4 for target ion masses and retention times) was achieved using a targeted single ion monitoring (SIM) method in negative-ion mode with the following parameters: Resolution at 70000 full width of half maximum, automatic gain control target of 5x10^4^, maximum injection time of 100 ms, and isolation window of 4 m/z. Data dependent MS2 scans of 2-AMP-3PG ([M-H]^−^ = 514.0382) and 2-AMP-glycerate ([M-H]^−^ = 434.0719) were achieved with the following parameters: Resolution at 35000 resolution full width of half maximum, automatic gain control target of 2x10^5^ with a minimum of 8x10^3^, maximum injection time of 100 ms, top N = 5, isolation window of 2.0 m/z, and collision energy set to 35. Bar plots were generated in GraphPad Prism and analyzed using a two-way ANOVA with Turkey’s multiple comparisons correction to calculate *p*-values.

Cellular extracts were analyzed using a method that includes a full scan from to 70 to 1050 m/z in both positive-and negative-ion modes and a SIM scan in negative-ion mode for targeted quantification of 2-AMP-3PG and 2-AMP-glycerate. Both sets of scans operated under the following parameters: Resolution of 70000 full width of half maximum, maximum injection time of 100 ms, isolation window of 2.0 m/z, and automatic gain control target of 1x10^6^. For cellular metabolomics, metabolites were identified by referencing a library of external standards. Relative quantification of metabolite abundance was performed using Xcalibur 4.1.50 software (ThermoFisher Scientific). A mock extraction control was used to subtract out background noise. Raw peak intensities were normalized to total cell number. Poorly detected molecules (undetected in >40% of samples, unless missing values were confined to specific groups) were removed from the final dataset.

For 2-AMP-3PG, bar graphs were visualized using GraphPad Prism and ordinary one-way ANOVA with Turkey’s multiple comparisons correction was used to calculate *p*-values. For pairwise comparisons for untargeted metabolomics, log2(fold change) and *p*-values using a two-sided Student’s *t* test were calculated in Rstudio and volcano plots were visualized using ggplot2.^99^ MS2 spectra were visualized in FreeStyle 1.3 SP2 (ThermoFisher Scientific). Metabolomics data for *in vitro* SidL reaction are listed in Table S4 and the metabolomics data and statistics for the cellular metabolomics are listed in Table S5. LC-MS metabolomics mass spectrometry .raw files have been uploaded to the MassIVE repository under the accession number MSV000099170. Raw file names for corresponding samples are defined in Tables S4 and S5.

##### Extraction of metabolites from L. pneumophila-infected THP-1 cells

Wild-type bacteria (strain Lp02) containing pJB908 (an empty vector)^125^ (TO222),^126^ a Dot/Icm translocation-deficient mutant (strain Lp03) containing pJB908 (TO183),^126^ and a Δ*sidL* mutant (TO4654) harboring either pJB908 (TO4898), pDTI116::3xFLAG-sidL (pJB245) (TO4901) or pDTI116::3xFLAG-*sidL-H571A* (pJB246) (TO4904), were grown to mid-exponential phase, induced with 0.25 mM IPTG for 2 hours, and grown to post exponential phase (based on A600 = 3.8 to 4.0 and demonstrated motility based on visual inspection using a Nikon TS100 inverted microscope). To verify protein expression, 3x10^9^ bacteria were resuspended in 150 μL of 1X Laemmli buffer and boiled for 10 min. To verify expression of 3xFLAG-tagged proteins, equal volumes of sample were examined by immunoblotting (as describe in “Immunoblotting”) with Ponceau S staining of the membrane as a loading control. For infection, 2x10^7^ THP-1 cells were seeded in a 10-cm dish in medium containing 2-O-tetradecanoylphorbol-13-acetate (10 ng/ml; TPA) and incubated for 48 hours. THP-1 cells were then rinsed with fresh media lacking TPA and challenged with bacteria at a multiplicity of infection (MOI) of 5 for 1 hour. Cells were rinsed three times with medium lacking TPA. Uninfected cells were treated the same but without bacterial challenge.

All subsequent steps were performed on ice or at 4°C unless otherwise noted. At 1, 3, 6, and 9 hours post infection, adherent and detached cells were harvested and resuspended in ice-cold extraction solvent (80:20 v/v methanol:HPLC-grade water containing 500 nM isotopically labeled adenosine monophosphate-^13^C_10_,^15^N_5_ (MedChem Express #HY-A0181S)). To harvest detached cells, culture supernatant was harvested and centrifuged at 1,000 x*g* for 5 minutes at 4°C. Cells were washed twice with 1 mL Blood Bank Saline Isotonic Solution 0.90% (w/v) then lysed in 100 μL of ice-cold extraction solvent and vortexed briefly. In parallel, the adherent cells were washed twice with 5 mL Blood Bank Saline Isotonic Solution 0.90% (w/v) then lysed in 900 μL extraction solvent, harvested quickly by scraping, transferred to a new tube, and vortexed briefly before being combined with the detached cell lysate. Cell lysates were vortexed for 15 minutes then centrifuged at 18,123 x*g* for 10 minutes. 900 μL of each supernatant was transferred to a fresh tube and dried under nitrogen gas at room temperature on a Reacti-Therm III Heating/Stirring Module prior to storage at −80°C. The remaining insoluble pellet was flash frozen in liquid nitrogen and stored at −80°C to be quantified by BCA analysis for normalization purposes.

For BCA analysis, the insoluble pellet was thawed and resuspended in 500 μL 0.2 M NaOH and heated at 95°C for 20 minutes with shaking. Samples were then diluted with 0.2 M NaOH to a final volume of 1,400 μL. 25 μL of each sample was then analyzed in technical duplicates by BCA analysis, as described above in the “Immunoblotting” subsection.

##### LC-MS analysis of metabolites from L. pneumophila-infected THP-1 cells

Metabolite extracts were analyzed using a 1290 Infinity III LC coupled to an 6495D triple quadrupole mass spectrometer (Agilent). The LC was equipped with a ZORBAX RRHD Extend C18 column (2.1 × 150 mm, 1.8 μm) (Agilent, 759700-902) held at 35°C. Metabolites were injected onto the column and eluted with water/methanol (97:3) containing 10 mM tributylamine and 15 mM acetic acid (mobile phase A) and methanol containing 10 mM tributylamine and 15 mM acetic acid (mobile phase B). Both mobile phases contained 0.01% InfinityLab deactivator additive (v/v) (Agilent Technologies, 5191-4506). The LC gradient was performed as follows: 0% B from 0 mins to 2.5 mins; 20% B at 7.5 mins, 45% B at 13 mins, 99% B at 20 mins; 99% B to 0% B from 32.1 mins to 35 mins; 0% B from 35 mins to 38 mins (end of run). Flow rates were set as follows: 0.25 mL/min from 0 mins to 24.05 mins; 0.4 mL/min from 24.05 mins to 32 mins; 0.25 mL/min from 32.1 mins to 35 mins; 0.4 mL/min from 35.1 mins to 37.9 mins; 0.25 mL/min at 38 mins. To clean column in-between runs, column was run in reverse from 24 mins to 37.9 mins. Sample replicates were reconstituted and analyzed as separate batches across four days. During the runs, samples were stored in an autosampler chamber set to 4°C. 2 μL of each sample in each batch were injected in random order and were analyzed within 24-48 hours of reconstitution.

The MC operated in negative-ion mode with the following parameters: drying gas set to 150°C at 13 L/min, nebulizer at 45 psi, sheath gas set to 325°C at 12 L/min, capillary voltage at 2000 V, nozzle voltage at 500 V, fragmentor at 166 V. Targeted measurement of metabolites was performed using a multiple reaction monitoring (MRM) method. A library of transitions, explicit retention times, and optimal collision energies were generated using authentic standards or SidL *in vitro* reactions when necessary. Agilent MassHunter Acquisition Software (v12.3) was used for method setup and data collection and metabolite peak areas were quantified using Skyline (v25.1). To account for general extraction variability and differences in cell density, raw peak intensities were normalized to the isotopically labeled AMP internal standard and total protein mass (μg). MRM parameters and data collected from these experiments are described in Table S6.

Bar plots were generated in GraphPad Prism and analyzed using a two-way ANOVA with Turkey’s multiple comparisons correction to calculate *p*-values for comparison across timepoints for each strain (for Fig. 6) and using a two-way ANOVA with Dunnett’s multiple comparisons correction to calculate *p*-values for comparisons across strains within the same timepoint (for Figs. S8B-I and S9).

#### Glucose consumption/lactate production assays

5x10^5^ HEK293T cells per well were seeded in a 6-well dish in 2 mL media; 6-well dishes were cultured in technical duplicate. DMEM media (Gibco #11966025; no glucose, 584 mg/L L-glutamine, no pyruvate) supplemented with 10% FBS and 2.7 g/L (15 mM) D-glucose (Sigma-Aldrich #G7021) was used for these experiments. Cells were transfected ~28-30 hours after seeding with 1 μg of pDNA, as described in “Plasmid DNA transfections”, and 0.15% DMSO, or 300 nM Torin 1 was added 2 hours post-transfection. After overnight incubation (~16-18 hours), media was exchanged with fresh media containing DMSO, or Torin 1. An aliquot of media supernatant from each well was retained (T0), flash frozen, and stored at −80°C. Cells were then incubated for 6 hours and another aliquot of media supernatant was taken from each well (T6), flash frozen, and stored at −80°C. Cells were then trypsinized and counted on a TC20 Automated Cell Counter (Bio-Rad) using trypan blue. The amounts of glucose and lactate in the T0 and T6 supernatants were determined using clear, flat-bottom 96-well plate-based assays measured on a Synergy H1 microplate reader (BioTek).

To measure glucose, media supernatants were thawed and diluted 2-fold in DMEM without glucose and 5 μL of diluted supernatants were added to a well and mixed with 150 μL glucose reagent (μDialysis #P000023). This was incubated for 10 minutes at room temperature then measured at 546 nm. Samples were compared to a standard curve of 2-fold serially diluted glucose standards (15-0.47 mM) made in the same media and linearly interpolated using GraphPad Prism. Glucose consumed per hour per 10^6^ cells was calculated by first multiplying the measured glucose concentrations by 2 then subtracting T6 from T0 then dividing by 6 hours then dividing by cell count.

To measure lactate, media supernatants were thawed and diluted 15-fold in water. 20 μL of diluted supernatants were added to a well and 50 μL Lactate Reagent (Eton Bioscience #1200012002). This was incubated for 60 minutes at 37°C then measured at 490 nm. Samples were compared to a standard curve of 2-fold serially diluted lactate standards (3000-47 μM) made in water and linearly interpolated using GraphPad Prism. Lactate produced per hour per 10^6^ cells was calculated by multiplying the measured lactate concentrations by 15 then subtracting T0 from T6, dividing by 6 hours then dividing by the corresponding cell count.

A mean was calculated for the technical duplicates of each biological replicate. These data were plotted in GraphPad Prism and analyzed with an ordinary one-way ANOVA with Turkey’s multiple comparisons correction to calculate *p*-values.

#### Analysis of the SidL product by NMR

A 2.5 mL mixture containing 100 nM recombinant SidL, 600 nM actin, 10 mM 3PG (Santa Cruz #sc-214793), and 10.5 mM ATP (Sigma A7699) in reaction buffer (50 mM HEPES-KOH pH 7.4, 100 mM KOAc, 15 mM Mg(OAc)_2_) was assembled. This mixture was split into 21 120 μL aliquots and incubated at 37°C for 3 hours with shaking at 550 rpm on a thermal mixer. The reaction aliquots were then pooled together and filtered through 10 kDa cutoff centrifugal filter tubes (VWR #82031-348) to remove SidL and actin. Reactions were flash frozen in LN_2_ and lyophilized to remove water prior to analysis by nuclear magnetic resonance (NMR).

For 1D ^1^H-NMR, 1D ^31^P-NMR, and ^1^H-^31^P HMQC NMR, crude samples were dissolved in 550 μL D_2_O and transferred to NMR tubes for spectra collection. A glass insert, containing TPPO dissolved in benzene-*d*6, was used as an external reference set to 0.0 ppm for ^31^P experiments. For 1D ^13^C-NMR, ^1^H-^1^H TOCSY NMR, and ^1^H-^13^C HSQC NMR, flash chromatography was performed to remove excess salts including acetate from the buffer that may interfere with spectra collection. After filtering and lyophilization, crude product was dissolved in a minimum of water and run on a silica flash column (Luknova SuperSep 12 g silica column, Biotage Isolera One) using the following solvent system A: Acetonitrile, B: Water + 0.1% TEA. The following gradient was run: 5% B for 3 CV, 5-100% B over 15 CV, 100% B for 3 CV. Product elution was monitored by absorbance at 254 nm. Product eluted after ~9 CV at 40% B in a single peak. Fractions were collected and flash frozen in LN_2_ and lyophilized to remove water/acetonitrile. The resulting white solid was dissolved in 550 μL of D_2_O and transferred to an NMR tube for spectra collection. Control samples of 3PG and AMP were prepared in reaction buffer, flash frozen in LN_2_, lyophilized, and dissolved in 550 μL D_2_O. 1D ^1^H-NMR, 1D ^31^P-NMR, and ^1^H-^31^P HMQC NMR were collected on a JEOL JNM-ECZL500R 500 MHz spectrometer at room temperature (~22°C). 1D ^13^C-NMR, 2D ^1^H-^1^H TOCSY NMR, and 2D ^1^H-^13^C HSQC NMR were collected on an 800 MHz Bruker Avance NEO equipped with a triple resonance TCI cryogenic probe with actively shielded z-axis gradients at 15°C. All data were processed in Bruker TopSpin 4.4.1.

- ^1^H-NMR: (500 MHz, D_2_O) δ 8.31 (s, 1H), 8.04 (s, 1H), 5.93 (d, *J* = 6.0 Hz, 1H), 4.57 (t, *J* = 5.7 Hz, 1H), 4.43 (p, *J* = 4.6 Hz, 1H), 4.32 (t, *J* = 3.7 Hz, 1H), 4.21 (t, *J* = 2.6 Hz, 1H), 4.02 (m, 2H), 3.88 (m, 2H)
- ^13^C-NMR: (200 MHz, D_2_O) δ 175.853, 155.399, 152.641, 148.979, 139.643, 118.353, 86.427, 84.089, 76.514, 74.061, 70.207, 65.943, 64.673
- ^31^P-NMR: (202 MHz, D_2_O) δ −22.192 (s), −25.579 (s)

### QUANTIFICATION AND STATISTICAL ANALYSIS

For all experiments, the details of quantification, statistical methods, and the software used are described in the corresponding figure legends and methods (as well as below).

For Fig. 2F, cell confluency was calculated using CELLCYTE Studio (Cytena, settings: contrast sensitivity 50 a.u., smoothing 2 a.u., filled hole size 100 μm^2^, minimum object size 100 μm^2^). Confluency values for both plots are mean ± standard deviation (SD) of biological triplicates (n=3) and an ordinary one-way ANOVA with Dunnet’s multiple comparisons was used to calculate *p*-values using GraphPad Prism for the 48-hour timepoint (right bar plot): *p* < 0.0001 (****).

For Fig. S2A, see the “Mass spectrometry of affinity-purifications” subsection of the methods for detailed description of the analysis of these proteomics data. Briefly, affinity purifications were done in technical triplicate (n=3) and mass spectrometry searches were done using DIA-NN 2.1.0.^121^ The resultant protein group file was manually filtered to remove contaminant proteins and the differential expression analysis was performed using DIA-Analyst (https://analyst-suites.org)^122^ which calculates *p*-values using a moderated *t* test (Benjamini-Hochberg adjusted) with Bayesian Principal Component Analysis (BPCA) imputation applied. Volcano plots displaying log2(fold change between 3xFLAG-SidL-H571A and 3xFLAG-GFP) vs −log10(adjusted *p*-values) were visualized in Rstudio using ggplot2.^99^

For Figs. 4F and S4B-C, bars are mean signal area intensities ± SD with points representing individual reactions (n=3) and a two-way ANOVA with Turkey’s multiple comparisons was used to calculate *p*-values using GraphPad Prism: *p* ≤ 0.05 (*), *p* ≤ 0.01 (**), *p* ≤ 0.001 (***), *p* ≤ 0.0001 (****). Only relevant comparisons are shown and “nd” denotes not detected.

For Figs. 5A-B, signal area intensities for each molecule are normalized to cell counts; bars are mean normalized signal area intensities ± SD with points representing individual biological samples (n=5). Bar graphs were visualized using GraphPad Prism and an ordinary one-way ANOVA with Turkey’s multiple comparisons correction was used to calculate *p*-values: *p* ≤ 0.001 (***). “nd” denotes not detected.

For Figs. 5D-E, quantification of glucose and lactate production are discussed in detail in the “Glucose consumption/lactate production assays” subsection of the methods. Experiments were done in biological replicates (n=4) with each biological replicate being done in technical duplicate. A mean was calculated for the technical duplicates of each biological replicate. These data were plotted in GraphPad Prism and analyzed using an ordinary one-way ANOVA with Turkey’s multiple comparisons correction to calculate *p*-values: not significant (ns), *p* ≤ 0.05 (*), *p* ≤ 0.01 (**), *p* ≤ 0.001 (***). Bars are mean ± SD of biological replicates (n=4) with points being the mean of technical duplicates for each biological replicate.

For Figs. 5F-G and S7B-D, detection and quantification of metabolites from HEK293T cells via untargeted metabolomics are detailed in the “LC-MS analysis of in vitro reactions and metabolites from HEK293T cells” subsection of the methods. For pairwise comparisons of conditions containing individual biological samples (n=5), log2(fold change) and *p*-values using a two-sided Student’s *t* test were calculated in Rstudio and volcano plots were visualized using ggplot2 for Figs. S7B-D.^99^ Metabolite changes are shown as log2-transformed fold changes and a −log10-transformed *p*-value calculated with a two-sided Student's *t* test (n=5). The dashed lines on the y- and x-axes delineate −log10(0.05) *p*-value and log2 fold change of ± 1, respectively. Figs. 5F-G are bar graphs of the same data specifically showing fold changes in the abundance of glycolysis and serine biosynthesis intermediates and asterisks (*) indicate a two-sided Student’s *t* test *p*-value ≤ 0.05.

For Figs. 6A-B, S8B-I, and S9, detection and quantification of metabolites from THP1 cells infected with or without *L. pneumophila* via targeted metabolomics are detailed in the “LC-MS analysis of metabolites from *L. pneumophila*-infected THP-1 cells” subsection of the methods. Metabolite signal areas are normalized to a heavy AMP standard in the extraction buffer and total cellular protein content. Bars are mean ± SD with points representing biological replicates (n=4). Bar plots were generated in GraphPad Prism and analyzed using a two-way ANOVA with Turkey’s multiple comparisons correction to calculate *p*-values for comparison across timepoints for each strain (for Figs. 6A-B) and using a two-way ANOVA with Dunnett’s multiple comparisons correction to calculate *p*-values for comparisons across strains within the same timepoint (for Figs. S8B-I and S9): not significant (ns), *p* ≤ 0.05 (*), *p* ≤ 0.01 (**), *p* ≤ 0.001 (***), *p* ≤ 0.001 (****).

## Supplementary Material

1 **Document S1.** Supplemental Figures S1-S9.

2 **Table S1.** Sequence Annotation and Similarity Network Scores. Related to Figs. 2G-H and S1.

3 **Table S2.** Proteomic composition of 3xFLAG-SidL-H571A and 3xFLAG-GFP affinity purifications. Related to Fig. S2A.

4 **Table S3.** Metabolite library compounds and sources. Related to Figs. 4B, S3A-C and S5.

5 **Table S4.** LC-MS analysis data for *in vitro* reactions. Related to Figs. 4E, 4F, S4B, S4C, and S6.

6 **Table S5.** Metabolomics analysis of HEK293T metabolites. Related to Figs. 5A, 5B, 5F-H and S7.

7 **Table S6.** LC-MS analysis of metabolites from *L. pneumophila*-infected THP-1 cells. Related to Figs. 6, S8, and S9.

8 **Data S1.** Newick tree file. Related to Figs. 2G-H.

9 **Data S2.** Additional NMR validation of 2-AMP-3PG. Related to Fig. 4G.

10 **Data S3.** GenBank files for the plasmids generated in this study.

## Figures and Tables

**Table:** Key resources table

REAGENT or RESOURCE	SOURCE	IDENTIFIER
Antibodies		
Mouse monoclonal anti-puromycin (1:2000)	Sigma-Aldrich	Cat#MABE343; RRID: AB_2566826
Rabbit monoclonal anti-β-actin-HRP (1:5000)	Cell Signaling Technology	Cat#5125; RRID: AB_1903890
Rabbit polyclonal anti-pan-actin (1:1000)	Cell Signaling Technology	Cat#4968; RRID: AB_2313904
Mouse monoclonal anti-vinculin (1:5000)	Santa Cruz Biotechnology	Cat#sc-73614; RRID: AB_1131294
Rabbit monoclonal anti-4EBP1 (1:1000)	Cell Signaling Technology	Cat#9644; RRID: AB_2097841
Rabbit polyclonal anti-phospho-p70 S6 Kinase Thr421/Ser424 (1:1000)	Cell Signaling Technology	Cat#9204; RRID: AB_2265913
Rabbit monoclonal anti-p70 S6 Kinase (1:1000)	Cell Signaling Technology	Cat#2708; RRID: AB_390722
Mouse monoclonal anti-FLAG M2-HRP (1:20,000)	Sigma-Aldrich	Cat#A8592; RRID: AB_439702
Mouse monoclonal anti-FLAG M2 (1:20,000)	Sigma-Aldrich	Cat# F1804; RRID: AB_262044
Mouse monoclonal anti-AMP 17G6 (1:1000)	Höpfner et al.^55^	N/A
Rabbit monoclonal anti-phospho-Acetyl-CoA Carboxylase Ser79 (1:1000)	Cell Signaling Technology	Cat#11818; RRID: AB_2687505
Rabbit monoclonal anti-Acetyl-CoA Carboxylase (1:1000)	Cell Signaling Technology	Cat#3676; RRID: AB_2219397
Rabbit polyclonal anti-AMPKα (1:1000)	Cell Signaling Technology	Cat#2532; RRID: AB_330331
Goat polyclonal anti-rabbit IgG-HRP (1:5000)	Cell Signaling Technology	Cat#7074; RRID: AB_2099233
Goat polyclonal anti-mouse IgG-HRP (1:5000)	Invitrogen	Cat#32430; RRID: AB_1185566
Goat polyclonal anti-mouse IgG-IRDye 680RD (1:20,000)	LICORbio	Cat#926-68070; RRID: AB_10956588
Goat polyclonal anti-rabbit IgG-IRDye 800CW (1:20,000)	LICORbio	Cat#926-32211; RRID: AB_621843
Bacterial and virus strains		
*Escherichia coli* DH5α	Invitrogen	Cat#18265017
*Escherichia coli* BL21(DE3)	New England Biolabs	Cat#C2527I
*Escherichia coli* DH5α λpir (endA1 glnV44 thi-1 recA1 relA1 gyrA96 deoR nupG (Φ80dlac ΔlacZ) M15 (ΔlacZYA-argF)U169, hsdR17(rK-mK+), (λpir))	Kolter et al.^98^	N/A
*Legionella pneumophila Philadelphia-1, rpsL, hsdR, thy-* (wild-type strain)	Berger and Isberg^74^	Lp02
*Legionella pneumophila Philadelphia-1, rpsL, hsdR, thy-, dotA03* (translocation deficient strain)	Berger and Isberg^74^	Lp03
*Legionella pneumophila* Lp02 Δ*sidL*	This paper	TO4654
*Legionella pneumophila* Lp02 + pJB908	O’Connor et al.^126^	TO222
*Legionella pneumophila* Lp03 + pJB908	O’Connor et al.^126^	TO183
*Legionella pneumophila* Lp02 Δ*sidL* + pJB908	This paper	TO4898
*Legionella pneumophila* Lp02 Δ*sidL* + pJB245	This paper	TO4901
*Legionella pneumophila* Lp02 Δ*sidL* + pJB246	This paper	TO4904
Chemicals, peptides, and recombinant proteins		
Fetal bovine serum	Gibco	Cat#26140079
Dulbecco’s Modified Eagle Medium (4.5 g/L D-glucose, 584 mg/L L-glutamine, 110 mg/L sodium pyruvate)	Gibco	Cat#11995065
Trypsin-EDTA (0.25%), phenol red	Gibco	Cat#25200114
Fetal bovine serum, dialyzed	Gibco	Cat#26400044
Dulbecco’s Modified Eagle Medium (4.5 g/L D-glucose, 584 mg/L L-glutamine, no pyruvate)	Gibco	Cat#11965092
Dulbecco’s Modified Eagle Medium (no D-glucose, 584 mg/L L-glutamine, no pyrvuate)	Gibco	Cat#11966025
D-glucose	Sigma-Aldrich	Cat#G7021
Advanced RPMI 1640 medium	Gibco	Cat#12633012
200 mM L-glutamine (100X)	Gibco	Cat#25030081
PBS, pH 7.4	Gibco	Cat#10010023
L-cysteine	Sigma-Aldrich	Cat#C7352
ferric nitrate	Sigma-Aldrich	Cat#216828
thymidine	Sigma-Aldrich	Cat#T1895
Blood Bank Saline Isotonic Solution 0.90% w/v	ThermoFisher Scientific	Cat#293-184
Mycoplasma PCR detection kit	Applied Biological Materials	Cat#G238
Lipofectamine 3000 Transfection Reagent	Invitrogen	Cat#L3000015
Opti-MEM Reduced Serum Media	Gibco	Cat#51985034
Gibson Assembly Master Mix	New England Biolabs	Cat#E2611
GeneJET Plasmid Miniprep Kit	ThermoFisher Scientific	Cat#K0503
ZymoPURE II Plasmid Midiprep Kit	Zymo Research	Cat#D4201
Dimethyl sulfoxide (DMSO)	Invitrogen	Cat#D12345
Torin 1	Selleckchem	Cat#S2827
Rapamycin	Selleckchem	Cat#S1039
Puromycin	Sigma-Aldrich	Cat#P7255
Emetine	Millipore	Cat#324693
RIPA buffer	ThermoFisher Scientific	Cat#89901
Halt Protease and Phosphatase Inhibitor Cocktail	ThermoFisher Scientific	Cat#71636
Na_2_HPO_4_	Sigma-Aldrich	Cat#71636
β-glycerophosphate	Sigma-Aldrich	Cat#G9422
Benzonase Nuclease	Millipore	Cat#E1014
TURBO Dnase	Invitrogen	Cat#AM22390
NP-40 alternative	Millipore	Cat#492018
Open-top polyclear 14x89 mm centrifuge tubes (for SW41)	Seton Scientific	Cat#7030
PD MiniTrap desalting column with Sephadex G-25 resin	Cytiva	Cat#28918007
ANTI-FLAG M2 magnetic beads	Millipore	Cat#M8823
3xFLAG peptide	Sigma-Aldrich	Cat#F4799
4-20% 18-well Criterion TGX Precast Midi Protein Gels	Bio-Rad	Cat#5671094
4-20% 26-well Criterion TGX Precast Midi Protein Gels	Bio-Rad	Cat#5671095
Precision Plus Protein Dual Color Standard	Bio-Rad	Cat#1610374
10X Tris/Glycine/SDS buffer	Bio-Rad	Cat#1610732
Trans-Blot Turbo RTA Midi 0.2 μm Nitrocellulose Transfer Kit	Bio-Rad	Cat#1704271
Blotto non-fat milk	Santa Cruz Biotechnology	Cat#sc-2324
SuperSignal West Pico PLUS Chemiluminescent Substrate	ThermoFisher Scientific	Cat#34580
Femto Maximum Sensitivity Chemiluminescent Substrate	ThermoFisher Scientific	Cat#34095
ROTI Block	Carl Roth	Cat#A151
Intercept TBS Blocking Buffer	LICORbio	Cat#927-60001
Intercept T20 TBS Antibody Diluent	LICORbio	Cat#927-65001
Luria Broth	Research Products International	Cat#L24040
2xYT Broth	Research Products International	Cat#X15680
carbenicillin	Research Products International	Cat#C46000
isopropyl-β-D-thiogalactoside (IPTG)	Gold Biotechnology	Cat#I2481C
Ethylenediaminetetraacetic acid (EDTA)	Sigma-Aldrich	Cat#ED
β-mercaptoethanol	Sigma-Aldrich	Cat#M6250
Tris(2-carboxyethyl)phosphine hydrochloride (TCEP)	Gold Biotechnology	Cat#TCEP2
Dithiothreitol (DTT)	Gold Biotechnology	Cat#DTT
cOmplete EDTA-free protease inhibitor cocktail	Roche	Cat#11873580001
Lysozyme	Sigma-Aldrich	Cat#L6876
ultra-pure Benzonase nuclease	Sigma-Aldrich	Cat#E8263
Glutathione resin	Genscript	Cat#L00206
Glutathione	Sigma-Aldrich	Cat#G4251
Amicon Ultra-4 Centrifugal Filter Unit, 10 kDa MWCO	Millipore	Cat#UFC8010
Amicon Ultra-4 Centrifugal Filter Unit, 30 kDa MWCO	Millipore	Cat#UFC8030
Amicon Ultra-15 Centrifugal Filter Unit, 30 kDa MWCO	Millipore	Cat#UFC9030
Superdex 200 Increase 10/300 GL column	Cytiva	Cat#28-9909-44
Actin protein ( >99% pure, human platelet beta gamma actin)	Cytoskeleton	Cat#APHL99
Recombinant Shrimp Alkaline Phosphatase (rSAP)	New England Biolabs	Cat#M0371
Apyrase	New England Biolabs	Cat#M0398
Recombinant *Bordetella pertussis* adenylate cyclase	BioTechne	Cat#8270-AC
Bovine testes calmodulin	Sigma-Aldrich	Cat#P1431
*Saccharomyces cerevisiae* pyrophosphatase	Sigma-Aldrich	Cat#I1643
PEI Cellulose F TLC plastic sheets	Supelco	Cat#1.05579
Trichloroacetic Acid, 100% (w/v) Aqueous Solution	Fisher Scientific	Cat#18-607-543
ATP[α^32^P] 3000Ci/mmol 10mCi/ml EasyTide	Revvity	Cat#BLU503H250UC
ATP[ɣ^32^P] 3000Ci/mmol 10mCi/ml EasyTide	Revvity	Cat#BLU502A250UC
EasyTag EXPRESS^35^S Protein Labeling Mix, [^35^S]-, 2mCi	Revvity	Cat#NEG772002MC
Adenosine 5’-triphosphate (ATP)	Sigma-Aldrich	Cat#A7699
Metabolite library	Hicks et al.^67^	N/A
3-phosphoglycerate	Sigma-Aldrich	Cat#P8877
3-phosphoglycerate	Santa Cruz Biotechnology	Cat#sc-214793
3-phosphoglycerate	MedChem Express	Cat#HY-141412
Methanol	Sigma-Aldrich	Cat#34860
HPLC-grade water	Sigma-Aldrich	Cat#270733
adenosine monophosphate-^13^C_10_,^15^N_5_	MedChem Express	Cat#HY-A0181S
Trypan blue solution	Bio-Rad	Cat#1450021
OASIS HLB μElution plates	Waters Corp	Cat#186001828BA
Trypsin/LysC protease mix	Pierce	Cat#A41007
NiNTA resin	Gold Biotechnology	Cat#H-350-50
Rabbit reticulocyte lysate (RRL)	Green Hectares	N/A
0.22 μm Costar Spin-X filter	Corning	Cat#CLS8161-100EA
10 kDa cutoff centrifugal filter tubes	VWR	Cat#82031-348
Luknova SuperSep 12 g silica column	Biotage Isolera One	N/A
Critical commercial assays		
Glucose reagent	μDialysis	Cat#P000023
L-Lactate Assay Kit I	Eton Bioscience	Cat#1200012002
BCA Protein Assay Kit	Pierce	Cat#23227
Qubit RNA High Sensitivity Assay	ThermoFisher Scientific	Cat#Q32855
Deposited data		
Proteomics data related to Table S2	PRIDE repository	PXD068090
Metabolomics data related to Tables S4 and S5	MassIVE repository	MSV000099170
Metabolomics data related to Table S6	MassIVE repository	MSV000101773
Raw and uncropped western blot, gel, TLC, and autoradiograph data	Mendeley Data	https://doi.org/10.17632/2yx3sh7vmp.1
Experimental models: Cell lines		
HEK293T	American Type Culture Collection	Cat#CRL-3216; RRID: CVCL_0063
HEK293T	Orozco et al.^44^	N/A
HEK293T AMPKα1/2 DKO	Orozco et al.^44^	N/A
THP-1	American Type Culture Collection	Cat#TIB-202; RRID: CVCL_0006
Oligonucleotides		
ggcggatccCAAAACTTAGATGAGATTCTAAAGAAACTGAG	Integrated DNA Technologies	JBO372
gcctctagaCTAGCACCCATAAACAGTTCCATC	Integrated DNA Technologies	JBO373
gccgagctcCCAAAGCGATTACCTTCAGCATC	Integrated DNA Technologies	JBO381
gccggatccATAACCCTCTACCTCTTAGCATTGTC	Integrated DNA Technologies	JBO382
gccggatccAGGATAATTTGGGTTCCCATTCCC	Integrated DNA Technologies	JBO383
gcctctagaGCATCAGATGGTATAACAACTTTGG	Integrated DNA Technologies	JBO384
Recombinant DNA		
pcDNA3.1-P_CMV_::3xFLAG-GFP amp^R^ neo^R^	This paper	pJB63
pcDNA3.1-P_CMV_::3xFLAG-SidL amp^R^ neo^R^	This paper	pJB91
pcDNA3.1-P_CMV_::3xFLAG-SidL-S527A amp^R^ neo^R^	This paper	pJB92
pcDNA3.1-P_CMV_::3xFLAG-SidL-H571A amp^R^ neo^R^	This paper	pJB152
pcDNA3.1-P_CMV_::3xFLAG-SidL-E575A amp^R^ neo^R^	This paper	pJB153
pcDNA3.1-P_CMV_::3xFLAG-LnaB amp^R^ neo^R^	This paper	pJB224
pcDNA3.1-P_CMV_::3xFLAG-LegK4Δ1-58 amp^R^ neo^R^	This paper	pJB225
pcDNA3.1-P_CMV_::3xFLAG-Lpg0209 amp^R^ neo^R^	This paper	pJB226
pcDNA3.1-P_CMV_::3xFLAG-SidL-ΔN60 amp^R^ neo^R^	This paper	pJB108
pcDNA3.1-P_CMV_::3xFLAG-SidL-ΔN236 amp^R^ neo^R^	This paper	pJB110
pcDNA3.1-P_CMV_::3xFLAG-SidL-ΔC43 amp^R^ neo^R^	This paper	pJB156
pcDNA3.1-P_CMV_::3xFLAG-SidL-ΔC28 amp^R^ neo^R^	This paper	pJB157
pcDNA3.1-P_CMV_::3xFLAG-SidL-H571A-ΔN60 amp^R^ neo^R^	This paper	pJB214
pcDNA3.1-P_CMV_::3xFLAG-SidL-H571A-ΔN236 amp^R^ neo^R^	This paper	pJB215
pcDNA3.1-P_CMV_::3xFLAG-SidL-H571A-ΔC43 amp^R^ neo^R^	This paper	pJB218
pcDNA3.1-P_CMV_::3xFLAG-SidL-H571A-ΔC28 amp^R^ neo^R^	This paper	pJB219
pGEX-3C (ori, amp^R^, P_tac_::GST-3C; *E. coli* expression vector)	Cytiva	pGEX-3C
pGEX-3C::3xFLAG-GFP	This paper	pJB193
pGEX-3C::3xFLAG-SidL	This paper	pJB194
pGEX-3C::3xFLAG-SidL-H571A	This paper	pJB209
pGEX-3C::3xFLAG-LnaB	This paper	pJB213
pSR47s (R6K, *sacB*, kan^R^); *Legionella* suicide vector	Merriam et al.^97^	pSR47s
pSR47s:*lpg0437* (*sidL* deletion plasmid)	This paper	pJB251
pJB908 (RSF1010 ori, tdΔi, ΔoriT, thyA, amp^R^, P_tac_); empty vector (pEV) for *Legionella*	Sexton and Vogel^125^	pJB908
pDTI116 (pJB908-P_tac_::3xFLAG); *Legionella* expression vector	Isaac et al.^96^	pDTI116
pDTI116::P_tac_::3xFLAG-SidL	This paper	pJB245
pDTI116:: P_tac_::3xFLAG-SidL-H571A	This paper	pJB246
Software and algorithms		
CELLCYTE Studio	Cytena	N/A
Prism (versions 10 & 11)	GraphPad	https://www.graphpad.com/
R	R Core Team	https://www.r-project.org
Rstudio	Posit, PBC	https://posit.co
ggplot2	Wickham^99^	https://ggplot2.tidyverse.org/
DIA-NN 2.1.0	Demichev et al.^121^	https://github.com/vdemichev/DiaNN
DIA-Analyst	Shah et al.^122^	https://analyst-suites.org
Xcalibur 4.1.50	ThermoFisher Scientific	N/A
FreeStyle 1.3 SP2	ThermoFisher Scientific	N/A
MassHunter Acquisition Software (v12.3)	Agilent	N/A
Skyline (v25.1)	McLean et al.	https://skyline.ms/
AlphaFold 3 (AF3)	Abramson et al.^53^	https://alphafoldserver.com/
ChimeraX	Goddard et al.^117^	https://www.rbvi.ucsf.edu/chimerax/
ChemDraw	Revvity Signals Software, Inc.	N/A
Mol*	Sehnal et al. ^118^	RRID:SCR_017551
PSI-BLAST	Altshcul et al. ^100^	RRID:SCR_001010
JACKHMMER	Potter et al. ^101^	RRID:SCR_005305
MMseqs2	Steinegger et al. ^102^	RRID:SCR_022962
HHpred	Zimmermann et al. ^103^	RRID:SCR_010276
PFAM	Mistry et al. ^104^	RRID:SCR_004726
PDB	Berman et al. ^105^	RRID:SCR_012820
MAFFT	Katoh et al. ^112^	RRID:SCR_011811
FastTree	Price et al. ^114^	RRID:SCR_015501
TreeViewer	Bianchini et al. ^116^	N/A
HHalign	Steinegger et al. ^106^	RRID:SCR_016133
DaliLite	Liisa Holm^113^	RRID:SCR_013433
FOLDSEEK	van Kempen et al. ^119^	RRID:SCR_027018
HMMSCAN	Sean R. Eddy^111^	RRID: SCR_005305
RPSBLAST	Schäffer et al.^110^	N/A
Genome	NCBI resources	RRID:SCR_002474
Bruker TopSpin 4.4.1	Bruker	N/A
Other		
CELLCYTE X imager	Cytena	N/A
SW-41 swinging-bucket rotor	Beckman Coulter	Cat#331362
Type 45 Ti fixed-angle rotor	Beckman Coulter	Cat#339160
Biocomp Piston Gradient Fractionator	Biocomp	N/A
Orbitrap Exploris 480 mass spectrometer (for proteomics)	ThermoFisher Scientific	N/A
Dionex Ultimate 3000 UPLC system (for *in vitro* and HEK293T metabolite analysis)	ThermoFisher Scientific	N/A
QExactive Orbitrap mass spectrometer (for *in vitro* and HEK293T metabolite analysis)	ThermoFisher Scientific	N/A
SeQuant ZIC-pHILIC column (for *in vitro* and HEK293T metabolite analysis)	Millipore Sigma	N/A
1290 Infinity III LC coupled to an 6495D triple quadrupole mass spectrometer (for infected THP-1 metabolite analysis)	Agilent	N/A
ZORBAX RRHD Extend C18 column (for infected THP-1 metabolite analysis)	Agilent	Cat#759700-902
0.01% InfinityLab deactivator additive (v/v)	Agilent Technologies	Cat#5191-4506
Synergy H1 microplate reader	BioTek	N/A
Trans-blot Turbo Transfer System	Bio-Rad	N/A
ChemiDoc Imaging System	Bio-Rad	N/A
Odyssey CLx	LICORbio	N/A
0.5-inch diameter horn	Branson Ultrasonics	Cat#101-147-049
Akta pure chromatography system	Cytiva	N/A
NanoDrop Spectrophotometer Eight	Thermo Scientific	N/A
Model 583 Gel Dryer	Bio-Rad	N/A
Amersham TYPHOON RGB	Cytiva	N/A
phosphor screen	Cytiva	Cat#BAS-IP-MS-2040-E
Reacti-Therm III Heating/Stirring Module	Pierce	N/A
TC20 Automated Cell Counter	Bio-Rad	N/A
800 MHz Bruker Avance NEO	Bruker	N/A
TS100 inverted microscope	Nikon	N/A

## Highlights:

- SidL is an actin-dependent adenylyltransferase that targets host glycolysis.
- SidL adenylates 3PG to produce 2-AMP-3PG.
- Expression of SidL in human cells disrupts glycolysis and blocks mTORC1 signaling.
- Production of 2-AMP-3PG by SidL occurs early during the *Legionella* infection cycle.
