# Supplementary material for: The Legionella pneumophila effector SidL is an adenylyltransferase that modifies the glycolytic intermediate 3-phosphoglycerate
Source: Mol Cell. Author manuscript; Available in PMC 2026 Aug 3. (PMC13431035; doi:10.1016/j.molcel.2026.07.007)
Supplement: 1 — Document S1. Supplemental Figures S1-S9. [file NIHMS2196818-supplement-1.pdf]

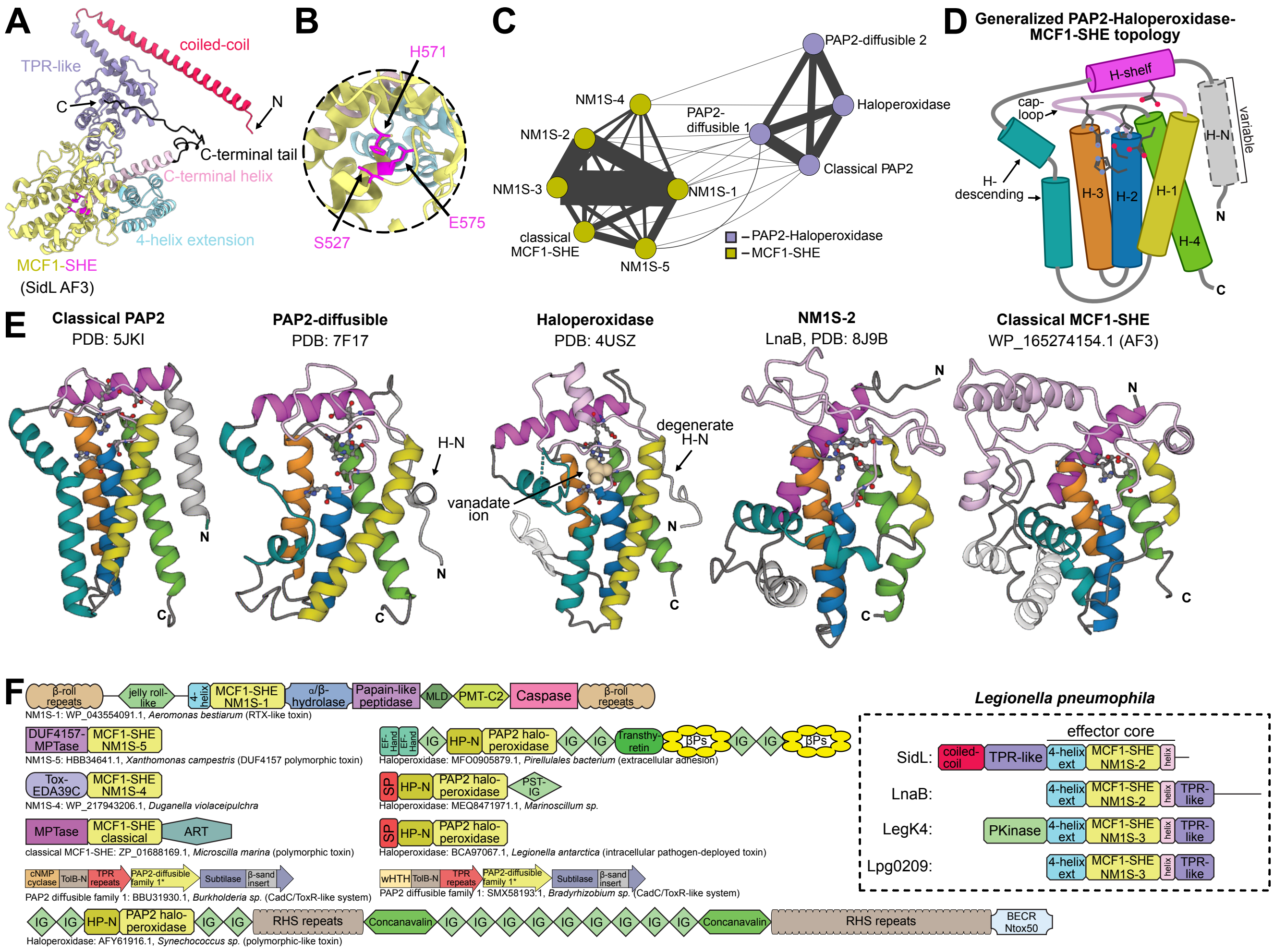

Figure S1. Structural and phylogenetics analyses of SidL and PAP2 superfamily proteins.

(A) AlphaFold3 prediction of SidL, colored by its identified regions as in **Fig. 2A**.

(B) View of the region of SidL surrounding the amino acids S527, H571, and E575 from the prediction in (A).

(C) Relationship network of the PAP2-Haloperoxidase-MCF1-SHE families. Thickness of edges is scaled by negative-log HHalign e-values (see **Table S1**). Families are colored according to the community identified by the Leiden algorithm[1] (see **STAR Methods**).

(D) Generalized topology diagram depicting the core conserved structural elements of the PAP2-Haloperoxidase-MCF1-SHE superfamily. Side chains of conserved residues are rendered as chemical structures. In addition to the core SHE residue triad, frequently well-conserved active site pocket-supporting residues are shown here and in (D) including an acidic residue in H-shelf, a basic residue in the cap-loop, and a histidine in H-2.

(E) Experimentally determined and AlphaFold3-modeled representative structures of the superfamily. Conserved residues in (C) are rendered as ball-and-stick. Structural features are colored to match (B). Poorly conserved

structural elements are colored in light gray. Primary source sequences for AlphaFold3 models are provided by gene name or NCBI accession number.

(F) Representative genome contexts for PAP2-Haloperoxidase-MCF1-SHE families with known or predicted roles in biological conflict[2] or responses to environmental stresses. Genes are depicted by box arrows, with the arrowhead indicating the 3’ end of the gene. Genes encoding proteins with multiple domains are broken into labeled sections. Domain architectures are depicted by the individual domains represented by distinct shapes. All PAP2-Haloperoxidase-MCF1-SHE domains are colored yellow for consistency. Domain architectural depictions of the MCF1-SHE-domain containing proteins from *L. pneumophila* are shown within the dashed box. Abbreviations: HP-N, Haloperoxidase N-terminal; MLD, Membrane Localization Domain; PMT-C2, *Pasteurella mutocida* toxin C2-like; MPTase, Metallopeptidase; βPs, β-propeller repeats; IG, Immunoglobulin-like; PST-IG, Porphyromonas Secretion Tail Immunoglobin-like; ART, ADP-ribosyltransferase; wHTH, winged Helix-Turn-Helix; BECR, Barnase-EndoU-Colicin E5/D-RelE-like; PKinase, Protein Kinase.

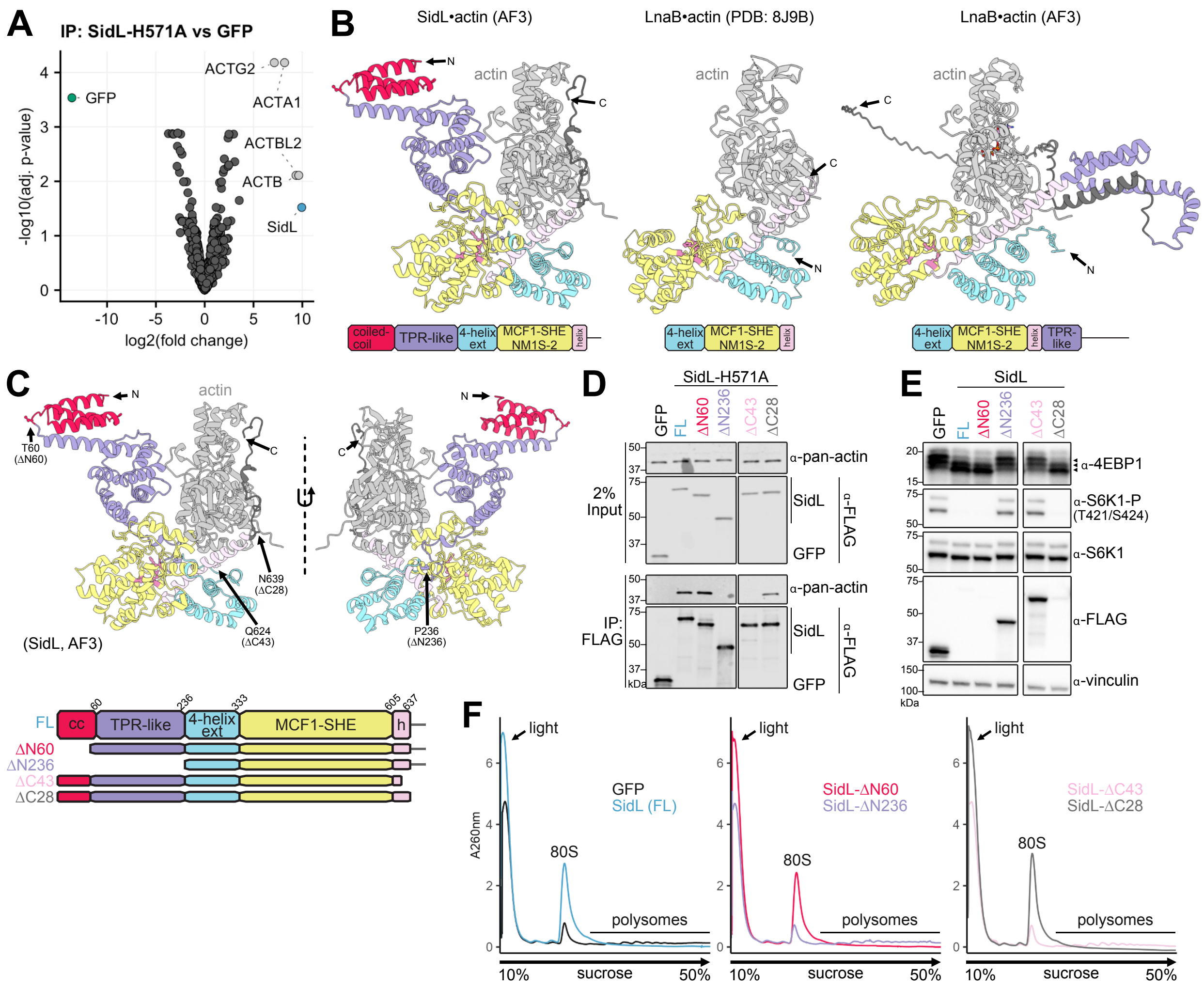

Figure S2. Actin-binding is necessary for SidL function in HEK293T cells.

(A) Anti-FLAG affinity purification of 3xFLAG-SidL-H571A (pJB152) or 3xFLAG-GFP (pJB63) from lysates of HEK293T cells transfected with plasmids encoding the indicated proteins. Volcano plot of protein enrichment in 3xFLAG-SidL-H571A compared to 3xFLAG-GFP, done in technical triplicate (n=3). Differential analysis was done using DIA-Analyst,[3] which calculates *p*-values using a moderated *t* test (Benjamini-Hochberg adjusted), with Bayesian Principal Component Analysis (BPCA) imputation applied. See **STAR Methods** for details and **Table S2**.

(B) Comparison of AlphaFold3 predictions of SidL and LnaB in complex with actin (ACTB) to an experimentally derived LnaB-actin complex (PDB 8J9B).[4] N- and C-termini are indicated by "N" and "C", respectively. The conserved SHE residues are colored in magenta and the visible domains are colored as in the diagrams below each structure. Note the TPR-like C-terminal region of LnaB is missing in PDB 8J9B.

(C) Above: AlphaFold3 prediction of SidL-actin interaction with SidL domains colored as in **Fig. 2A** and actin colored in light gray; SidL-SHE residues are colored magenta and truncation residues are shown as ball and stick. Below: Schematics of SidL truncation variants with domains indicated to scale ("CC" denotes Coiled-coil region).

(D) Anti-FLAG affinity purification of 3xFLAG-SidL-H571A (pJB152) and truncation variants (pJB214, pJB215, pJB218, and pJB219) testing for co-purification of actin from lysates of HEK293T cells transfected with plasmids encoding the listed proteins. 2% input and purification eluates were analyzed by immunoblotting with indicated antibodies. Representative of two independent experiments.

(E) Immunoblots for mTORC1 activity in HEK293T cells transfected for ~18-20 hours with plasmids encoding 3xFLAG-GFP (pJB63), wild-type 3xFLAG-SidL (pJB91), or 3xFLAG-SidL truncation variants (pJB108, pJB110, pJB156, and pJB157).

(F) Sucrose gradient traces of lysates from HEK293T cells transfected for ~18-20 hours with plasmids encoding expressing 3xFLAG-GFP (pJB63), 3xFLAG-SidL (pJB91), or 3xFLAG-SidL truncation variants (pJB108, pJB110, pJB156, and pJB157).

Panels D-F are representative of two independent experiments.

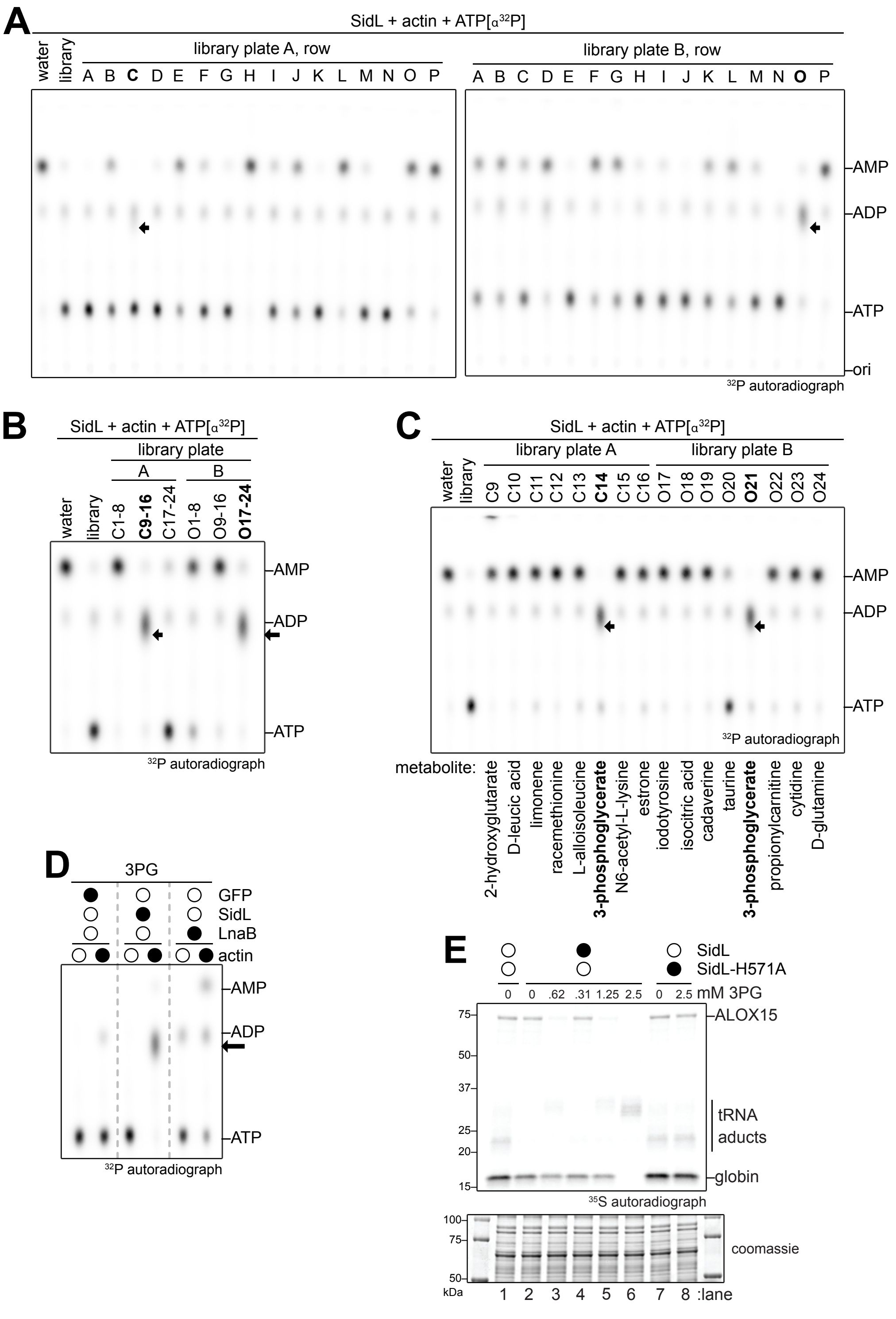

Figure S3. An *in vitro* biochemical screen identifies 3-phosphoglycerate as the substrate of SidL.

(A) TLC analysis of reactions containing 3xFLAG-SidL, actin, ATP[α^{32}P] with water, 696-metabolite library,[5] or different sub-pools of this library, based on their positions in two 384-well plates (see **Table S3**). Black arrow

indicates production of an [$\alpha^{32}P$]-modified species. TLC separation was done with 500 mM KH_2PO_4 pH 3.5 mobile phase.

(B) As in (A) but with smaller sub-pools of library metabolites.

(C) As in (A) but with individual library metabolites.

(D) *In vitro* translation reactions supplemented with methionine[^{35}S] were performed in the presence of 50 nM of recombinant SidL or SidL-H571A with the indicated 3PG supplementations. Newly translated globin and lipoxygenase (ALOX15) proteins are visualized by SDS-PAGE and autoradiography. The ~25 kDa species represent charged tRNA and tRNA-protein adducts. Total protein loading is visualized by coomassie stain. Note that lanes 3 and 4 were swapped.

(E) [$\alpha^{32}P$]-modification of 3PG by 3xFLAG-SidL compared to 3xFLAG-GFP, 3xFLAG-SidL-H571A, and GST-3C-3xFLAG-LnaB ± actin, as analyzed by TLC with 500 mM KH_2PO_4 pH 3.5 mobile phase.

For panels D and E, closed and open circles respectively indicate the presence and absence of the annotated recombinant proteins.

Panels A-C are of single experiments and panels D-E are representative of two independent experiments.

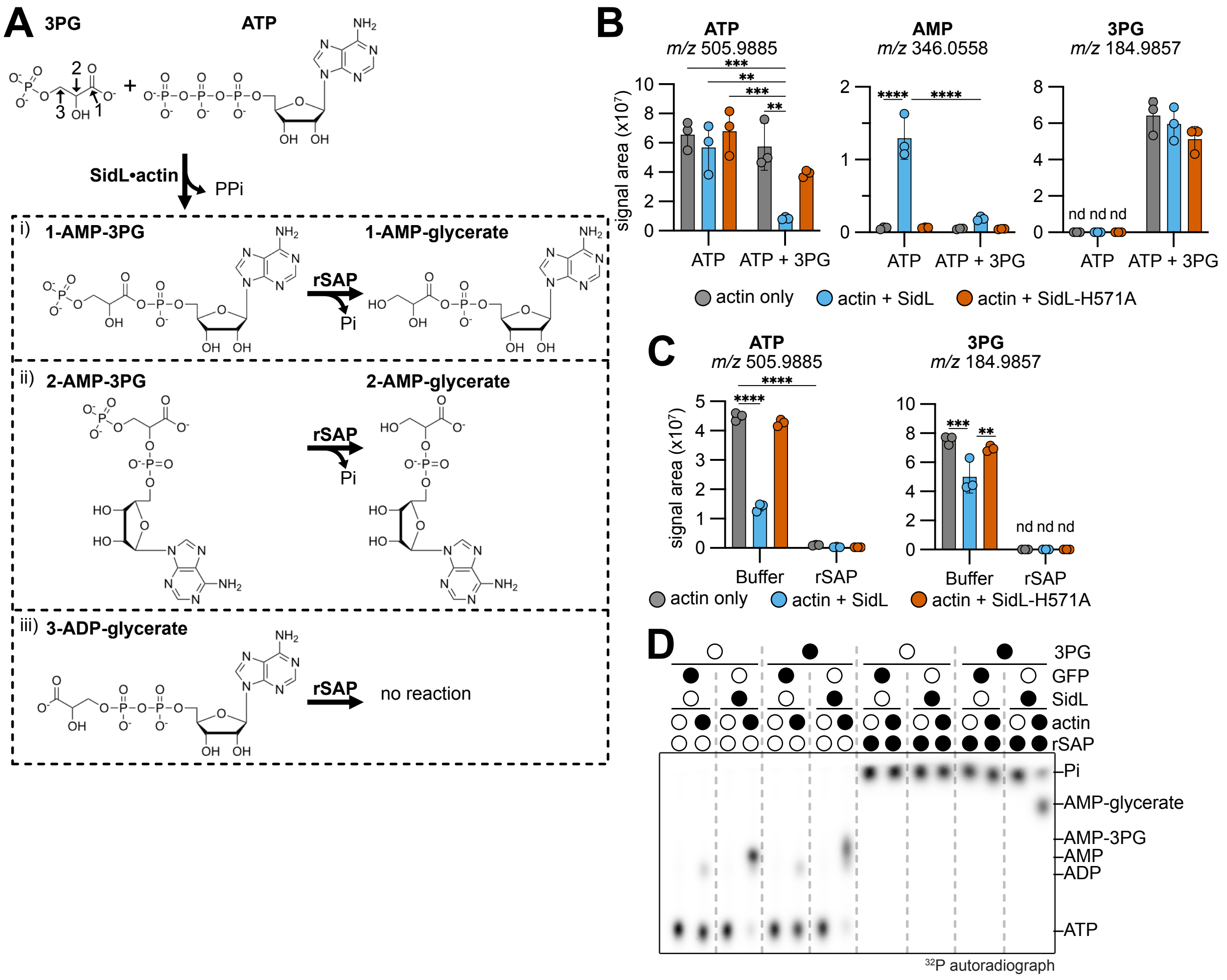

Figure S4. AMP-3-phosphoglycerate susceptibility to rSAP treatment.

(A) Molecular depiction of potential SidL products and the expected effect of rSAP treatment on these potential products; the carbons of 3PG are numbered.

(B, C) LC-MS signal intensities for ATP, AMP, and 3PG from *in vitro* reactions containing ATP and actin alone or with 3xFLAG-SidL or 3xFLAG-SidL-H571A with or without 3PG. For **(C)**, the reactions were first incubated then split in half and subsequently treated with rSAP or its storage buffer and the levels of ATP and 3PG were analyzed as a positive control to validate rSAP activity. *m/z* for each metabolite is indicated. Bars are mean ± standard deviation with points representing individual reactions (n=3). Two-way ANOVA with multiple comparisons: $p \leq 0.05$ (*), $p \leq 0.01$ (**), $p \leq 0.001$ (***), $p \leq 0.0001$ (****). Only relevant comparisons are shown. "nd" denotes not detected (see **Figs. 4E-F** and **Table S4**).

(D) Sensitivity of [α^{32}P]-modified 3PG to rSAP-treatment as determined by first incubating 3xFLAG-GFP or 3xFLAG-SidL with ATP[α^{32}P] ± 3PG ± actin. Reactions were then split and treated with rSAP or storage buffer prior to analysis by TLC (750 mM KH_2PO_4 pH 3.5 mobile phase). Representative of a single experiment. Closed and open circles respectively indicate the presence and absence of 3PG or the indicated proteins.

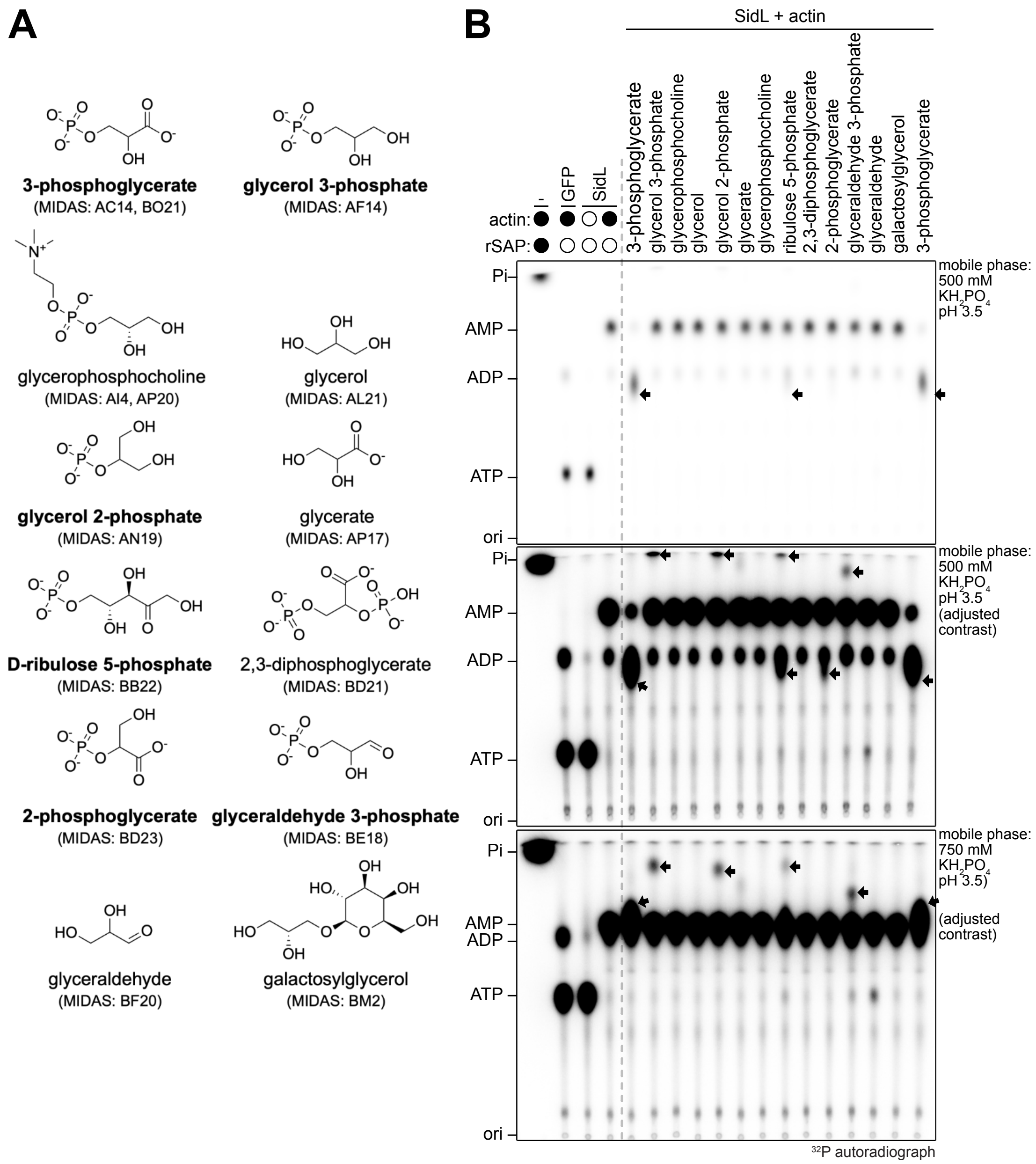

Figure S5. Testing SidL modification of metabolites with structural similarities to 3-phosphoglycerate.

(A) Molecular depiction of metabolites in the library that share similarities with 3PG; bold text denotes molecules were modified by 3xFLAG-SidL *in vitro* in (B).

(B) SidL-catalyzed modification of metabolites that share similarity with 3PG was accessed by incubating the indicated metabolites with 3xFLAG-SidL, actin, and ATP[α^{32}P] and compared to control reactions, as analyzed by TLC with mobile phases 500 mM KH_2PO_4 pH 3.5 (upper and middle panels) and 750 mM KH_2PO_4 pH 3.5 (lower panel). The contrast of the middle and lower panels was increased to visualize signal for weakly modified metabolites. Black arrows indicate production of [α^{32}P]-modified SidL products. Closed and open circles respectively indicate the presence and absence of the indicated proteins. Representative of two independent experiments.

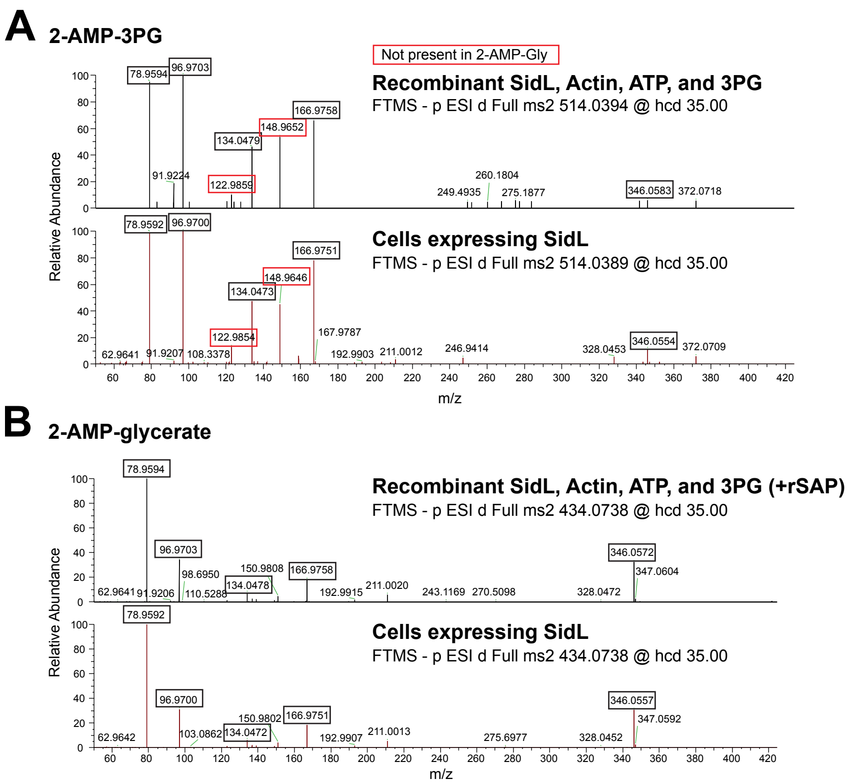

Figure S6. MS2 spectra of 2-AMP-3-phosphoglycerate and 2-AMP-glycerate.

(A, B) MS2 spectra of 2-AMP-3PG **(A)** and 2-AMP-glycerate **(B)** hydrogen-loss ions detected in *in vitro* enzyme reactions (top) and in HEK293T cells transfected with a plasmid encoding 3xFLAG-SidL (bottom; pJB91). Fragments in red are specific to 2-AMP-3PG but not 2-AMP-glycerate.

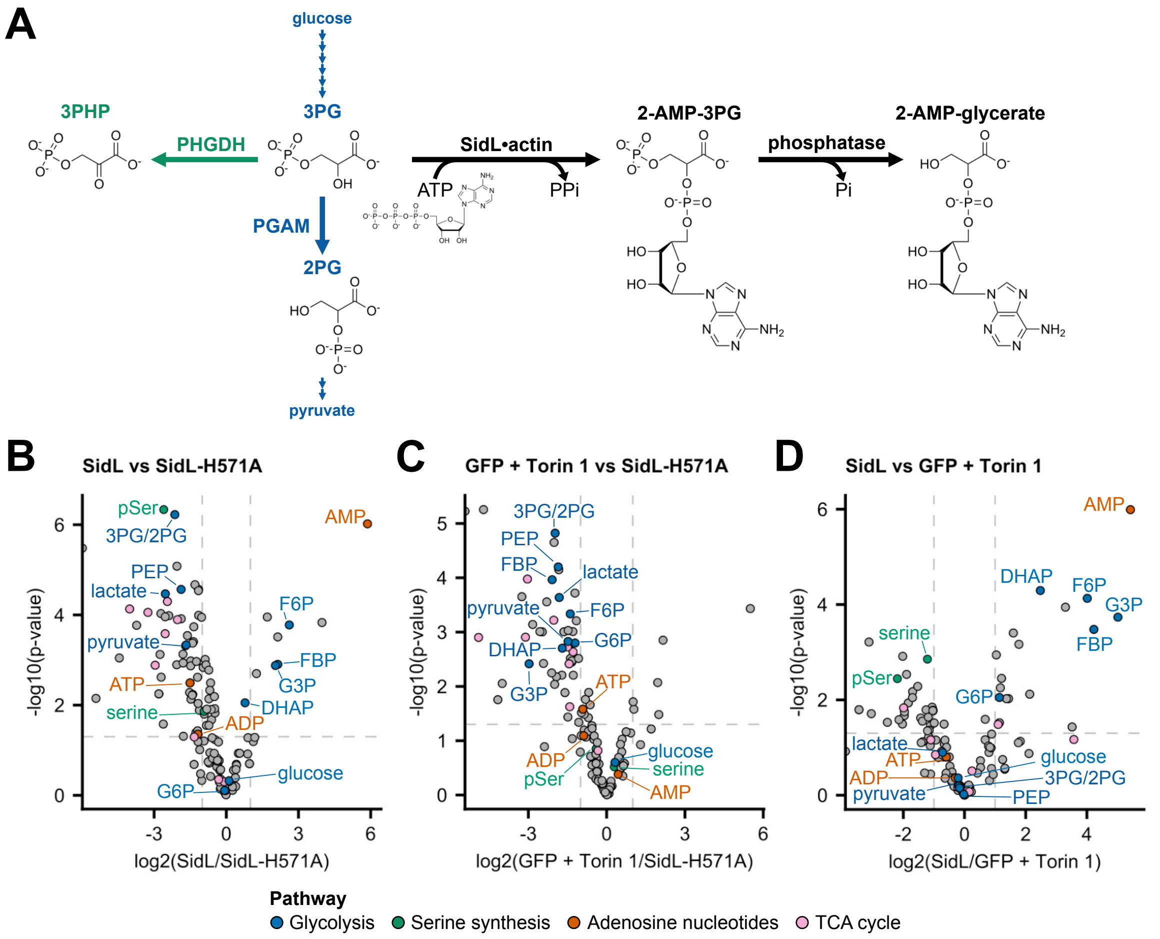

Figure S7. Effects of SidL and Torin 1 on the abundances of metabolites in HEK293T cells.

(A) Molecular representation of how 2-AMP-3PG is incompatible with proximal eukaryotic metabolism. SidL adenylates the glycolytic intermediate 3-phosphoglycerate (3PG) at its 2-position hydroxyl group, producing of 2-AMP-3PG which can be dephosphorylated by an unknown phosphatase. AMP at the 2-position of 3PG should make it incompatible with further glycolysis and in the first step of *de novo* serine synthesis. Phosphoglycerate mutase (PGAM) converts 3PG into 2-phosphoglycerate (2PG) by phosphorylating 3PG at its 2-position hydroxyl group then removing the 3-position phosphoryl group (EC5.4.2.11).[6] In serine synthesis, phosphoglycerate dehydrogenase (PHGDH) converts 3PG into 3-phosphohydroxypyruvate (3PHP) by oxidizing the 2-position hydroxyl group into a carbonyl group (EC1.1.1.95).[6]

(B) Volcano plots for changes in steady-state metabolite abundances from HEK293T cells transfected with a plasmid encoding 3xFLAG-SidL (pJB91) relative to cells transfected with a plasmid encoding 3xFLAG-SidL-H571A (pJB152) as detected by untargeted metabolomics. Cells were transfected with the plasmids for 18 hours and Torin 1 treatment started 2 hours post transfection. Changes are shown as log2-transformed fold changes and a -log10-transformed *p*-value calculated with a two-sided Student's *t* test (n=5). The dashed lines on the y- and x-axes delineate -log10(0.05) *p*-value and log2 fold change of ± 1, respectively. Selected metabolites are colored according

to pathway: glycolysis (blue), TCA cycle (pink), serine synthesis (green), adenosine nucleotides (orange). Abbreviations are as in (C).

(C) As in (B) but comparing cells transfected with a plasmid encoding 3xFLAG-GFP (pJB63) + 300 nM Torin 1 or 3xFLAG-SidL-H571A (pJB152).

(D) As in (B) but comparing cells transfected with a plasmid encoding 3xFLAG-SidL (pJB91) or 3xFLAG-GFP (pJB63) + 300 nM Torin 1.

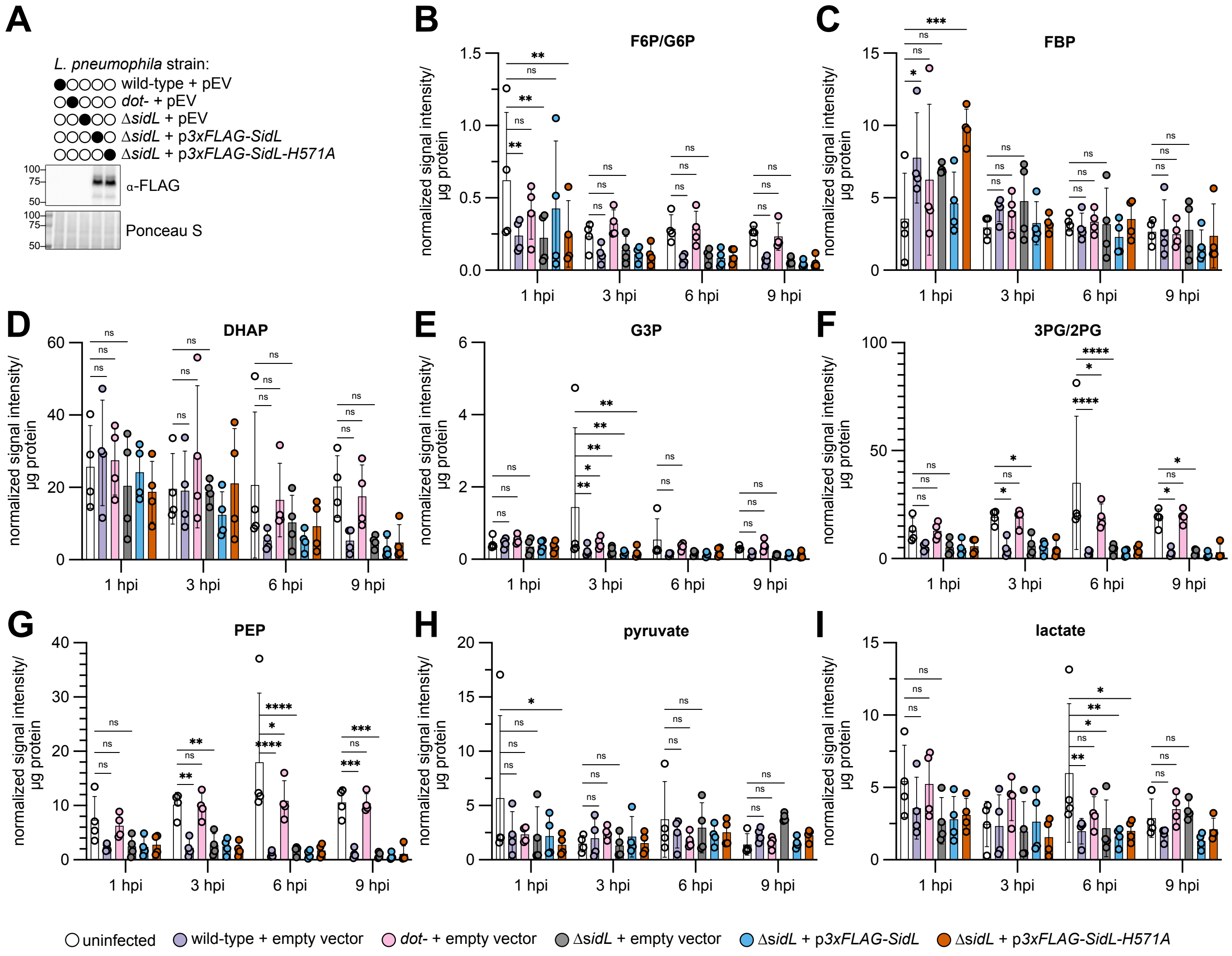

Figure S8. Effect of *L. pneumophila* infection on glycolytic intermediates in THP-1 cells.

(A) Expression of 3xFLAG-SidL and 3xFLAG-SidL-H571A in *L. pneumophila*. Western analysis of whole cell lysates of wild-type, *dot-*, or Δ*sidL L. pneumophila* strains harboring empty vector or a vector encoding an IPTG inducible 3xFLAG-SidL (p*3xFLAG-SidL*) or 3xFLAG-SidL-H571A (p*3xFLAG-SidL-H571A*). Cell cultures were incubated with 250 μM IPTG for ~2 hours to induce expression of 3xFLAG-SidL prior to harvesting. Lysates were normalized by bacteria number and Ponceau S membrane staining was used as a loading control. Closed and open circles respectively indicate the presence and absence of the indicated *L. pneumophila* strains. Blots are representative of four biological replicates.

(B-I) LC-MS detection of glucose 6-phosphate/fructose 6-phosphate (G6P/F6P) **(B)**, fructose 1,6-bisphophate (FBP) **(C)**, dihydroxyacetone phosphate (DHAP) **(D)**, glyceraldehyde 3-phosphate (G3P) **(E)**, isobaric 3-phosphoglycerate/2-phosphoglycerate (3PG/2PG) **(F)**, phosphoenolpyruvate (PEP) **(G)**, pyruvate **(H)**, and lactate **(I)** in either uninfected THP-1 cells or cells with the indicated *L. pneumophila* strains at the indicated hours post infection (hpi). Signals are normalized to a heavy AMP standard and total cellular protein content. Bars are mean ±

SD with points representing biological replicates (n=4) and colored by condition: uninfected (white), wild-type + pEV (purple), *dot-* + pEV (pink), ∆*sidL* + pEV (gray), ∆*sidL* + p*3xFLAG-sidL* (blue), ∆*sidL* + *p3xFLAG-sidL-H571A* (orange). Two-way ANOVA with multiple comparisons: not significant (ns), $p \leq 0.05$ (*), $p \leq 0.01$ (**), $p \leq 0.001$ (***), $p \leq 0.001$ (****). See **Table S6**.

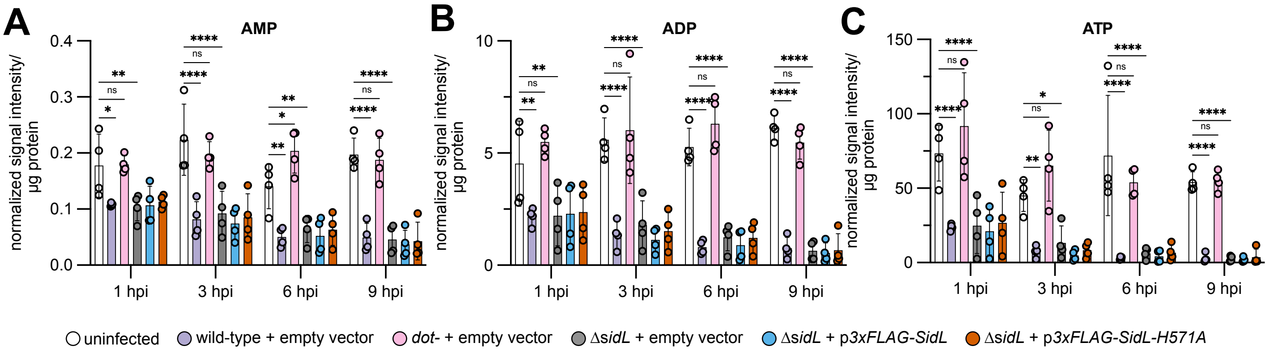

Figure S9. Effect of *L. pneumophila* infection on adenosine nucleotides in THP-1 cells.

(A-C) LC-MS detection of AMP **(A)**, ADP **(B)**, and ATP **(C)**, in either uninfected THP-1 cells or cells with the indicated *L. pneumophila* strains at the indicated hours post infection (hpi). Signals are normalized to a heavy AMP standard and total cellular protein content. Bars are mean ± SD with points representing biological replicates (n=4) and colored by condition: uninfected (white), wild-type + pEV (purple), *dot-* + pEV (pink), Δ*sidL* + pEV (gray), Δ*sidL* + p*3xFLAG-sidL* (blue), Δ*sidL* + *p3xFLAG-sidL-H571A* (orange). Two-way ANOVA with multiple comparisons: not significant (ns), $p \leq 0.05$ (*), $p \leq 0.01$ (**), $p \leq 0.001$ (***), $p \leq 0.001$ (****). See **Table S6**.

Supplemental References:

1. Traag, V.A., Waltman, L., and van Eck, N.J. (2019). From Louvain to Leiden: guaranteeing well-connected communities. Sci Rep *9*, 5233. https://doi.org/10.1038/s41598-019-41695-z.

2. Aravind, L., Iyer, L.M., and Burroughs, A.M. (2022). Discovering Biological Conflict Systems Through Genome Analysis: Evolutionary Principles and Biochemical Novelty. Annu Rev Biomed Data Sci *5*, 367–391. https://doi.org/10.1146/annurev-biodatasci-122220-101119.

3. Shah, A.D., Goode, R.J.A., Huang, C., Powell, D.R., and Schittenhelm, R.B. (2020). LFQ-Analyst: An Easy-To-Use Interactive Web Platform To Analyze and Visualize Label-Free Proteomics Data Preprocessed with MaxQuant. J Proteome Res *19*, 204–211. https://doi.org/10.1021/acs.jproteome.9b00496.

4. Fu, J., Li, S., Guan, H., Li, C., Zhao, Y.-B., Chen, T.-T., Xian, W., Zhang, Z., Liu, Y., Guan, Q., et al. (2024). Legionella maintains host cell ubiquitin homeostasis by effectors with unique catalytic mechanisms. Nat Commun *15*, 5953. https://doi.org/10.1038/s41467-024-50311-2.

5. Hicks, K.G., Cluntun, A.A., Schubert, H.L., Hackett, S.R., Berg, J.A., Leonard, P.G., Ajalla Aleixo, M.A., Zhou, Y., Bott, A.J., Salvatore, S.R., et al. (2023). Protein-metabolite interactomics of carbohydrate metabolism reveal regulation of lactate dehydrogenase. Science *379*, 996–1003. https://doi.org/10.1126/science.abm3452.

6. Hauenstein, J., Jeske, L., Jäde, A., Krull, M., Dümmer, K., Koblitz, J., Tietz, A., Jahn, D., Reimer, L.C., and Bunk, B. (2026). BRENDA in 2026: a Global Core Biodata Resource for functional enzyme and metabolic data within the DSMZ Digital Diversity. Nucleic Acids Res *54*, D527–D534. https://doi.org/10.1093/nar/gkaf1113.
