# Supplementary material for: The Legionella pneumophila effector SidL is an adenylyltransferase that modifies the glycolytic intermediate 3-phosphoglycerate
Source: Mol Cell. Author manuscript; Available in PMC 2026 Aug 3. (PMC13431035; doi:10.1016/j.molcel.2026.07.007)
Supplement: 3 — Table S2. Proteomic composition of 3xFLAG-SidL-H571A and 3xFLAG-GFP affinity purifications. Related to Fig. S2A. [file NIHMS2196818-supplement-3.pdf]

Supplemental Data S2: 2-AMP-3PG NMR Appendix

The *Legionella pneumophila* effector SidL is an adenylyltransferase that modifies the glycolytic intermediate 3-phosphoglycerate

Joshua J. Black[1,2], A. Maxwell Burroughs[3], Edrees H. Rashan[4,5], Charles R. Nosal[6,7], Katerina A. Romanov[1], Marco A. Catipovic[1,2], Catherine Valadez[1], L. Aravind[3], Kevin G. Hicks[8], Caren L. Freel Meyers[6,7], Tamara J. O'Connor[1], Matthew G. Vander Heiden[4,5,9], Rachel Green[1,2]*

(1) Department of Molecular Biology & Genetics, Johns Hopkins University School of Medicine; Baltimore, MD, USA
(2) Howard Hughes Medical Institute; Chevy Chase, MD, USA
(3) Computational Biology Branch, Division of Intramural Research, National Library of Medicine, National Institutes of Health; Bethesda, MD, USA
(4) Koch Institute for Integrative Cancer Research, Massachusetts Institute of Technology; Cambridge, MA, USA
(5) Department of Biology, Massachusetts Institute of Technology; Cambridge, MA, USA
(6) Department of Pharmacology and Molecular Sciences, Johns Hopkins University School of Medicine, Baltimore, MD, USA
(7) Chemistry-Biology Interface Graduate Training Program, Johns Hopkins University, Baltimore, MD, USA
(8) Department of Nutrition & Integrative Physiology, University of Utah College of Health; Salt Lake City, UT, USA
(9) Dana-Farber Cancer Institute; Boston, MA, USA
*Corresponding author. Email: ragreen@jhmi.edu

Table of Contents

Figure A1. 1D ^{1}H-NMR (500 MHz, D2O) of 2-AMP-3PG in reaction buffer S2

Figure A2. 1D ^{1}H-NMR (500 MHz, D2O) of 2-AMP-3PG in reaction buffer S3

Figure A3. 2D TOCSY (800 MHz, D2O) of 2-AMP-3PG post flash chromatography S4

Figure A4. 2D TOCSY (800 MHz, D2O) of 2-AMP-3PG post flash chromatography S4

Figure A5. 1D ^{13}C-NMR (200 MHz, D2O) of 2-AMP-3PG post flash chromatography S5

Figure A6. 2D ^{1}H-^{13}C HSQC (800 MHz, D_2O) of 2-AMP-3PG post flash chromatography S6

Figure A7. 2D ^{1}H-^{13}C HSQC (800 MHz, D_2O) of 2-AMP-3PG post flash chromatography S7

Figure A8. 1D ^{31}P-NMR (500 MHz, D2O) of 2-AMP-3PG in reaction buffer referenced to TPPO S8

Figure A9. 2D ^{1}H-^{31}P HMQC (500 MHz, D2O) of 2-AMP-3PG referenced to TPPO S9

References S10

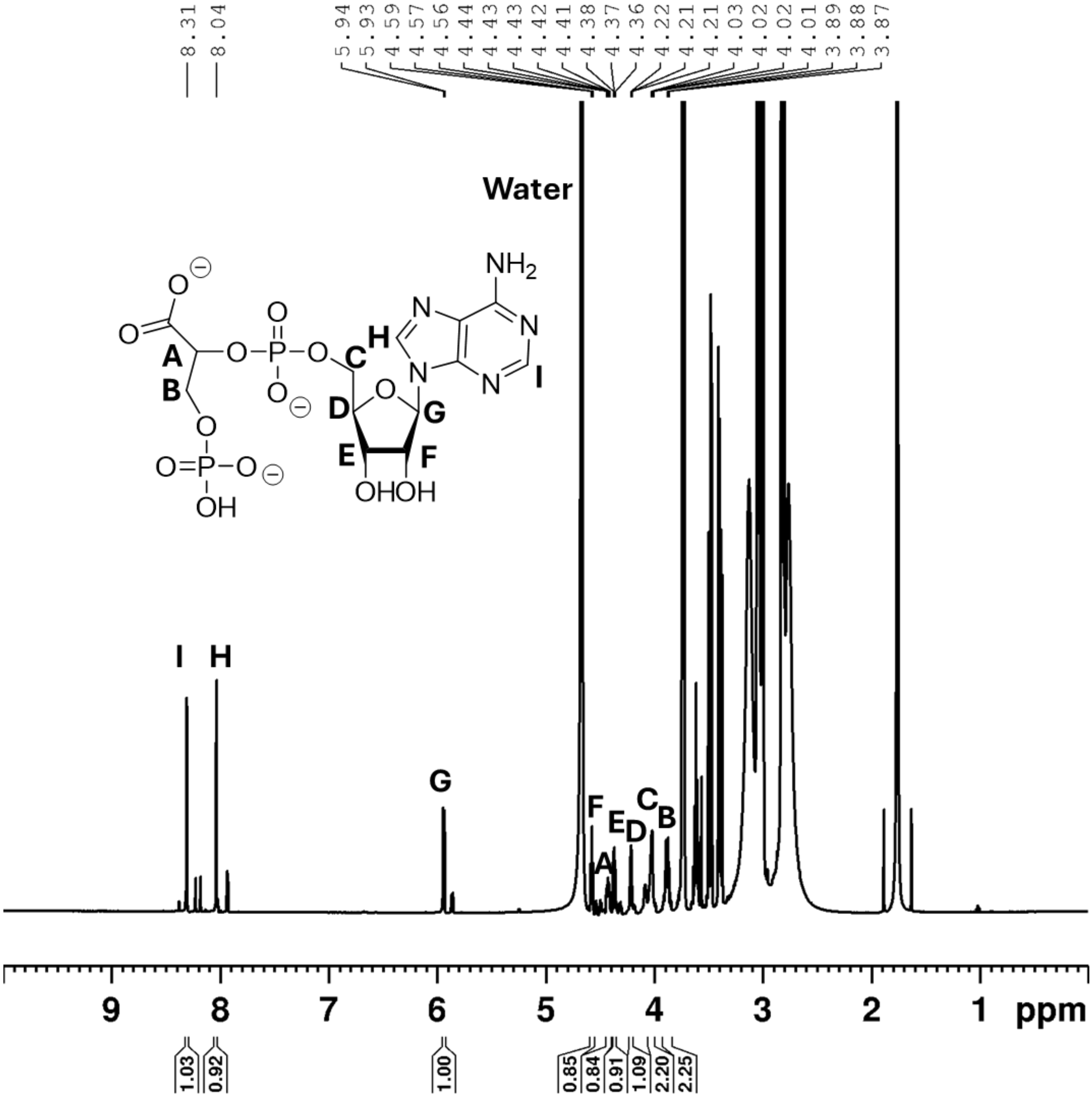

Fig A1. 1D ^{1}H-NMR (500 MHz, D2O) of 2-AMP-3PG in reaction buffer. Spectra were collected with an acquisition time of 2 s and relaxation delay of 4 s. 64 scans were collected per FID.

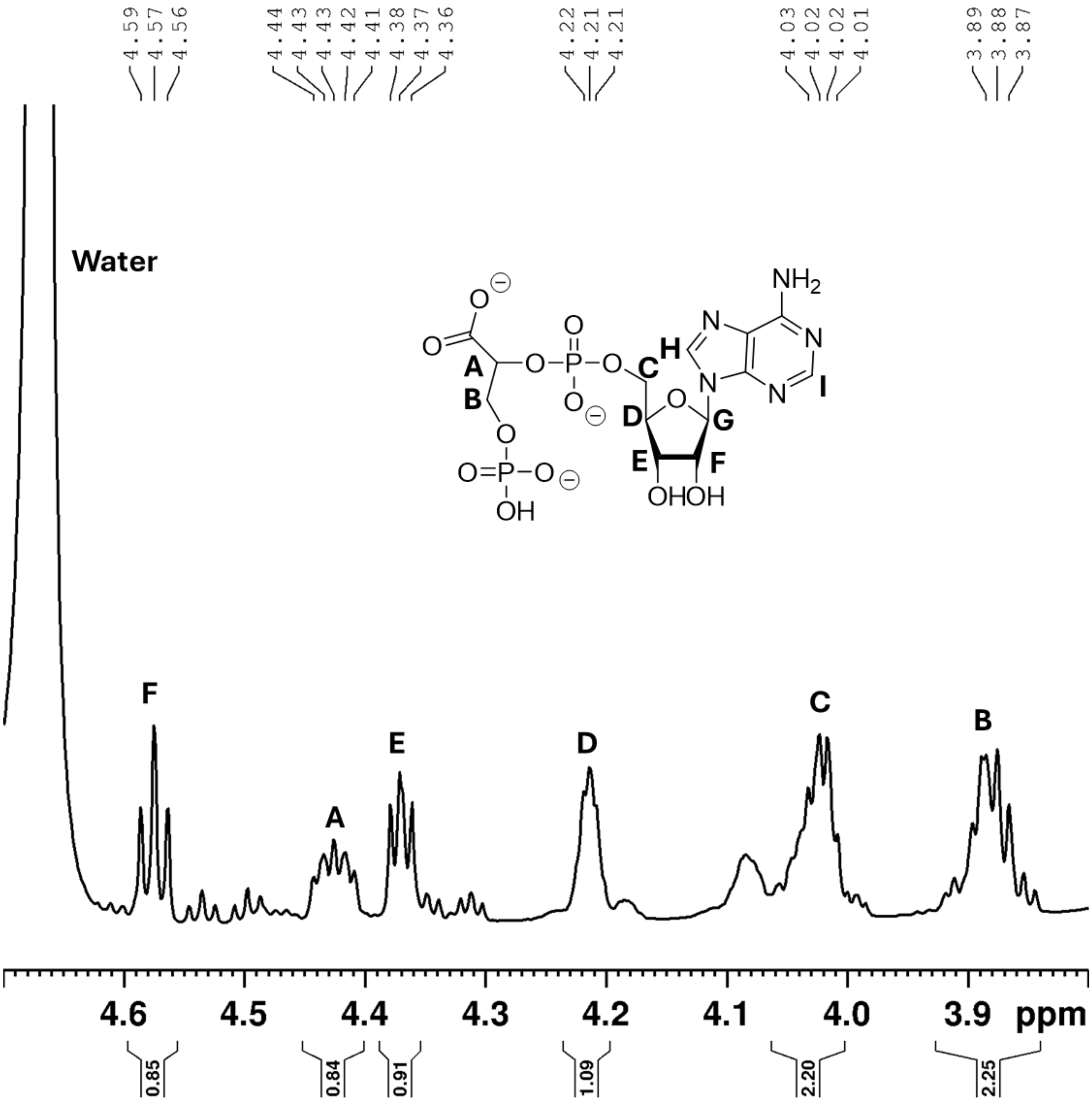

Fig A2. 1D ^{1}H-NMR (500 MHz, D2O) of 2-AMP-3PG in reaction buffer. Amplification of spectra to show protons A-F. Spectra were collected with an acquisition time of 2 s and relaxation delay of 4 s. 64 scans were collected per FID.

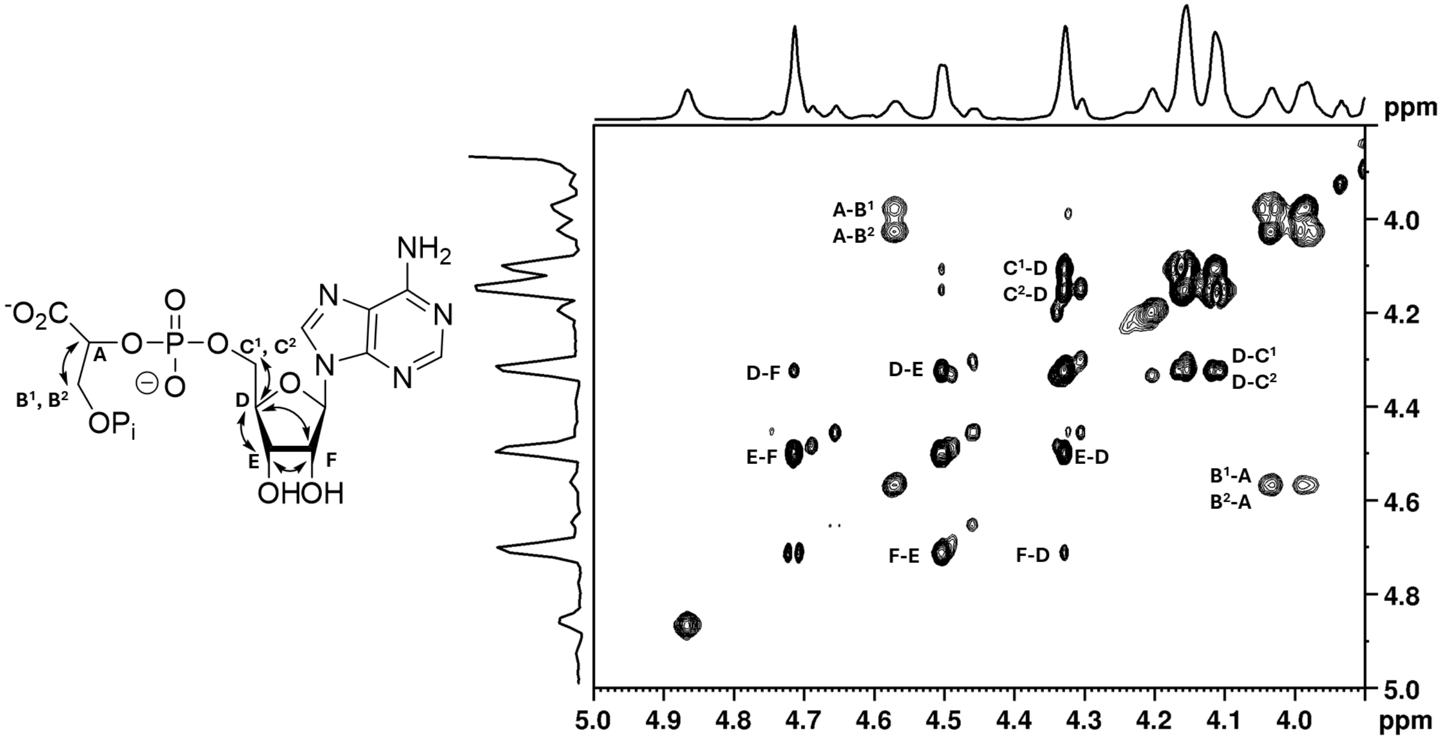

Fig A3. 2D TOCSY (800 MHz, D2O) of 2-AMP-3PG post flash chromatography. Amplification of spectra to show protons A-F. A 250 ms acquisition time (t_2) and 1 second relaxation delay with 8 scans and an F2 ^1H spectral width of 15.621 ppm and an F1 ^1H spectral width of 11 ppm centered at 4.87 ppm at 15 ℃ were used.

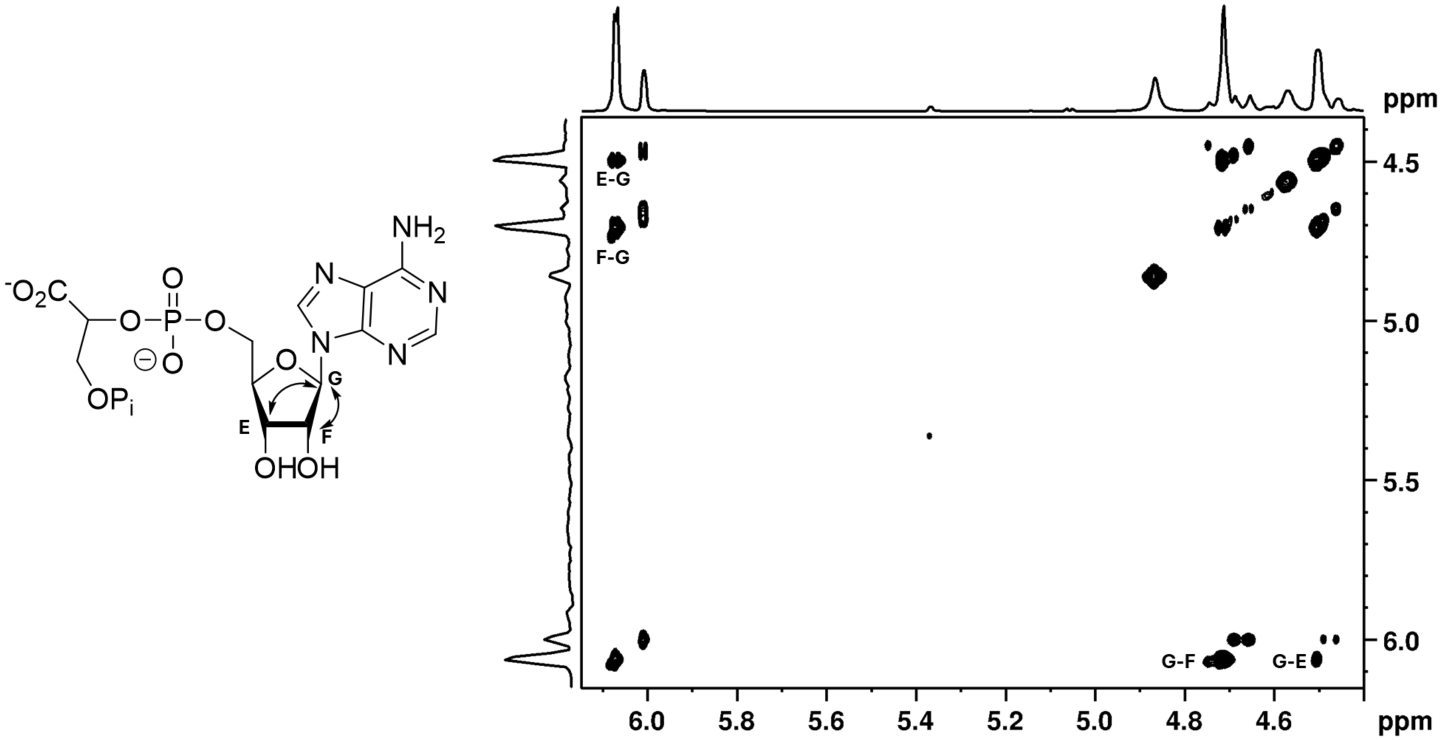

Fig A4. 2D TOCSY (800 MHz, D2O) of 2-AMP-3PG post flash chromatography. Amplification of spectra to show protons E-G. A 250 ms acquisition time (t_2) and 1 second relaxation delay with 8 scans and an F2 ^1H spectral width of 15.621 ppm and an F1 ^1H spectral width of 11 ppm centered at 4.87 ppm at 15 ℃ were used.

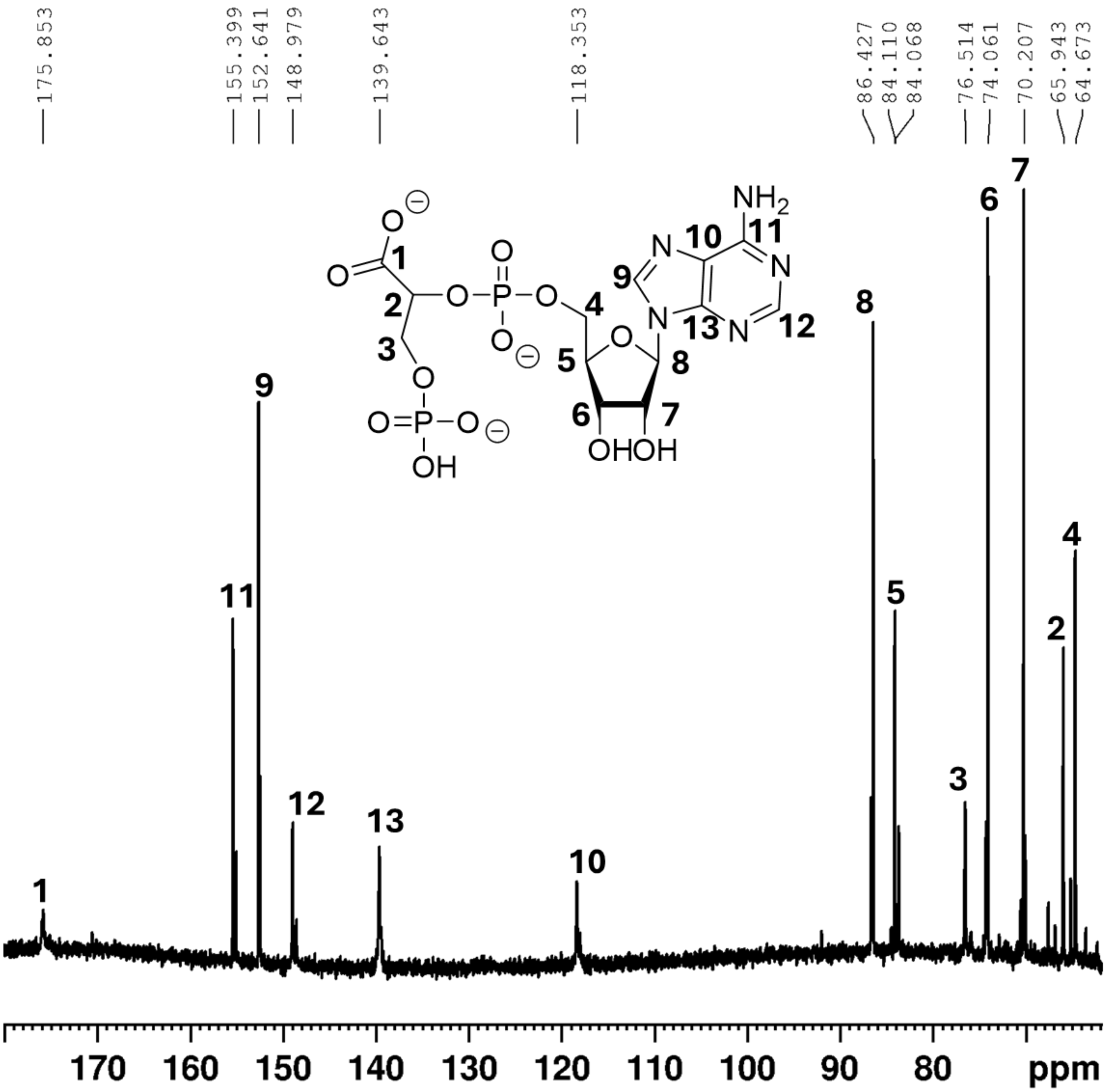

Fig A5. 1D ^{13}C-NMR (200 MHz, D2O) of 2-AMP-3PG post flash chromatography. Acquisition time was 200 ms, and a ^{1}H-^{13}C NOE was applied during a relaxation delay of 3 seconds to enhance the intensities of CH_n (n = 1, 2, 3) ^{13}C nuclei., Optimum signal to noise was obtained as follows: 1024 scans employing a 75° excitation pulse per scan were collected per FID, followed by a 20 second delay to facilitate complete return to equilibrium. 5 FIDs were added together for a total of 5120 scans. Carbon assignments for 10, 11, and 13, were made based on reported ^{13}C chemical shifts of adenosine.[1]

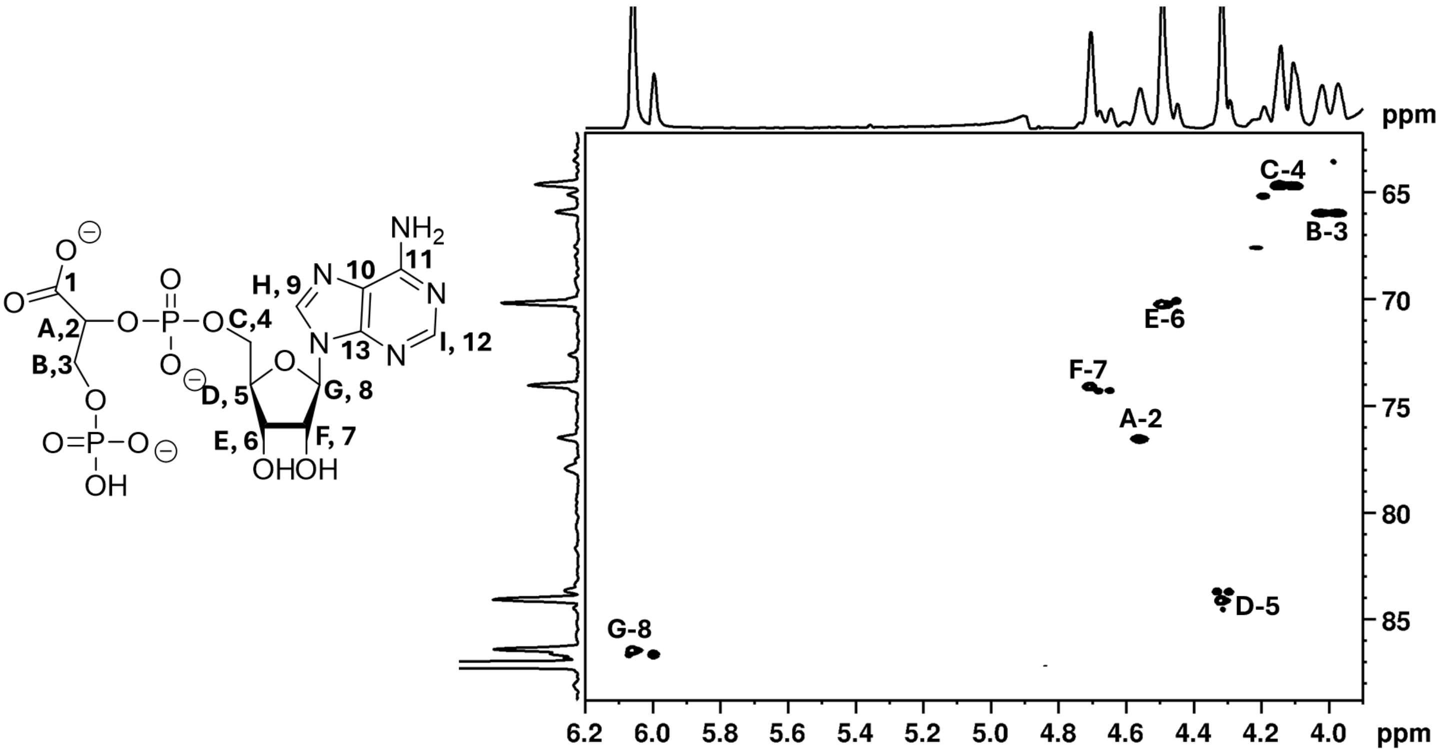

Fig A6. 2D ^{1}H-^{13}C HSQC (800 MHz, D_2O) of 2-AMP-3PG post flash chromatography. Protons (F2, direct axis) are labeled as letters and carbons (F1, indirect axis) are labeled as numbers. In the direct dimension (t_2, ^{1}H), the ^{1}H carrier was positioned at the H2O frequency (4.87 ppm at 15 ℃) with a spectral width of 15.621 ppm. 16 scans/FID, 125 ms acquisition time and a relaxation delay of 1.5 s were employed. In the indirect dimension (t_1, ^{13}C) the ^{13}C carrier was positioned at 80 ppm with a spectral width of 40 ppm, along with acquisition times of 25 ms.

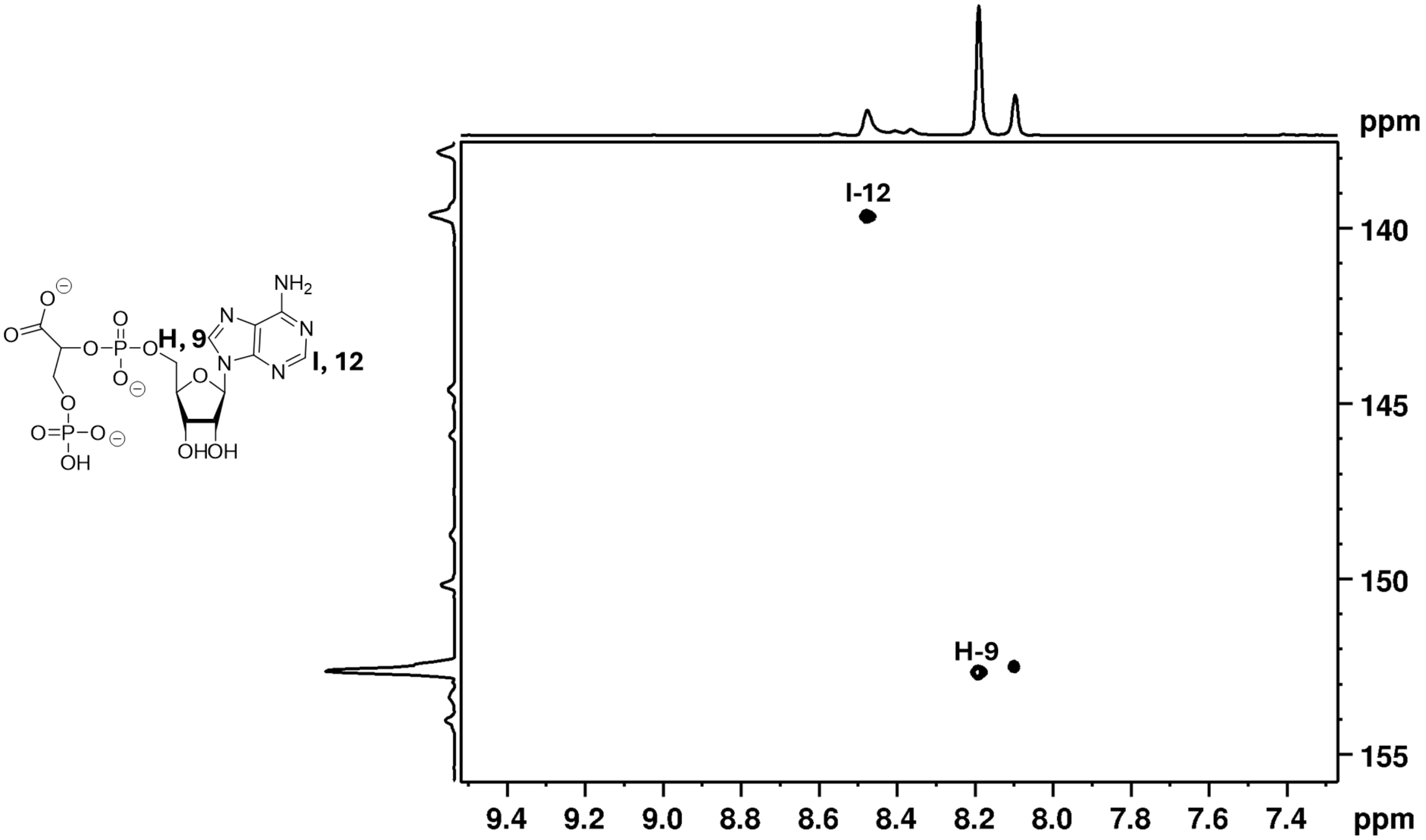

Fig A7. 2D ^{1}H-^{13}C HSQC (800 MHz, D_2O) of 2-AMP-3PG post flash chromatography. Protons (F2, direct axis) are labeled as letters and carbons (F1, indirect axis) are labeled as numbers. In the direct dimension (t_2, ^{1}H), the ^{1}H carrier was positioned at the H2O frequency (4.87 ppm at 15 ℃) with a spectral width of 15.621 ppm. 16 scans/FID, 125 ms acquisition time and a relaxation delay of 1.5 s were employed. In the indirect dimension (t_1, ^{13}C) the ^{13}C carrier was positioned at 140 ppm with a spectral width of 40 ppm, along with acquisition times of 25 ms.

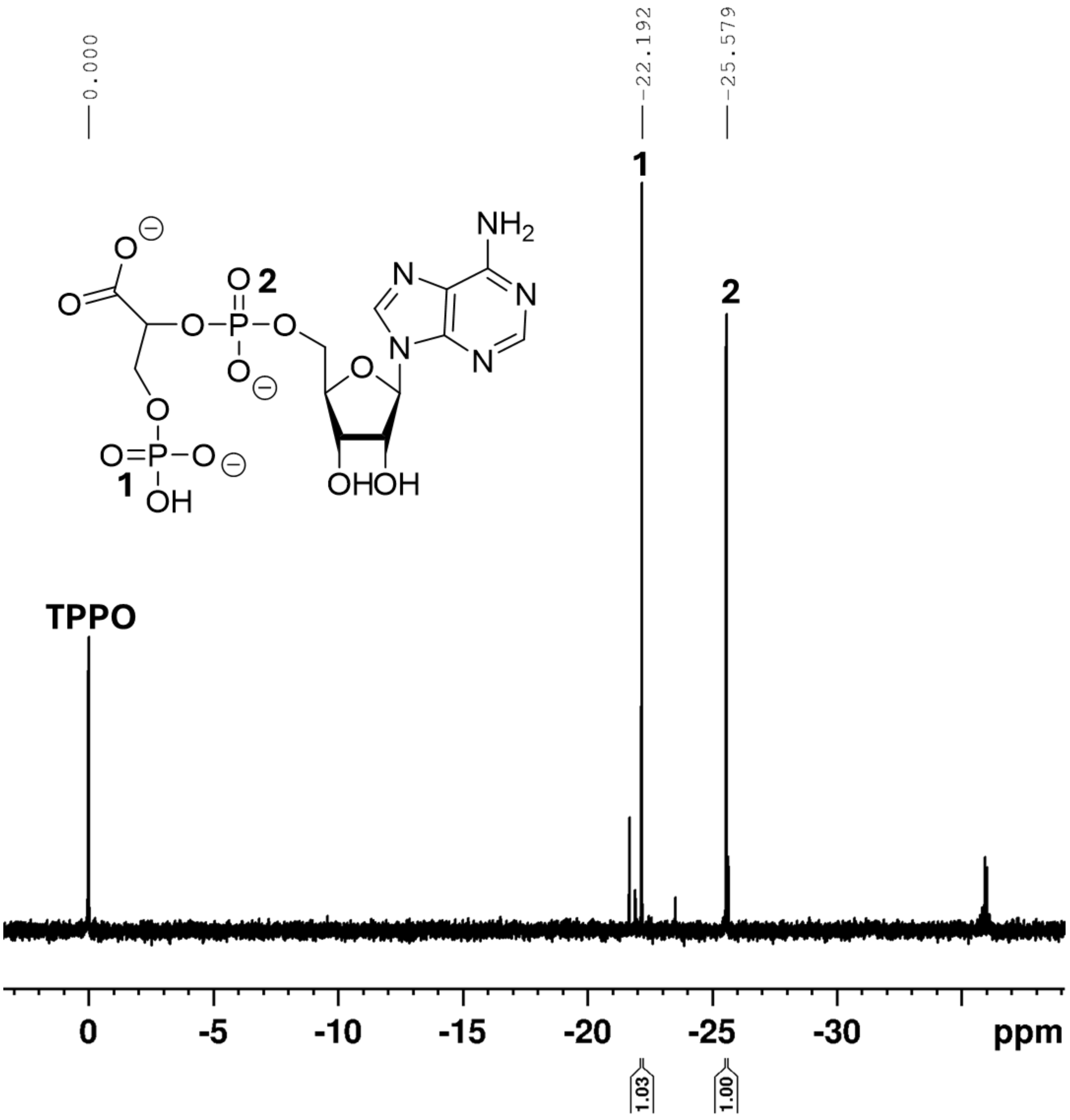

Fig A8. 1D ^{31}P-NMR (500 MHz, D2O) of 2-AMP-3PG in reaction buffer referenced to TPPO. Acquisition time was 1.5 s, relaxation delay was 2 s, and 64 scans were collected per FID.

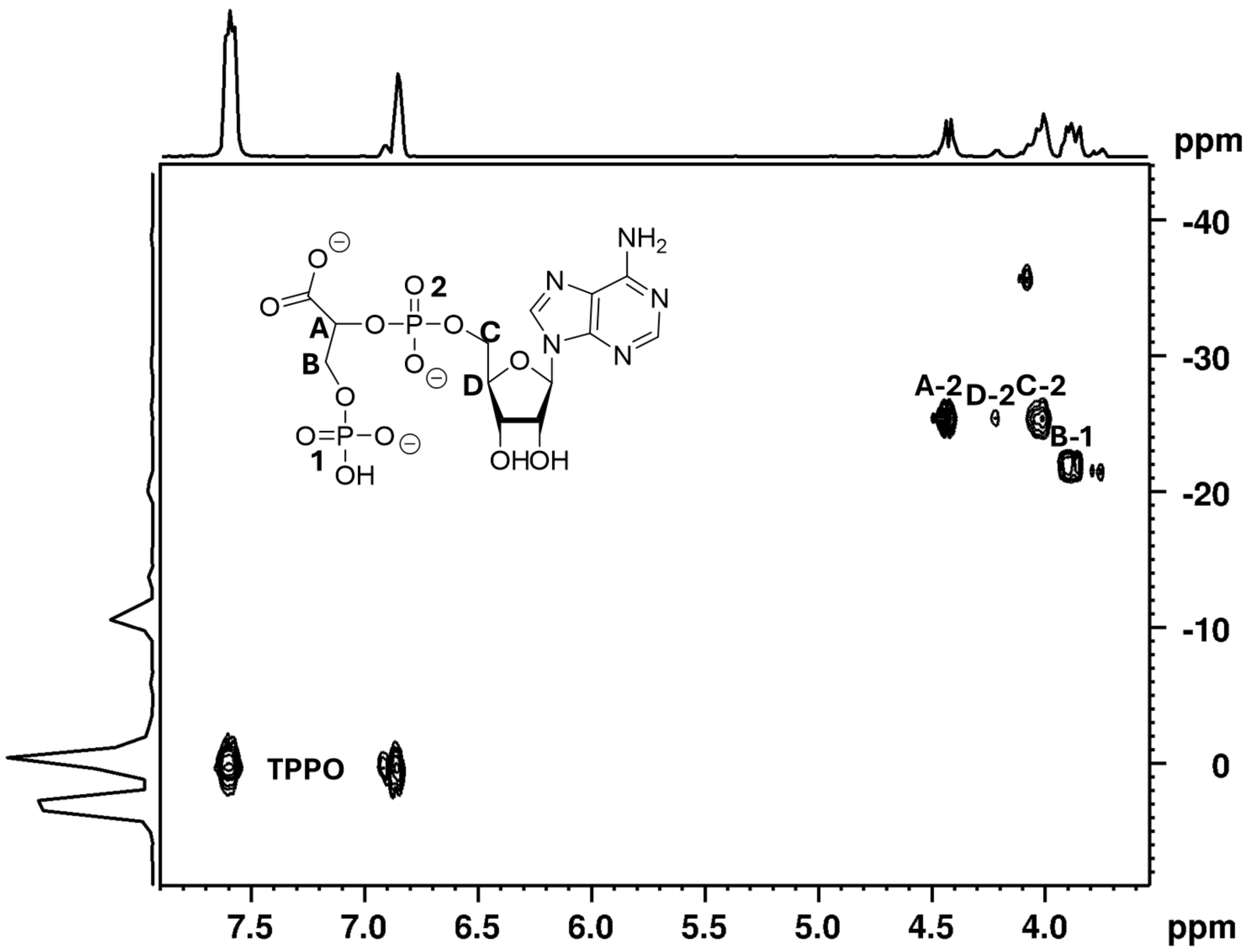

Fig A9. 2D ^{1}H-^{31}P HMQC (500 MHz, D2O) of 2-AMP-3PG referenced to TPPO. Protons (F2, direct axis) are labeled as letters and phosphorous (F1, indirect axis) are labeled as numbers. In the direct dimension (t_2, ^{1}H), the ^{1}H carrier was positioned at 5 ppm with a spectral width of 10 ppm. 256 scans/FID, 200 ms acquisition time and a relaxation delay of 1.5 s were employed. In the indirect dimension (t_1, ^{13}C) the ^{31}P carrier was positioned at 0 ppm with a spectral width of 100 ppm, along with acquisition times of 6.3 ms. The *J* constant was set to 25 Hz.

References

(1) Ciuffreda, P.; Casati, S.; Manzocchi, A. Complete (1)H and (13)C NMR Spectral Assignment of Alpha- and Beta-Adenosine, 2'-Deoxyadenosine and Their Acetate Derivatives. *Magn. Reson. Chem.* **2007**, *45* (9), 781–784.
